# Anomalous Water Fluorescence Induced by Solutes

**DOI:** 10.1021/acs.jpclett.5c01189

**Published:** 2025-06-30

**Authors:** Anna Maria Villa, Jessica Di Paola, Diletta Ami, Luca De Gioia, Antonino Natalello, Luca Bertini

**Affiliations:** Department of Biotechnologies and Biosciences, 9305 University of Milano-Bicocca, Piazza della Scienza 2, 20126 Milan, Italy

## Abstract

The origin of recently reported anomalous fluorescence emissions from aqueous solutions of nonaromatic solutes remains elusive. To determine whether the solute nature influences the fluorescence characteristics and to identify a potential common mechanism, we measured the fluorescence spectra of 21 different solutions. We observed similar emission characteristics across all samples, suggesting that the solute nature plays a minimal role in the emission mechanism. Using time-dependent density functional theory on large water, NaCl/water, and glycerol/water clusters, we attributed the anomalous emission to the decay of charge-transfer-to-solvent excitations (CTTS) which populate a diradical zwitterionic excited state localized at hydrogen-bond network defects. The Arrhenius-like plots for NaCl and glycerol solutions revealed that the S_1_ nonradiative decay pathway involves the diradical recombination via librational motion. We propose that the presence of solute molecules slows this process, thus increasing the lifetime of the CTTS excited states and facilitating emission.

## Graphical abstract

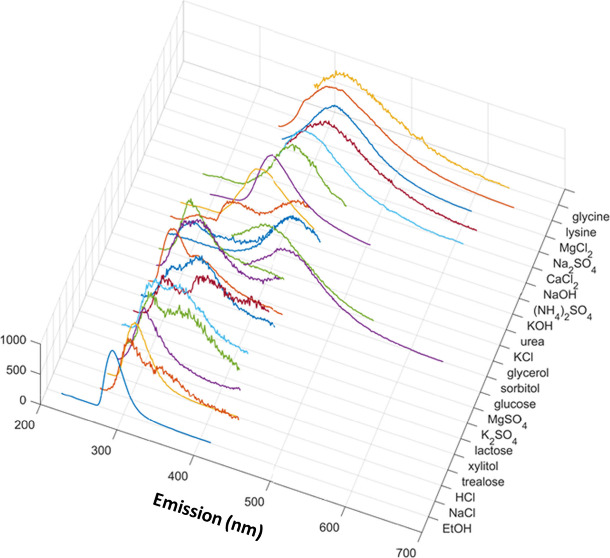

The peculiar properties of water are often referred to as anomalous or mysterious in the scientific literature.^1^ Many of these properties arise from the intricate and extended hydrogen bond network of liquid water, whose fluctuations are correlated to proton transfer and local electron density rearrangements.^2–5^ In the last two decades, many approaches have been proposed to model the dynamics of the water hydrogen bond network and to investigate how it correlates with the anomalous properties of the solutions.^6–9^ Such anomalies have been closely linked to the presence of inhomogeneities in the solution or defects in the structure of the hydrogen bonding network at the nanoscopic and mesoscopic level.^10^

Spectroscopic studies have played an important role in this field, especially in exploring the dynamics of water around solutes and in understanding how the chemical characteristics of solutes can impact such dynamics.^11^

Recently, many studies have been devoted to the intrinsic fluorescence of aqueous solutions of nonaromatic molecules, i.e., systems that lack the typical conjugated π-electron aromatic groups.^12–15^ This anomalous fluorescence is observed in the near UV and visible range and has been attributed to various mechanisms, such as charge transfer (CT) recombinations, hydrogen bonding stiffening, or aggregation-induced effects,^16^ that prolong the lifetime of the excited state, minimizing the probability of the nonradiative transitions from the S_1_ excited state to the ground state.^17^

The involvement of the hydrogen bond network in the mechanism of the anomalous fluorescence in aqueous solution suggests that the charge-transfer-to-solvent (CTTS) states and their evolution^18–21^ play an important role in these emissions. Indeed, CTTS bands show exceptional sensitivity to the surrounding water environment.^22^ In iodide aqueous solutions studied via ultrafast fluorescence spectroscopy, the CTTS state electronic structure depends on the local solvent structure, particularly on voids in the first solvation shell of the I^–^ ion.^22,23^ Anomalous fluorescence has been also observed in nonaromatic amino acids, peptides, monomeric proteins, and protein aggregates,^12–15,24–26^ with increased structural rigidity likely being the underlying cause,^27,28^ as in the case of amyloid aggregates.^19,20^ Recent studies suggest that anomalous fluorescence in proteins and peptide aggregates arises from H-bonds inhibiting CO bond stretching, slowing relaxation to the ground state, and extending the S_1_ excited state lifetime.^28,29^ Similarly, fluorophores composed solely of water molecules has been proposed to explain the anomalous fluorescence emission of water confined within nanocavities.^30^

In the investigation of these anomalous emissions, quantum chemistry modeling can be a crucial tool, in particular by identifying which atoms or atomic groups are involved in the excitation and in studying the fate of the excited state when the nonradiative decay is slowed or inhibited, thereby promoting emission.

In our previous papers,^24,25^ we investigated the anomalous fluorescence emission of HCl and KCl aqueous solutions, and we found emission bands with low quantum yield around 300 nm and in the 400–430 nm region, with excitation at 220–240 nm. To explain this anomalous fluorescence, we proposed a model based on time-dependent density functional theory (TD-DFT) computations where the nonradiative recombination dynamics is slowed down by the interaction of ions with the solvent, favoring emission. In the case of HCl, we observed that H_3_O^+^ ions contribute to emission by reducing the vibrational flexibility of the solutions, whereas for KCl, the ions are able to slow the dynamics of the H-bond networks, favoring emissive phenomena. These results raise an important question: do these observations hold only for 1:1 strong electrolytes or would they also apply to covalent solutes?

In order to assess whether the nature of the solute could affect the characteristics of this anomalous fluorescence and to identify a possible common mechanism, in this work we examine a wide range of solutes of different chemical natures, from covalent to ionic, showing that the emission features of their aqueous solutions are similar. This similarity suggests an underlying molecular mechanism primarily related to water relaxation dynamics. Using TD-DFT calculations on extended NaCl and glycerol–water cluster models, we characterize the CTTS excitation responsible for this anomalous fluorescence and elucidate its nonradiative decay mechanism providing a solid rationale for why all these fluorescence features fall within a relatively narrow range of wavelengths.

In this paper, we investigated 21 solutes in addition to Milli-Q water. All solutes are not considered fluorophores, since they do not present the typical characteristics of fluorescent molecules, i.e., delocalized electrons and rigid planar structures. Among these solutes, 11 are ionic and 10 are covalent molecules, thus comprising a broad range of water-soluble compounds. We included acid and bases (HCl, NaOH, KOH); 1:1 salts (KCl, NaCl); 1:2 salts (CaCl_2_, MgCl_2_, MgSO_4_); 2:1 salts (K_2_SO_4_, Na_2_SO_4_, (NH_4_)_2_SO_4_); nonaromatic amino acids (l-lysine, l-glycine); carbohydrates (lactose, glucose, trehalose); alditols (xylitol, sorbitol, glycerol); and diamide and alcohol (urea and ethanol). All the absorption, excitation, and emission spectra as a function of concentration and the emission spectra as a function of temperature are provided in the Supporting Information (SI, Figures S1 to S63).

We confirmed that pure liquid water has no detectable emission in the range of wavelengths considered (SI, Figures S1–S2), indicating that the S_1_ → S_0_ decay is a nonradiative process. In contrast, all of the aqueous solutions examined exhibited fluorescence emission. Figure 1 graphically reports the excitation and emission maxima, with the coordinates of each spot representing the wavelength of the emission and excitation maxima for each solute. Surprisingly, we found that the spots were not evenly distributed. Indeed, in emission, they cluster in three regions 20–40 nm wide centered at 295, 343, and 420 nm, while in excitation they cluster in two regions of about 30 nm wide centered at 228 and 323 nm.

**Figure 1:**
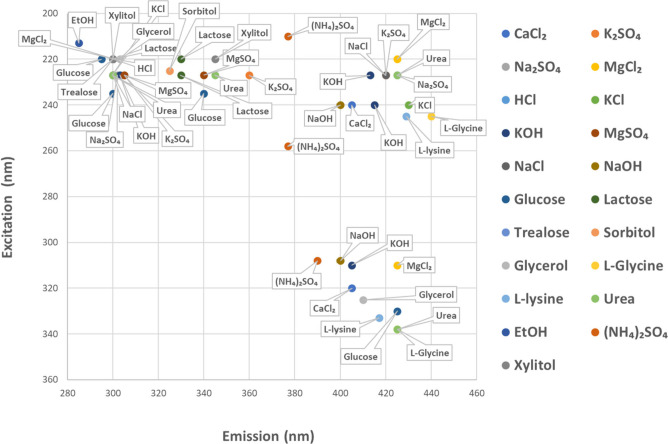
Comprehensive scatter plot displays the wavelength values for the maxima of the excitation and emission bands of all the aqueous solutions considered in this paper. The emission maxima cluster in three spectral regions centered at 295, 343, and 420 nm, while the excitation maxima cluster in two regions centered at 228 and 323 nm.

Among the various solutes considered, only the fluorescence properties of lysine were previously documented,^15^ and our results are consistent with those reported in the literature. As shown in Figure 1, strong electrolytes emit mainly in the region between 285 and 310 nm and in the region between 400 and 440 nm, while covalent solutes present emission bands that are almost evenly distributed in the three regions. The observation that all the aqueous solutions emit within a narrow wavelength range (280–440 nm) suggests that the molecular mechanism could be a shared nonradiative decay pathway, likely related to the aqueous solvent rather than the solute. This mechanism appears to be slowed down compared to pure water, thereby promoting emission—albeit with low quantum yields.

For 17 selected solutes, we determined the relative quantum yield (QY) (SI Table S1). By averaging over all the measured QY values, we obtain a mean of 0.008 ± 0.002. Clustering the QY on the basis of the three excitation regions and taking the average for each region, we obtain 0.006 ± 0.005 (220–227 nm), 0.008 ± 0.007 (240–260 nm), and 0.010 ± 0.003 (>260 nm) (SI, Figure S64).

NaCl and glycerol (hereafter referred to as Gly in molecular formulas), as model systems of ionic and covalent solutes, have been further extensively investigated through spectroscopic analysis and TD-DFT modeling. For both solutions, we aim to provide a detailed characterization of the nonradiative decay mechanisms of the S_1_ state, as their slowdown promotes fluorescence emission.

In Figure 2A, we report the absorption spectra of NaCl aqueous solutions at different concentrations from 0.25 to 4 M, showing two shoulders at 227 and 240 nm. For comparison, the absorption spectrum of water is also reported in light blue.

**Figure 2:**
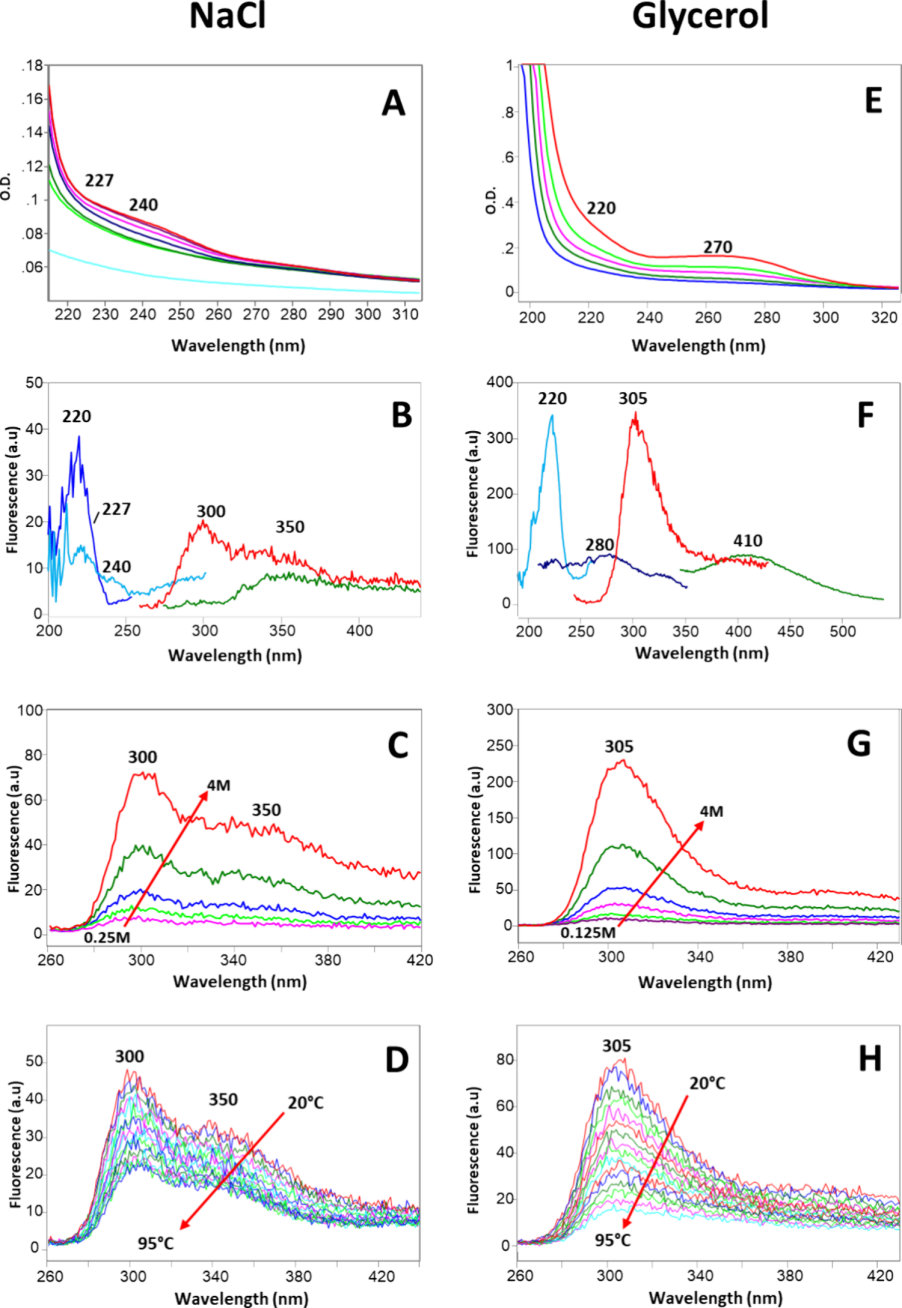
(A) Absorption spectra of NaCl aqueous solutions at different concentrations (4 M, 2 M, 1 M, 0.5 M, 0.25 M), the absorption spectrum of water is reported for comparison (light blue); (B) excitation and emission spectra of 1 M NaCl solution (excitation: blue = emission at 300 nm, light blue = emission at 350 nm; emission: red = excitation at 227 nm, green = excitation at 240 nm); (C) fluorescence emission spectra of NaCl aqueous solutions at different concentrations (4 M, 2 M, 1 M, 0.5 M, 0.25 M); excitation at 227 nm; (D) fluorescence emission spectra of 1 M NaCl aqueous solution at different temperatures; the emission spectra are recorded every 5 °C from 20 to 95 °C; excitation at 227 nm; (E) absorption spectra of glycerol aqueous solutions at different concentrations (4 M, 2 M, 1 M, 0.5 M, 0.25 M); (F) excitation and emission spectra of 1 M glycerol solution (excitation: light blue = emission at 305 nm, blue = emission at 410 nm; emission: red = excitation at 220 nm, green = excitation at 280 nm); (G) Fluorescence emission spectra of glycerol solutions at different concentrations (4 M, 2 M, 1 M, 0.5 M, 0.25 M, 0.125 M); excitation at 220 nm; (H) fluorescence emission spectra of 1 M glycerol aqueous solution at different temperatures; the emission spectra are recorded every 5 °C from 20 to 95 °C with excitation at 220 nm. Conditions: photomultiplier gain in B–D, F: 900 V; in G–H 770 V. Representative spectra from three independent experiments are shown.

The fluorescence excitation and emission spectra of a 1 M aqueous solution of NaCl are reported in Figure 2B. The excitation spectrum (blue), collected with the emission centered at 300 nm, presents a peak at 220 nm and a shoulder at 227 nm. The excitation spectrum with emission centered at 350 nm (light blue) shows a peak at 220 nm with a shoulder at 240 nm. Upon excitation at 227 nm, two bands are observed in the emission spectrum (red): a strong emission peaked at 300 nm and a weaker emission peaked at 350 nm. By exciting at 240 nm, the emission spectrum (green) only shows a large weak band with a maximum at 350 nm. Figure 2C shows the fluorescence emission spectra of NaCl solutions at different concentrations from 0.25 to 4 M. The emission intensity of the band peaked at 300 nm (exc = 227 nm) increases with the solute concentration, suggesting that the anomalous fluorescence is due to the presence of the solute.

From the absorption and emission spectra of NaCl solution, we determined a relative QY of 0.004 ± 0.003 for the strong emission band at 300 nm with excitation at 227 nm (SI, Table S1 and Figure S64).

The absorption spectra of glycerol aqueous solutions at different concentrations from 0.25 to 4 M are displayed in Figure 2E, showing a shoulder at 220 nm and a large band at 270 nm.

In Figure 2F we report the fluorescence excitation and emission spectra of a 1 M solution of glycerol. The excitation spectrum collected with emission at 305 nm (light blue) shows a single strong peak at 220 nm, while the excitation spectrum with emission at 410 nm (blue) presents a low intensity broad band with a maximum at 280 nm. Upon excitation at 220 nm, a strong emission peak at 305 nm is observed (red), while excitation at 280 nm gives a broad emission band at 410 nm (green). Figure 2G shows the fluorescence emission spectra of glycerol solutions from 0.125 to 4 M. The emission intensity at 305 nm (exc = 220 nm) increases with concentration, confirming that this anomalous fluorescence is due to the presence of the solute. The strong 305 nm band has a relative QY of 0.004 ± 0.002. As seen in Figure 2C (NaCl) and 2G (glycerol), the emission maximum remains unchanged, indicating consistent structural fluorophores across concentrations.

The effect of temperature on the fluorescence intensity of the NaCl solution was investigated by collecting the fluorescence emission spectra every 5 °C from 20 to 95 °C (Figure 2D). As expected, the fluorescence intensity decreases with increasing temperature (Figures S65–S66).

Indeed, as the temperature increases, the nonradiative deactivation pathways become more efficient, leading to a decrease in fluorescence intensity described as *e* ^–*E*_a_/*RT*^. By measuring the temperature dependence of fluorescence intensity, we estimated the activation energies (*E*_a_) of competing nonradiative processes^25,31,32^ via an Arrhenius-like plot. Here, the slope of the Arrhenius plot represents the activation energy for the nonradiative S_1_ → S_0_ decay along the S_1_ potential energy surface. The value of *E*_a_ is an estimate of how much energy is required to activate the thermal quenching process on the S_1_ PES, providing insight into its possible molecular mechanism. A higher energy barrier corresponds to slower nonradiative decay and thus to an increased probability of fluorescence emission.^29^

For the 1 M NaCl solution, the Arrhenius plot is linear (see SI, Figure S69), yielding an *E*_a_ value of 9.6 ± 0.8 kJ/mol, which corresponds to 802 ± 63 cm^–^^1^ in wavenumbers, a frequency within the region of water librations, as shown by the FTIR spectra of NaCl solutions (SI, Figures S67, S68). This result suggests that a libration mode—the intermolecular vibration bands ranging from 500 to 1000 cm^–^^1^ that peak around 600–700 cm^–^^1^—could be a strong candidate for the mode slowed down or hindered by NaCl and potentially involved in the nonradiative decay of the S_1_ state.^33^ Indeed, this value of activation energy is in reasonable agreement with the value of ∼10 kJ/mol that is the energy required to break a hydrogen bond via librational motion.^34^

Considering glycerol, in the case of the 305 nm band (exc = 220 nm) of the 1 M solution (Figure 2H), the Arrhenius plot is significantly different from NaCl, showing a nonlinear trend (SI, Figure S70), which can be interpreted as the presence of multiple fluorescence inactivation processes^35^ in the examined temperature range. By breaking the plot into two sections, each linearly interpolated, we obtained two values of activation energies. For temperatures below 60 °C, we found *E*_a_ = 11.5 ± 0.4 kJ/mol, a value that is in agreement with the calculated energy barrier of 12–13 kJ/mol between strong and weak hydrogen bonds in water.^36,37^ Its wavenumber value corresponds to 959 ± 25 cm^–1^, an infrared frequency in the region of water librations.^33^ It is interesting that for both NaCl and glycerol, this frequency is higher than that of the libration band of bulk liquid water (centered at 670 cm^–1^ at 25 °C), indicating a stiffening of the OH rotational potential.^33^ Above 60 °C, a new degree of freedom is gained, as indicated by the slope change in the Arrhenius plot. The new value of the activation energy is 20.4 ± 2.4 kJ/mol, in agreement with the activation energy for a conformational transition of glycerol.^31,38,39^

Table S2 in SI summarizes the activation energies obtained for NaCl, glycerol, HCl, KCl, and l-lysine. On average, except for glycerol, when compared with the vibrational spectrum of liquid water, these wavenumbers are in the region of the librational modes.^40^ We considered a second case of a covalent solute, namely l-lysine, which has an activation barrier of 8.7 ± 0.4 kJ/mol, once again in line with the values reported above.

To investigate fluorescence mechanisms, we performed TD-DFT calculations (for further validation, see SI for details). We consider (H_2_O)_110_, (NaCl)_2_(H_2_O)_108_, and (Gly)_2_(H_2_O)_108_ that mimic bulk liquid water and 1 M NaCl and glycerol solutions, providing a realistic yet compact representation of the bulk phase. Additionally, the literature indicates that the infrared spectra of water clusters comprising 10–100 molecules resemble those of the bulk liquid water.^41^ Therefore, studying solute-water clusters with a similar number of solvent molecules could be representative of the behavior of a real bulk solution.

Compared to the TD-DFT modeling proposed in our previous works,^24,25^ the novelty of the approach here is to consider clusters that are twice as large (110 water molecules versus 55). This allowed us to keep the structure fully relaxed during the TD-DFT geometry optimization without observing the unphysical dissociations of hydrogen atoms from the surface of the cluster, which had previously forced us to impose constraints on the atomic positions at the cluster surface. In contrast, the larger cluster size enabled better delocalization of the molecular orbitals involved in excitation, thereby preventing these dissociations.

This study is structured as follows: (i) a pool of representative cluster structures was selected and subsequently optimized at the ground-state DFT level, starting from a molecular dynamics (MD) trajectory (see computational details in SI); (ii) S_1_ excitation energy and bands assignment via TD-DFT; (iii) nonradiative decay of S_1_ simulated via TD-DFT geometry relaxation to propose the fluorescent emitter’s structure and mechanism.^42^

Before analyzing the results, it is important to clarify that the DFT-level sampling of the PES for water and solute/water clusters, aimed at locating minimum-energy geometries, is not intended to comprehensively capture the dynamical behavior of the actual system. The goal is not to identify the global minimum but rather to determine a set of representative local minimum structures with varying water/solute arrangements. These structures serve as starting points for assigning the CTTS band to explore the dynamics on the S_1_ state’s PES.

We first considered the (H_2_O)_110_ water cluster.^43,44^ The results of the DFT geometry optimization of 20 structures obtained from classical MD simulations are reported in Figure 3 and in SI Table S3. On average, the selected structures are characterized by 191 ± 2 H-bond interactions with an O–H distance of 1.82 ± 0.01Å.

**Figure 3:**
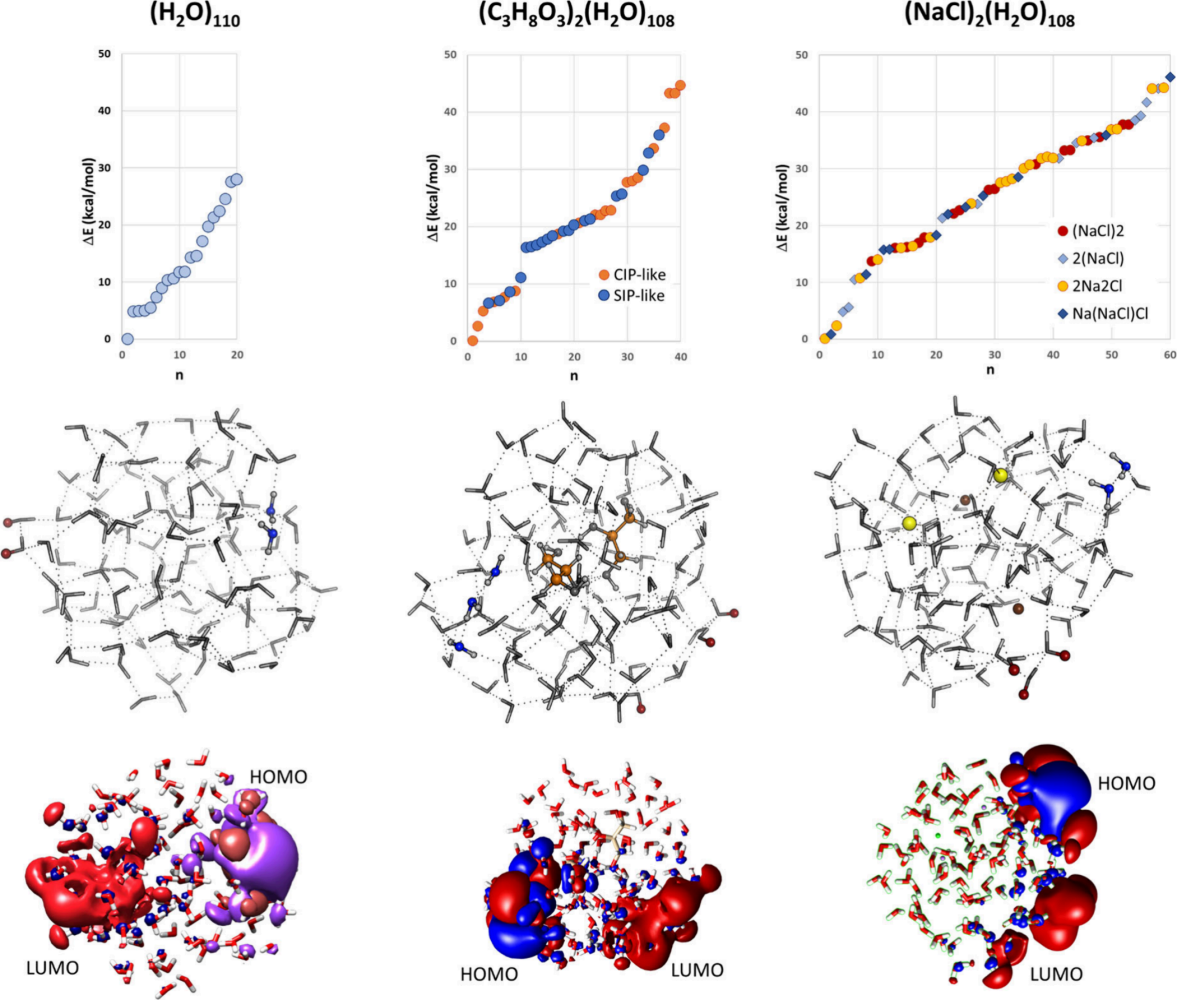
At the top of the figure: the stability of the cluster model is considered with respect to the lowest energy structure identified. Δ*E* is the B-LYP/TZVP energy difference in kcal/mol. In the case of the glycerol water clusters, in blue and orange we report the solvent separated-like (SIP-like) and contact-pair like (CIP-like) forms in which one or more water molecules are or are not interposed between the two glycerol molecules. In the central part of the figure we report the structures for the most stable form (Δ*E* = 0 kcal/mol). The oxygen atoms and the hydrogen atoms involved in the CTTS band are evidenced in blue and red, respectively. In the bottom the HOMO and LUMO orbitals are reported for the most stable geometry form.

The S_1_ excitation involves a HOMO → LUMO monoelectronic transition with an average excitation wavelength equal to 268.8 ± 13.9 nm. Based on MO populations, HOMO is always localized on oxygen atoms that belong to water molecules at the cluster surface, with the main contribution from 2p lone-pair orbitals. In these structures, the water molecules on the cluster surface are typically double donor (both their hydrogen atoms are involved in hydrogen bonding) but single acceptor (their oxygen atoms are single acceptor of one H-bond), following the nomenclature recently proposed.^45^ Given that the 2p lone-pair based MO are stabilized through the formation of hydrogen bonds, it becomes highly probable^46^ that the HOMO within any finite-size cluster of water molecules is *predominantly localized on the cluster’s surface*. Similarly, LUMO major orbital contributions come from “dangling” hydrogen atoms (i.e., not involved in the formation of hydrogen bonds) that belong to surface water molecules pointing toward the outside of the cluster.^47^

Figure 3 shows the structure of the most stable (H_2_O)_1__1__0_ cluster identified, along with the HOMO and LUMO plots highlighting the nature of the S_1_ charge CT state.

According to natural bond order (NBO) populations, S_1_ excitation has an O→H CT character (see Table S6 in SI). On average, 0.83 electrons are transferred from one oxygen atom, with atomic orbitals contributing to the HOMO, to a certain number of hydrogen atoms that belong to LUMO. Based on this analysis, we describe this CT state as a peculiar zwitterionic diradical (H_2_O)^•+^/(H_2_O)^•–^ form, in which the electron vacancy and the excess electron are localized on the surface of the cluster and tend to be as far apart as possible in order to minimize the attractive Coulomb interaction that destabilizes the CT state. The electron vacancy is associated with the HOMO; excess electrons are associated with the LUMO, primarily contributed by “dangling” hydrogen atoms at the cluster edge, similar to the double H-bond acceptor motif in smaller (H_2_O)_*n*_^–^ clusters.^48–51^

After S_1_ excitation, charge recombination occurs. To investigate its dynamics, we carried out S_1_ TD-DFT geometry optimization. Starting from the S_0_ minimum forms, we observe (Figure 5A) the rapid reaching of the S_1_/S_0_ crossing, with the formation of the hydrogen-bonded hydronium-hydroxyl radical contact (H_3_O)^+^···(HO)^•^ unit which involves the oxygen atoms contributing to the HOMO of the S_0_. This result supports our assignment of S_1_ as a zwitterionic diradical-like species. Indeed this behavior is in line with the investigations^52,53^ of the small cationic water cluster (H_2_O)_*n*_^**•**+^ in which the lowest energy isomer is an hydrogen-bonded system (H_3_O)^+^···(HO)^**•**^, while the hemibonded system (H_2_O···H_2_O)^**•**+^ is higher in energy.^54^

In the cluster region with excess charge (H_2_O)^**•**‑^, vibrational relaxation on S_1_ minimally alters the molecular geometry surrounding the negative charge. After S_1_/S_0_ crossing, back electron transfer occurs from (H_2_O)^**•**−^ to (H_3_O)^+^···(HO)^•^, followed by back proton transfer, which leads to the initial form.

In the case of (NaCl)_2_(H_2_O)_108_ and (Gly)_2_(H_2_O)_108_, we considered structures with different ion/molecule pairing (see Figure 5 and Figures S69–S70)

The electronic structure of NaCl water clusters and ion solvation have been widely studied at DFT level, while classical or ab initio MD^55–60^ have been used to investigate the early stage of salt dissolution/salt precipitation. Here we selected 60 (NaCl)_2_(H_2_O)_108_ model structures extracted from a classical MD trajectory. (Table S4 in SI). On average these structures are characterized by (i) 179 ± 4 H-bond interactions with an O–H distance of 1.82 ± 0.11 Å; (ii) Na^+^ and Cl^–^ coordination numbers equal to 4.5 ± 0.5 and 5.2 ± 0.7 (Figure 4B). The Na^+^···Cl^–^ average distance is 5.32 ± 1.52 Å in line with the second peak of the radial distribution function at 5.1 Å, which corresponds to a solvent separated ion-pair;^61,62^ (iii) Na^+^···Na^+^ and Cl^–^···Cl^–^ are 6.25 ± 1.83 Å and 7.94 ± 3.42 Å respectively (Figure 4C).

**Figure 4:**
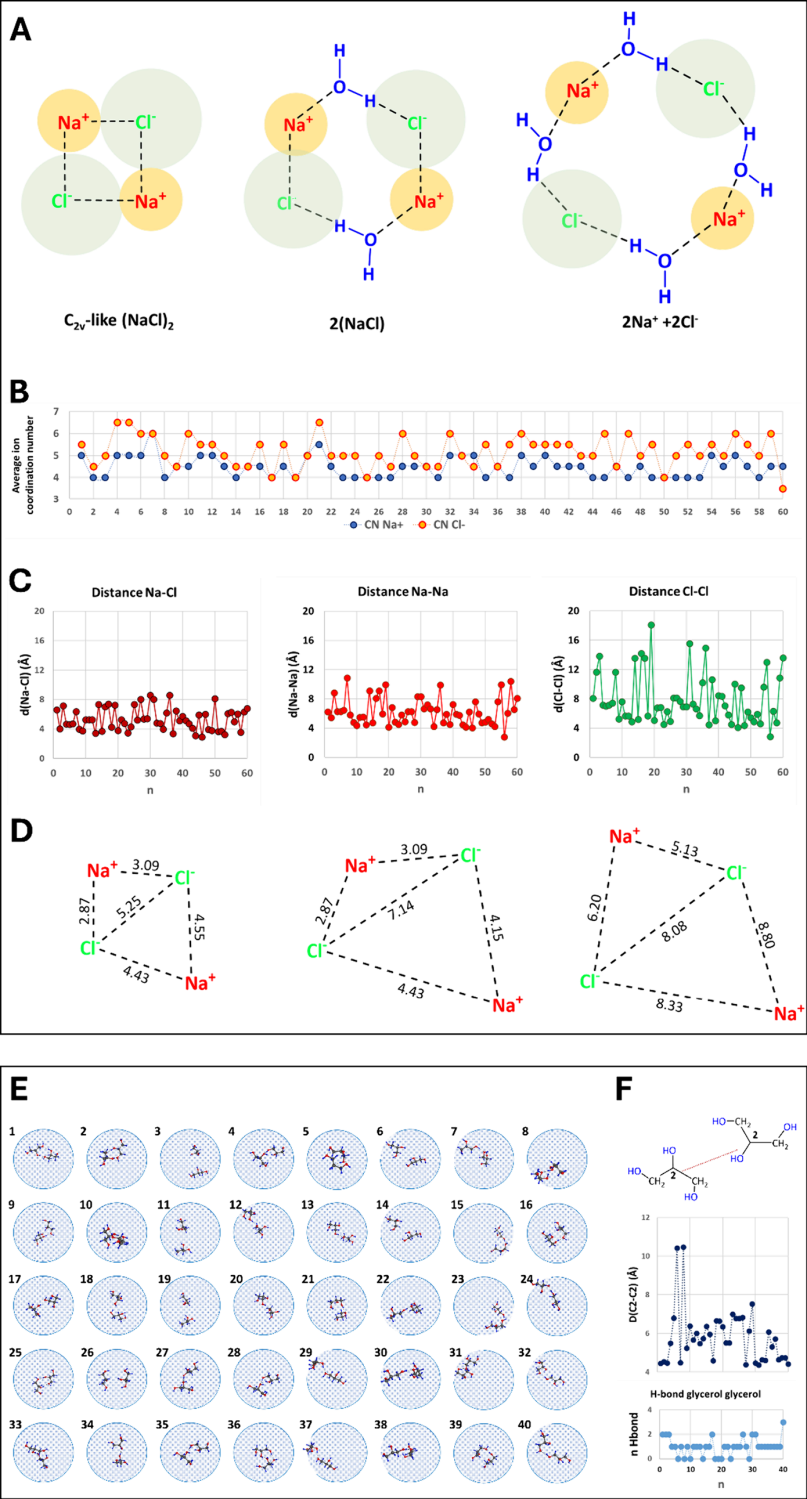
(A) The three (NaCl)_2_(H_2_O)_108_ cluster forms considered in the minimum energy search: contact ion pairs (C_2v_-like (NaCl)_2_), two CIP pairs separated by water (2­(NaCl)), and solvent-shared ion pairs (2Na^+^ + 2Cl^–^). (B) Na^+^ and Cl^–^ coordination numbers (threshold 4 Å). (C) Average ion–ion distances (Å). (D) Lowest-energy structures of each type. (E) Sketches of 40 (Gly)_2_(H_2_O)_108_ isomers, highlighting glycerol–-glycerol H-bonds. (F) C2–C2 distances vs number of H-bonds, sorted by stabilization energy.

According to Figure 4A, our (NaCl)_2_(H_2_O)_108_ selection includes 21 SSIP-like, 16 CIP, and 23 structures with two CIP pairs separated by water. Figure 4D and Figure S71 (SI) show the most stable structures, with the lowest-energy one being SSIP. Shorter hydrogen bonds generally correlate with lower energy.

The HOMO → LUMO S_1_ has an average excitation wavelength equal to 277.2 ± 14.0 nm. In the majority of the structures considered (43 out of 60), the HOMO involves water molecules at the cluster surface with a main contribution from the O 2p and/or Cl^–^ 3p lone-pair orbitals. LUMO always has Na^+^ 3s orbital contributions, plus, possibly, “dangling” hydrogen atoms. Therefore, according to HOMO/LUMO populations, the S_1_ state has Cl, O → Na, H CT character. On average 0.6 electrons are transferred from O or Cl atoms to H or Na atoms, where electronic excess is more delocalized compared to electronic vacancy. Depending on the HOMO orbital contributions, we observe S_1_ bands with a prevalent (i) solvent-to-solute CT character in which diradical forms like (H_2_O)^•+^/Na^•^ results transiently populated (when Cl^–^ is not involved in HOMO); (ii) solute-to-solute or solute-to-solvent CT character^63^ in which structures of type Cl^**•**^/(H_2_O)^**•**‑^ or type Cl^**•**^/Na^**•**^ results transiently populated.

S_1_ dynamics in NaCl clusters is studied via TD-DFT geometry optimization from the S_0_ minimum (Figure 5B and Figure S72 in SI). On average, S_1_/S_0_ crossing occurs after 74 cycles, sometimes reaching a near-stationary point. In most cases, nonradiative S_1_ decay involves intermolecular motions similar to those in water, often forming an (H_3_O)^+^···(HO)^•^ pair. When chloride is the donor in the CTTS band, during S_1_ vibrational cooling, we observe its displacement along with its coordinated water molecules.

**Figure 5:**
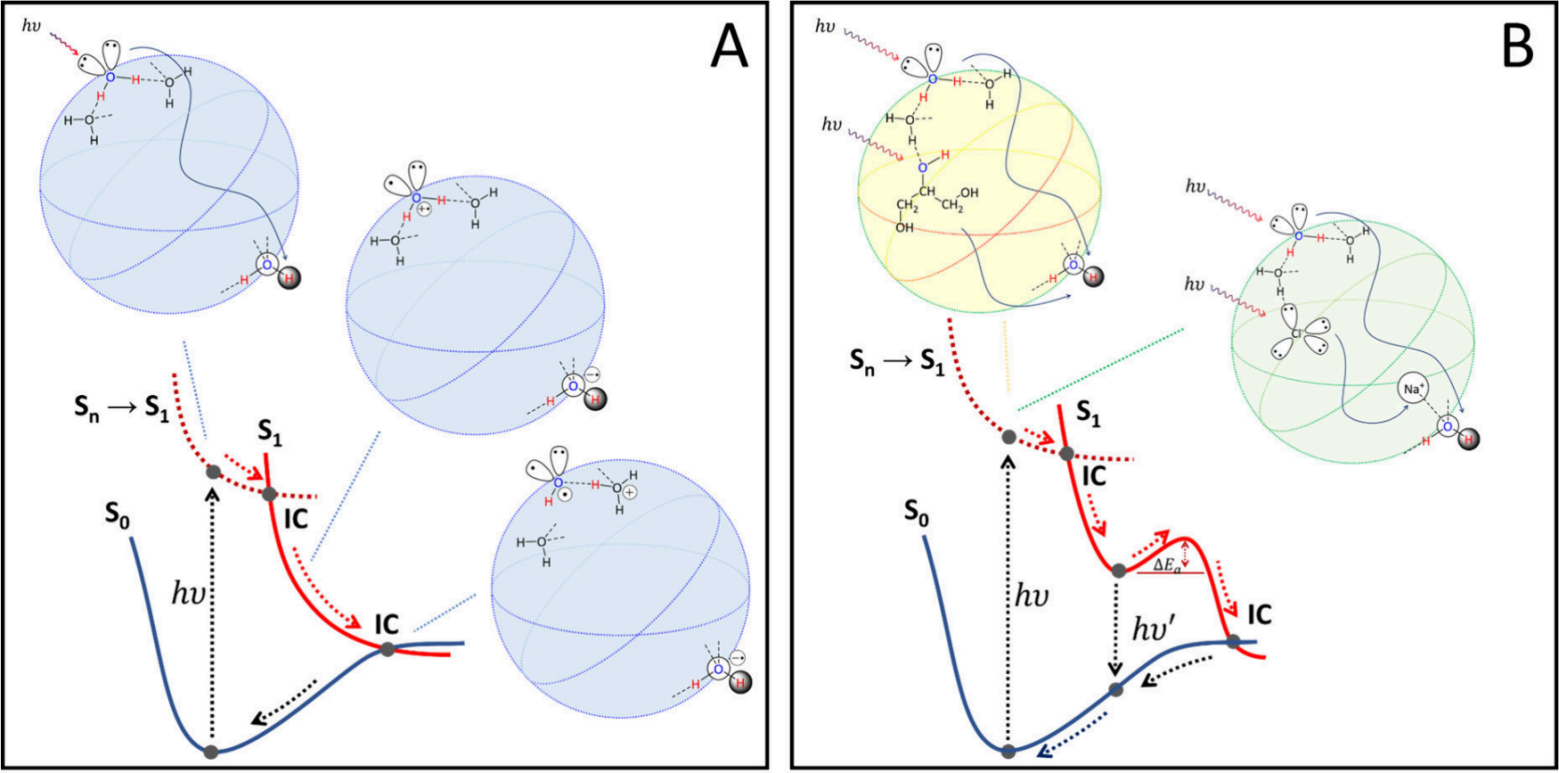
Scheme of CTTS S_1_ excitation and radiationless decay to S_0_ for (H_2_O)_110_ (A, blue spheres) and (NaCl)_2_(H_2_O)_108_ and (Gly)_2_(H_2_O)_108_ model clusters (B, green and yellow sphere, respectively). Dotted red PES represent the S_*n*_ excited PES with *n* > 1. After light absorption, a S_*n*_ excited state is populated and undergoes vibrational cooling decay to S_1_, passing through S_*n*_ → S_*n*‑1_ intermolecular internal conversion (IC). For water clusters (A), S_1_ is characterized by an HOMO → LUMO CT transition localized at the cluster surface, with the formation of a zwitterionic diradical H_2_O^•+^/H_2_O^•–^ solvated species. This latter evolves by vibrational cooling until reaching S_1_ → S_0_ IC. At this point, the solvated H_2_O^•+^/H_2_O evolve to a H_3_O^+^/OH^•^ ion-radical contact. On the S_0_ PES, this final species evolves rapidly by charge/radical recombination to the initial minimum geometry. In the case of glycerol (in light yellow) the CTTS state involves an HOMO → LUMO CT transition that is localized at the cluster surface, with the formation of a H_2_O^•+^/H_2_O^•‑^ or Gly^•+/^H_2_O^•–^ solvated species; in the case of NaCl (in light green) TDDFT shows the formation of H_2_O^•+^/Na^•^ or Cl^•/^Na^•^ solvated species.

As a representative example of a covalent solute, we investigated glycerol water clusters. Glycerol, a key cryoprotectant for living cells, has been extensively analyzed for its impact on water H-bonding.^64–67^ Most computational investigations use MD approaches to elucidate the H-bond dynamics of glycerol/water mixtures,^68–70^ while few focus on clusters, mainly in photoionization mass spectrometry.^71^

40 (Gly)_2_(H_2_O)_108_ model structures were selected from a classical MD trajectory and minimized at the DFT level (see Figure 4E-F and Tables S5–S6 in SI). On average, these structures are characterized by 203 ± 3 H-bond interactions with an average O–H distance of 1.824 ± 0.112 Å and C2–C2 distance between the two Gly molecules of 5.31 ± 1.41 Å, where in the case of the lowest energy form is 4.45 Å. As in the case of (NaCl)_2_(H_2_O)_108_ structures, the lower-energy Gly clusters generally have shorter H-bond distances.

S_1_ excitation involves an HOMO → LUMO transition with an average energy of 273.0 ± 13.7 nm and an O→H CT character, except in some structures where HOMO contributions come from one glycerol molecule. LUMO is always localized on the cluster’s surface “dangling” hydrogen atoms. On average, 0.5 electrons transfer from a few oxygens to several hydrogens, indicating greater delocalization of electronic excess than vacancy. S_1_ dynamics, explored via TD-DFT optimization (Figure 5B), resemble those in NaCl/water clusters, with S_1_/S_0_ crossing occurring after 65 cycles.

Although TD-DFT calculations on finite clusters cannot directly capture the dynamics of bulk liquid water, our results suggest that molecules involved in CTTS transitions are often found in environments that deviate from ideal tetrahedral coordination. In the liquid phase, thermal fluctuations continuously lead to transient local structures with incompletely hydrogen-bonded water molecules. These molecules transiently involved in local environments that are not fully tetrahedral can act as potential donor–acceptor pairs contributing to charge-transfer bands in the absorption spectrum.

Depending on their structure, these defects act as donor/acceptor centers for a CTTS band, leading to the formation of a (H_2_O)^•+^/(H_2_O)^•–^ zwitterionic diradical state, which evolves nonradiatively by recombining back to the ground state. Although these observations cannot be considered exhaustive, they clearly suggest that the decay dynamics of S_1_ for liquid water is nonradiative, in agreement with our experimental findings.

Our TD-DFT analysis suggests that the same type of CTTS excitation found in water is responsible for the emission observed in aqueous solutions. Here, CTTS excitation involves either undercoordinated water molecules at local defects (where the tetrahedral ordering of the hydrogen-bond network is perturbed by the presence of solute molecules) or the solute molecules themselves. In either case, a diradical excited state associated with hydrogen-bonding network defects is populated. This leads to an important question: beyond the ordinary molecular dynamics of the species in solution, what additional mechanisms could contribute to the formation of such defects within the bulk?

As highlighted in numerous studies, defects in tetrahedrality are observed in high-density water.^1–5^ Indeed, water exhibits a low-density phase with a well-ordered tetrahedral structure and a less structured high-density phase, where the hydrogen bond network becomes more disordered, leading to a significant reduction in tetrahedrality. Molecular modeling studies of the microscopic structural features of pure water have identified locally favored structures characterized by a peculiar arrangement of long-lived hydrogen bonds.^72,73^ These structures can be defined by their propensity to form local tetrahedral-type or distorted-type patches with lower and higher density.^72,74–77^ Interestingly, by MD simulations^42,72–74,78^ transient dendritic or fractal-like voids have been identified in pure water, where water molecules at the interface of voids are organized in high density patches. Since the surface of our water model cluster can be seen as the interface between bulk solution and an empty region like a void, we suggest that the diradical CTTS excited states we characterized at the TD-DFT level might be localized at the surface of these voids. In the case of pure water, this state evidently recombines in an ultrafast time scale, and no emission is therefore observed.

In conclusion, our study shows that the 21 aqueous solutions considered exhibit fluorescence with a common emission mechanism that involves a CTTS state localized mainly on hydrogen bond defects with transient formation of a diradical species. This CTTS state decays in pure water via nonradiative pathways, and no emission is observed, whereas for solutions this fast nonradiative decay is partially suppressed. It is therefore possible to hypothesize that the effect of the solute molecules could be to lengthen the lifetime of CTTS states by stiffening the hydrogen bond network around the local hydrogen bond defects, thus favoring fluorescence emission. In this perspective, the solute only plays a role in “activating” the fluorophore, which remains “switched off” in pure water. As a result, while some variance in the emissive properties of the solutions is present, it remains limited. Based on the observations reported, it might be reasonable to assume that this mechanism is common to many aqueous solutions. By aligning the wavenumber that best corresponds to the values obtained from Arrhenius-type plots, FTIR difference spectra, and the nuclear motions observed during TD-DFT S_1_ cooling, intermolecular librations appear to be a strong candidate for describing the mode responsible for the nonradiative decay of S_1_.

The results reported in this work offer a new perspective in the study of anomalous intrinsic fluorescence of aqueous solutions, showing how local defects in the H-bond network in water solutions can act as fluorophores.

## Supplementary Material







## Notes

DFT minimum-energy equilibrium structure Cartesian coordinates of the (H_2_O)_110_, (NaCl)_2_(H_2_O)_108_, and (C_3_H_6_O_3_)_2_(H_2_O)_108_ clusters (PDF)
Absorption and emission spectra of all the solutions considered but not reported in the main text (Figures S1–S63); determination of relative fluorescence quantum yields (Table S1 and Figure S64); fluorescence emission intensity of 1 M NaCl and glycerol solution vs temperature (Figures S65–S66); NaCl solution FTIR spectra (Figures S67–S68); Arrhenius plots for NaCl and glycerol 1 M solutions (Figures S69–S70); activation energy for radiationless decay of selected solutes (Table S2); computational results: classical mechanism and DFT/TD-DFT protocols; summary of structural and electronic properties for (H_2_O)_110_ water clusters (Table S3), (NaCl)_2_(H_2_O)_108_ clusters (Table S4) and (C_3_H_6_O_3_)_2_(H_2_O)_108_ (Table S5) including relative DFT energies, structural and hydrogen bonding informations, S_1_ excitation wavelength, main monoelectronic excitations and description of relaxation from TD-DFT calculations; general scheme for the description of (NaCl)_2_(H_2_O)_108_ structures (Figure S71); benchmarking CT and S_1_ absorption wavelength of water, NaCl and glycerol clusters as a function of DFT functionals; S_1_ and S_0_ recombination coordinates simulated using TD-DFT geometry optimization (Figure S72) (PDF)
Transparent Peer Review report available (PDF)

## Notes

In memory of Professor Silvia Maria Doglia, a passionate scientist and dear friend who played a central role in our study of water peculiar properties and made significant contributions in molecular and cellular biophysics.

## References

[1] Mallamace F. (2009). The Liquid
Water Polymorphism. Proc. Natl. Acad. Sci. U.
S. A..

[2] Hassanali A., Giberti F., Cuny J., Kühne T. D., Parrinello M. (2013). Proton Transfer through the Water Gossamer. Proc. Natl. Acad. Sci. U. S. A..

[3] Ceriotti M., Cuny J., Parrinello M., Manolopoulos D. E. (2013). Nuclear
Quantum Effects and Hydrogen Bond Fluctuations in Water. Proc. Natl. Acad. Sci. U. S. A..

[4] Nilsson A., Pettersson L. G. M. (2015). The
Structural Origin of Anomalous Properties of Liquid
Water. Nat. Commun..

[5] Huang C., Wikfeldt K. T., Tokushima T., Nordlund D., Harada Y., Bergmann U., Niebuhr M., Weiss T. M., Horikawa Y., Leetmaa M., Ljungberg M. P., Takahashi O., Lenz A., Ojamäe L., Lyubartsev A. P., Shin S., Pettersson L. G. M., Nilsson A. (2009). The Inhomogeneous Structure
of Water at Ambient Conditions. Proc. Natl.
Acad. Sci. U. S. A..

[6] Amann-Winkel K., Bellissent-Funel M.-C., Bove L. E., Loerting T., Nilsson A., Paciaroni A., Schlesinger D., Skinner L. (2016). X-Ray and Neutron Scattering
of Water. Chem. Rev..

[7] Perakis F., De Marco L., Shalit A., Tang F., Kann Z. R., Kuhne T. D., Torre R., Bonn M., Nagata Y. (2016). Vibrational
Spectroscopy and Dynamics of Water. Chem. Rev..

[8] Giovannini T., Egidi F., Cappelli C. (2020). Molecular Spectroscopy of Aqueous
Solutions: A Theoretical Perspective. Chem.
Soc. Rev..

[9] Cisneros G. A., Wikfeldt K. T., Ojamäe L., Lu J., Xu Y., Torabifard H., Bartók A. P., Csányi G., Molinero V., Paesani F. (2016). Modeling Molecular Interactions in
Water: From Pairwise to Many-Body Potential Energy Functions. Chem. Rev..

[10] Sedlák M. (2006). Large-Scale
Supramolecular Structure in Solutions of Low Molar Mass Compounds
and Mixtures of Liquids: I. Light Scattering Characterization. J. Phys. Chem. B.

[11] Heugen U., Schwaab G., Bründermann E., Heyden M., Yu X., Leitner D. M., Havenith M. (2006). Solute-Induced
Retardation of Water
Dynamics Probed Directly by Terahertz Spectroscopy. Proc. Natl. Acad. Sci. U. S. A..

[12] Tomalia D. A., Klajnert-Maculewicz B., Johnson K. A.-M., Brinkman H. F., Janaszewska A., Hedstrand D. M. (2019). Non-Traditional Intrinsic Luminescence: Inexplicable
Blue Fluorescence Observed for Dendrimers, Macromolecules and Small
Molecular Structures Lacking Traditional/conventional Luminophores. Prog. Polym. Sci..

[13] Niyangoda C., Miti T., Breydo L., Uversky V., Muschol M. (2017). Carbonyl-Based
Blue Autofluorescence of Proteins and Amino Acids. PLoS One.

[14] Morzan U. N., Díaz Mirón G., Grisanti L., González
Lebrero M. C., Kaminski Schierle G.
S., Hassanali A. (2022). Non-Aromatic
Fluorescence in Biological Matter: The Exception or the Rule?. J. Phys. Chem. B.

[15] Stagi L., Farris R., de Villiers
Engelbrecht L., Mocci F., Maria Carbonaro C., Innocenzi P. (2022). At the Root of L-Lysine Emission
in Aqueous Solutions. Spectrochim. Acta A Mol.
Biomol. Spectrosc..

[16] Mei J., Leung N. L. C., Kwok R. T. K., Lam J. W. Y., Tang B. Z. (2015). Aggregation-Induced
Emission: Together We Shine, United We Soar!. Chem. Rev..

[17] Díaz
Mirón G., Lien-Medrano C. R., Banerjee D., Morzan U. N., Sentef M. A., Gebauer R., Hassanali A. (2024). Exploring
the Mechanisms behind Non-Aromatic Fluorescence with the Density Functional
Tight Binding Method. J. Chem. Theory Comput..

[18] Barthel E. R., Martini I. B., Schwartz B. J. (2001). How Does the Solvent Control Electron
Transfer? Experimental and Theoretical Studies of the Simplest Charge
Transfer Reaction. J. Phys. Chem. B.

[19] Pinotsi D., Grisanti L., Mahou P., Gebauer R., Kaminski C. F., Hassanali A., Kaminski Schierle G.
S. (2016). Proton Transfer and
Structure-Specific Fluorescence in Hydrogen Bond-Rich Protein Structures. J. Am. Chem. Soc..

[20] Grisanti L., Pinotsi D., Gebauer R., Kaminski Schierle G. S., Hassanali A. A. (2017). A Computational Study on How Structure
Influences the
Optical Properties in Model Crystal Structures of Amyloid Fibrils. Phys. Chem. Chem. Phys..

[21] Barthel E. R., Martini I. B., Schwartz B. J. (2000). Direct
Observation of Charge-Transfer-to-Solvent
(CTTS) Reactions: Ultrafast Dynamics of the Photoexcited Alkali Metal
Anion Sodide (Na−). J. Chem. Phys..

[22] Messina F., Bräm O., Cannizzo A., Chergui M. (2013). Real-Time Observation
of the Charge Transfer to Solvent Dynamics. Nat. Commun..

[23] Bradforth S. E., Jungwirth P. (2002). Excited States
of Iodide Anions in Water: A Comparison
of the Electronic Structure in Clusters and in Bulk Solution. J. Phys. Chem. A.

[24] Villa A. M., Doglia S. M., De Gioia L., Bertini L., Natalello A. (2019). Anomalous
Intrinsic Fluorescence of HCl and NaOH Aqueous Solutions. J. Phys. Chem. Lett..

[25] Villa A. M., Doglia S. M., De Gioia L., Natalello A., Bertini L. (2022). Fluorescence of KCl Aqueous Solution:
A Possible Spectroscopic
Signature of Nucleation. J. Phys. Chem. B.

[26] Kumar A., Ahari D., Priyadarshi A., Ziauddin Ansari M., Swaminathan R. (2020). Weak Intrinsic Luminescence in Monomeric Proteins Arising
from Charge Recombination. J. Phys. Chem. B.

[27] Pansieri J., Josserand V., Lee S.-J., Rongier A., Imbert D., Sallanon M. M., Kövari E., Dane T. G., Vendrely C., Chaix-Pluchery O., Guidetti M., Vollaire J., Fertin A., Usson Y., Rannou P., Coll J.-L., Marquette C., Forge V. (2019). Ultraviolet–visible–near-Infrared Optical Properties
of Amyloid Fibrils Shed Light on Amyloidogenesis. Nat. Photonics.

[28] Stephens A. D., Qaisrani M. N., Ruggiero M. T., Díaz
Mirón G., Morzan U. N., González Lebrero M. C., Jones S. T. E., Poli E., Bond A. D., Woodhams P. J., Kleist E. M., Grisanti L., Gebauer R., Zeitler J. A., Credgington D., Hassanali A., Kaminski Schierle G.
S. (2021). Short Hydrogen Bonds
Enhance Nonaromatic Protein-Related Fluorescence. Proc. Natl. Acad. Sci. U. S. A..

[29] Mirón G. D., Semelak J. A., Grisanti L., Rodriguez A., Conti I., Stella M., Velusamy J., Seriani N., Došlić N., Rivalta I., Garavelli M., Estrin D. A., Kaminski Schierle G.
S., González
Lebrero M. C., Hassanali A., Morzan U. N. (2023). The Carbonyl-Lock
Mechanism Underlying Non-Aromatic Fluorescence in Biological Matter. Nat. Commun..

[30] Yang T.-Q., Hu X.-D., Shan B.-Q., Peng B., Zhou J.-F., Zhang K. (2021). Caged Structural Water Molecules
Emit Tunable Brighter Colors by
Topological Excitation. Nanoscale.

[31] Grasso R., Musumeci F., Gulino M., Scordino A. (2018). Exploring the Behaviour
of Water in Glycerol Solutions by Using Delayed Luminescence. PLoS One.

[32] Menter J. M. (2006). Temperature
Dependence of Collagen Fluorescence. Photochem.
Photobiol. Sci..

[33] Korepanov V., Yu C.-C., Hamaguchi H.-O. (2022). Hyper-Raman Spectral Signatures of
Structured and De-structured Hydrogen-bonded Water. J. Raman Spectrosc..

[34] Starr F. W., Nielsen J. K., Stanley H. E. (1999). Fast and
Slow Dynamics of Hydrogen
Bonds in Liquid Water. Phys. Rev. Lett..

[35] Wentworth M., Ruban A. V., Horton P. (2003). Thermodynamic
Investigation into
the Mechanism of the Chlorophyll Fluorescence Quenching in Isolated
Photosystem II Light-Harvesting Complexes. J.
Biol. Chem..

[36] Kühne T. D., Khaliullin R. Z. (2013). Electronic Signature of the Instantaneous Asymmetry
in the First Coordination Shell of Liquid Water. Nat. Commun..

[37] Titantah J. T., Karttunen M. (2013). Water Dynamics:
Relation between Hydrogen Bond Bifurcations,
Molecular Jumps, Local Density & Hydrophobicity. Sci. Rep..

[38] Chelli R., Procacci P., Cardini G., Califano S. (1999). Glycerol Condensed
Phases Part II.A Molecular Dynamics Study of the Conformational Structure
and Hydrogen Bonding. Phys. Chem. Chem. Phys..

[39] Chapter 1. Glycerol: Properties and Production. In Green Chemistry Series; RSC green chemistry series; Royal Society of Chemistry: Cambridge, 2008; pp 1–17.

[40] Shelton D. P. (2024). Correlated
Libration in Liquid Water. J. Chem. Phys..

[41] Goss L. M., Sharpe S. W., Blake T. A., Vaida V., Brault J. W. (1999). Direct
Absorption Spectroscopy of Water Clusters. J.
Phys. Chem. A.

[42] Tang F., Ohto T., Sun S., Rouxel J. R., Imoto S., Backus E. H. G., Mukamel S., Bonn M., Nagata Y. (2020). Molecular
Structure and Modeling of Water-Air and Ice-Air Interfaces Monitored
by Sum-Frequency Generation. Chem. Rev..

[43] Gao Y., Fang H., Ni K., Feng Y. (2022). Water Clusters and
Density Fluctuations in Liquid Water Based on Extended Hierarchical
Clustering Methods. Sci. Rep..

[44] Kazimirski J. K., Buch V. (2003). Search for Low Energy
Structures of Water Clusters (H_2_O)_
*n*
_, *n* = 20–22,
48, 123, and 293. J. Phys. Chem. A.

[45] Galvez
Vallejo J. L., Heredia J. D., Gordon M. S. (2021). Bonding Analysis
of Water Clusters Using Quasi-Atomic Orbitals. Phys. Chem. Chem. Phys..

[46] Tachikawa H., Yabushita A., Kawasaki M. (2011). Ab Initio Theoretical Calculations
of the Electronic Excitation Energies of Small Water Clusters. Phys. Chem. Chem. Phys..

[47] Turi L., Rossky P. J. (2012). Theoretical Studies
of Spectroscopy and Dynamics of
Hydrated Electrons. Chem. Rev..

[48] Roscioli J.
R., Hammer N. I., Johnson M. A. (2006). Infrared Spectroscopy of Water Cluster
Anions, (H2O)­n = 3–24-)­in the HOH Bending Region: Persistence
of the Double H-Bond Acceptor (AA) Water Molecule in the Excess Electron
Binding Site of the Class I Isomers. J. Phys.
Chem. A.

[49] Lee H. M., Suh S. B., Tarakeshwar P., Kim K. S. (2005). Origin of the Magic
Numbers of Water Clusters with an Excess Electron. J. Chem. Phys..

[50] Herbert J. M., Coons M. P. (2017). The Hydrated Electron. Annu.
Rev. Phys. Chem..

[51] Novelli F., Chen K., Buchmann A., Ockelmann T., Hoberg C., Head-Gordon T., Havenith M. (2023). The Birth and Evolution
of Solvated Electrons in the Water. Proc. Natl.
Acad. Sci. U. S. A..

[52] Lee H. M., Kim K. S. (2013). Dynamics and Structural
Changes of Small Water Clusters
on Ionization. J. Comput. Chem..

[53] Ziaei V., Bredow T. (2017). Qualitative Assessment
of Ultra-Fast Non-Grotthuss
Proton Dynamics in S1 Excited State of Liquid H_2_O from
Ab Initio Time-Dependent Density Functional Theory. Eur. Phys. J. B.

[54] Chipman D. M. (2016). Hemibonding
between Water Cation and Water. J. Phys. Chem.
A.

[55] Godinho S.S.M.C., do Couto P. C., Cabral B.J. C. (2004). Charge Separation
and Charge Transfer
to Solvent in NaCl–water Clusters. Chem.
Phys. Lett..

[56] Timko J., Bucher D., Kuyucak S. (2010). Dissociation of NaCl in Water from
Ab Initio Molecular Dynamics Simulations. J.
Chem. Phys..

[57] Bankura A., Carnevale V., Klein M. L. (2013). Hydration Structure of Salt Solutions
from Ab Initio Molecular Dynamics. J. Chem.
Phys..

[58] Siu C.-K., Fox-Beyer B. S., Beyer M. K., Bondybey V. E. (2006). Ab Initio Molecular
Dynamics Studies of Ionic Dissolution and Precipitation of Sodium
Chloride and Silver Chloride in Water Clusters, NaCl­(H2O)­n and AgCl­(H2O)­n,
N = 6, 10, and 14. Chemistry.

[59] Hou G.-L., Liu C.-W., Li R.-Z., Xu H.-G., Gao Y. Q., Zheng W.-J. (2017). Emergence of Solvent-Separated
Na – Cl^–^ Ion Pair in Salt Water: Photoelectron
Spectroscopy and Theoretical
Calculations. Journal of Physical Chemistry
Letters..

[60] Hassanali A. A., Cuny J., Verdolino V., Parrinello M. (2014). Aqueous Solutions:
State of the Art in Ab Initio Molecular Dynamics. Philos. Trans. A Math. Phys. Eng. Sci..

[61] Bouazizi S., Nasr S., Jaîdane N., Bellissent-Funel M.-C. (2006). Local Order
in Aqueous NaCl Solutions and Pure Water: X-Ray Scattering and Molecular
Dynamics Simulations Study. J. Phys. Chem. B.

[62] Mancinelli R., Botti A., Bruni F., Ricci M. A., Soper A. K. (2007). Hydration
of Sodium, Potassium, and Chloride Ions in Solution and the Concept
of Structure Maker/breaker. J. Phys. Chem. B.

[63] Carter-Fenk K., Johnson B. A., Herbert J. M., Schenter G. K., Mundy C. J. (2023). Birth of
the Hydrated Electron via Charge-Transfer-to-Solvent Excitation of
Aqueous Iodide. J. Phys. Chem. Lett..

[64] Dashnau J. L., Nucci N. V., Sharp K. A., Vanderkooi J. M. (2006). Hydrogen
Bonding and the Cryoprotective Properties of Glycerol/water Mixtures. J. Phys. Chem. B.

[65] Morita M., Matsumura F., Shikata T., Ogawa Y., Kondo N., Shiraga K. (2022). Hydrogen-Bond
Configurations of Hydration Water around
Glycerol Investigated by HOH Bending and OH Stretching Analysis. J. Phys. Chem. B.

[66] Daschakraborty S. (2018). How Do Glycerol
and Dimethyl Sulfoxide Affect Local Tetrahedral Structure of Water
around a Nonpolar Solute at Low Temperature? Importance of Preferential
Interaction. J. Chem. Phys..

[67] Egorov A. V., Lyubartsev A. P., Laaksonen A. (2011). Molecular Dynamics Simulation Study
of Glycerol-Water Liquid Mixtures. J. Phys.
Chem. B.

[68] Jahn D. A., Akinkunmi F. O., Giovambattista N. (2014). Effects of Temperature on the Properties
of Glycerol: A Computer Simulation Study of Five Different Force Fields. J. Phys. Chem. B.

[69] Jahn D. A., Wong J., Bachler J., Loerting T., Giovambattista N. (2016). Glass Polymorphism
in Glycerol-Water Mixtures: I. A Computer Simulation Study. Phys. Chem. Chem. Phys..

[70] Akinkunmi F. O., Jahn D. A., Giovambattista N. (2015). Effects of
Temperature on the Thermodynamic
and Dynamical Properties of Glycerol-Water Mixtures: A Computer Simulation
Study of Three Different Force Fields. J. Phys.
Chem. B.

[71] Lu W., Mackie C. J., Xu B., Head-Gordon M., Ahmed M. (2022). A Computational and Experimental View of Hydrogen Bonding in Glycerol
Water Clusters. J. Phys. Chem. A.

[72] Ansari N., Dandekar R., Caravati S., Sosso G. C., Hassanali A. (2018). High and Low
Density Patches in Simulated Liquid Water. J.
Chem. Phys..

[73] Schönfeldová T., Dupertuis N., Chen Y., Ansari N., Poli E., Wilkins D. M., Hassanali A., Roke S. (2022). Charge Gradients around
Dendritic Voids Cause Nanoscale Inhomogeneities in Liquid Water. J. Phys. Chem. Lett..

[74] Ansari N., Laio A., Hassanali A. (2019). Spontaneously
Forming Dendritic Voids
in Liquid Water Can Host Small Polymers. J.
Phys. Chem. Lett..

[75] Russo J., Tanaka H. (2014). Understanding Water’s
Anomalies with Locally
Favoured Structures. Nat. Commun..

[76] Russo J., Akahane K., Tanaka H. (2018). Water-like
Anomalies as a Function
of Tetrahedrality. Proc. Natl. Acad. Sci. U.
S. A..

[77] Offei-Danso A., Hassanali A., Rodriguez A. (2022). High-Dimensional Fluctuations in
Liquid Water: Combining Chemical Intuition with Unsupervised Learning. J. Chem. Theory Comput..

[78] Sosso G. C., Caravati S., Rotskoff G., Vaikuntanathan S., Hassanali A. (2017). On the Role of Nonspherical Cavities
in Short Length-Scale
Density Fluctuations in Water. J. Phys. Chem.
A.

