# Supplementary material for: Anomalous Water Fluorescence Induced by Solutes
Source: J Phys Chem Lett. 2025 Jun 30;16(27):6935–45. doi: 10.1021/acs.jpclett.5c01189 (PMC12257589; doi:10.1021/acs.jpclett.5c01189)
Supplement: Supplementary file 1 [file jz5c01189_si_001.pdf]

Supporting Information for Anomalous water fluorescence induced by solutes. Cartesian coordinates.

Anna Maria Villa, Jessica Di Paola, Diletta Ami, Luca De Gioia, Antonino Natalello, Luca Bertini
Department of Biotechnologies and Biosciences, University of Milano-Bicocca, Piazza della Scienza 2, 20126 Milan, Italy.
Luca Bertini
Email: luca.bertini@unimib.it

Cartesian coordinates in atomic units of the most stable forms of the $(H2O)_{110}$ cluster (structure 1 in Table S3 in SI)

```
$coord
  -13.73127784229610    -4.67161200184525    -9.87671248729932  o
   -2.63569904517042   -12.42116283178789    -9.64407876416082  o
   -4.65606672148709    -2.07622402801332    -9.32882138453116  o
   -4.67985835475437    -8.05978837423178    -2.42212762690890  o
  -15.73202502184697   -15.16605536842551    -1.36629775847512  o
   -8.47822558224133    -4.68079312592638    -3.25322861929747  o
  -16.98569922980458    -7.16144626200077    -6.08002660536378  o
    0.61780197200960   -10.15621338018793    -6.18659287032693  o
   -5.96275980853159   -11.05092431443577     1.66138015479661  o
  -15.00893062462623     0.53270742253077    -4.33598512084722  o
   -8.82253219221362    -5.13808057661656    -8.47604444902906  o
   -1.27359651394042   -19.00937331654730     6.13849622143765  o
   -9.82909798500769     9.91326480356911     8.54852099532245  o
    5.51540536146280    -8.57848162442345     2.37806747285445  o
    1.09250247454625   -10.45361377980126   -12.93065103226057  o
  -13.76114071913072   -11.22907690237814    -4.05393670484838  o
  -10.74441943803860     3.73609731982820   -10.52237458918436  o
   -9.45992329194300   -13.54097560694051    -6.80620372049432  o
   -6.25811962799561    -9.46351576030026    -7.52241593069444  o
    6.54539145822735     7.47336954246185     3.62724776515391  o
  -12.20982977668823    -7.37343629252599    -0.97598917070759  o
    0.20533616732820    -7.37283377960036     0.53467403240458  o
  -15.07845559137891    -6.13419686703184     8.63024193220845  o
    0.22922949230784   -19.10741961012965     1.34529417526514  o
   -4.04065477660462    -1.50495635338938     5.95625853448960  o
    5.00026690961906    -8.18515226629799    14.29684517599290  o
  -10.32638739826626     8.72159663241353    -8.41343078370021  o
   -1.96357581941520    -8.13665390793166     9.22145417803695  o
  -13.16430276457931    -1.81561706822848     5.90067375701179  o
   -8.54630274924803    -3.19013574936296     3.87855368724462  o
  -17.29505188496771    -1.59127617879195     2.45449722180306  o
   -5.31082677115177   -16.45576796005328    -6.93760045531712  o
   -9.74254333054776    -8.15719638501748     3.53621281424893  o
   -6.38244041045938    -0.96150188240362    10.44917602459775  o
   -6.88571690159937     6.16523630473138    14.10301486445166  o
  -11.54207269413062    -9.85518584984567     8.12500905933479  o
   -7.42247975481327    -0.43453077554044    -0.24310539818023  o
  -19.58295836551281    -4.96292839020143     5.91174135288699  o
   -6.57684356241949     2.35167658838116    -7.43263304826315  o
   -1.89275395582169    -0.96233292788611   -13.72029645266015  o
  -14.67502473540922     0.68228800036070    -9.62502806411788  o
  -17.28465490833017     3.35536739220545    -0.18236303283292  o
    5.00992153405316    -9.97068858634001    -9.09552375446944  o
    7.54714001244215     1.86153066626891    -3.54923750736677  o
  -10.73264379725955     7.44248184780465    -3.43394940572675  o
   -0.80580858077173     7.80621443164947   -12.40557734410500  o
    0.62165183303797     4.17002154851878    11.84821923881161  o
  -14.74922315753648     7.98227307561974     0.32428246577650  o
  -11.90825412449246    10.49826271050975     3.59799044442839  o
    0.18996054546256     3.36371922014530   -14.99333982448594  o
```

-2.98159960628173	3.36287573212675	4.83862661445516	o
-5.08992796517267	9.66145163848815	10.32779247796952	o
-2.60096070489263	-0.26627206762529	-1.48119983068102	o
1.57855313021237	6.50138816101041	-7.64169423520683	o
1.32791654803957	-3.65091431407028	-2.95151511198688	o
0.75922689772019	11.50305209752491	0.09181219679048	o
-10.22739997731211	2.29292914392320	-3.63936106981513	o
-0.45081758083718	3.97984389783448	0.48097903968856	o
-7.89803424083092	3.75694142510799	3.57068333640129	o
4.70262305444413	3.61646262105719	0.54663475231581	o
2.01128643577047	9.98817568084436	4.67676978119920	o
-5.02084836918581	9.66338684899129	-9.32816270387081	o
9.52847221613671	-6.98001347592976	11.09678186926020	o
-11.17407065161579	5.68797031148017	11.61070433123395	o
-1.10568135501945	6.94539979911952	7.94815282908072	o
-3.32295383985729	6.23377535876220	-5.93805156021094	o
6.75646374812190	0.08912061733771	3.93659561267354	o
-10.35640958375889	1.91257141983703	8.09632930297214	o
-2.23417611856234	-2.92075980041939	16.30583648412483	o
4.07040875831297	0.03230914650742	11.48837030658175	o
-3.03232058107213	8.23431395619155	-1.11877355935348	o
-16.10226642821846	-4.09413302268858	-1.99886759759097	o
-7.84463472469924	8.35789273722776	0.96377401889769	o
-7.31916298122657	-16.07549658725154	1.37443358936563	o
5.39124582916735	-1.55642888267449	-11.22356763995862	o
-5.91758265426753	-16.33433180312323	6.35772557566174	o
10.49656623423988	9.83124633759819	1.01304924837069	o
6.46099206769667	-3.28813163628660	-3.82452306154032	o
7.64379436735098	-5.31541526539840	-8.46998007308977	o
3.57121845199009	-14.99538343635548	1.71807491915641	o
9.20653170199605	1.66898269555553	11.17916101202935	o
11.09054028555500	-10.78001056737262	-2.79524799506891	o
12.85296427187071	5.18838509826431	2.41929403609891	o
0.16497099709629	-12.28530536741915	-1.30007239696132	o
8.46589470782402	-14.83900217196197	-0.28389103692784	o
-3.22033049269902	-16.44573254292774	-1.92715896485906	o
-0.82034337391034	-5.11012212350675	-7.37355544033827	o
1.34848357063881	-4.89224864682681	-12.05602706866163	o
11.81359222899663	-2.48180311247015	10.11292650279898	o
-5.07624892184548	-5.51774478915796	12.67804904995502	o
10.94602127164450	-2.14206915351124	2.10834727234165	o
9.18303102868404	-6.30862161575194	-0.56637206783122	o
-3.10374700026481	-11.95042511093487	5.92176987192250	o
9.29408053798011	3.94771987504690	6.31138634164005	o
5.59977425222530	3.10563005562222	-8.24193462756736	o
5.87562558396537	-7.52449260150824	7.34096030650708	o
-11.24977963656277	-14.95668289100668	7.13756438722495	o
12.38283599378451	1.89305052330429	-1.32929410589666	o
-12.50188455470472	-17.00700915569202	2.14656572642379	o
-3.76768257614893	1.82968709775638	14.00247738063516	o
10.17803456942575	-10.07729277829828	-7.70704071631519	o
5.57480089281930	2.85655257436635	-14.04807555509471	o
-0.53707694661813	-5.12859883480241	5.15121468774643	o
2.70470672783135	-10.29821619737880	10.11476803161166	o
-8.99447124446089	-8.98875889573751	12.58261698163011	o
3.83321513106128	-2.76231853971892	7.04586085996314	o
1.51168988060398	-14.32567185992784	6.52165707697094	o
2.61013930977068	-3.34037036172244	15.26798473379959	o
-14.13477741026592	-2.84940259585520	-9.89957665131586	h
-14.90961800357227	-5.46668326330423	-8.67737821778245	h
-3.29720075365011	-14.06816655658237	-9.06754638396630	h
-4.02399543196537	-11.21235563015000	-9.20404460484710	h
-6.22555146042431	-3.10383024901606	-9.40658637593173	h
-5.19913562139416	-0.46920564901398	-8.49359938095881	h
-3.00365013054180	-7.45562498963592	-1.89699625409664	h
-5.15663684984765	-9.25703496002670	-1.04196730364807	h
-16.34147135118787	-16.35313702406811	-2.62956894942412	h

-15.09925516687913	-13.67273013545666	-2.34991580108889	h
-6.94682628281347	-5.72374860652812	-2.88657923153678	h
-8.18340836986741	-3.02989058849619	-2.40872112802268	h
-17.04440861370063	-6.03933763568410	-4.56021010983889	h
-18.73730227607091	-7.43534039812642	-6.56844810333050	h
-0.06076873916048	-8.42248285923968	-6.43808015917765	h
-0.46576889973561	-11.20199784817295	-7.31515627307660	h
-4.70309155245116	-11.23700715424715	3.05591168623366	h
-6.58171820265965	-12.80481362879798	1.34681687430422	h
-15.20421841022099	0.56149474360745	-6.19949176068172	h
-15.30292162323264	-1.22601950421190	-3.74691755966103	h
-10.60086342789722	-5.05463379146692	-9.13903742046217	h
-8.94208416334212	-4.71601049046247	-6.65202946307013	h
-0.81619148704996	-20.61362131972012	6.91160301146923	h
-0.81678504622710	-19.17524551417501	4.27476348421585	h
-10.68065835417728	8.55097506159519	9.49198453333415	h
-10.57149320604112	9.94419602948114	6.83653785593520	h
3.89911949496598	-8.02240161612266	1.66062547941794	h
6.84788756433729	-7.75363762729314	1.33801761992394	h
1.04073348812276	-11.17507810011861	-14.62078887007842	h
-0.31099107664936	-11.27158996966234	-11.98679546183756	h
-14.94028374035734	-10.19018182268872	-5.05900957018165	h
-13.04742799227024	-9.96561254026225	-2.83605433752196	h
-9.12847398462442	3.17580022557831	-9.75595476566075	h
-10.88467362072489	5.51011728830314	-9.96014066466472	h
-8.53753962189374	-11.93736997379728	-7.11949226130013	h
-10.95657579212456	-13.04819205145340	-5.81821590619050	h
-7.19651777530958	-7.92965845109405	-8.13187366938930	h
-5.54950133379649	-9.00208637694263	-5.85650320341155	h
7.85624303492315	8.55294943845423	2.83612370729747	h
7.45617353875580	6.42812109002773	4.90254492049448	h
-11.60124254042687	-7.73264673019913	0.77034717516885	h
-10.81783141552276	-6.37845435291025	-1.79243412727507	h
0.02010668938542	-9.23199873039785	0.40148088896597	h
-0.24125643991911	-6.75297297726674	2.26682393701473	h
-15.30999158801111	-6.01589023110217	10.45224997069799	h
-13.94901418651102	-7.64149766329051	8.38308722642425	h
-0.95336546863719	-18.49748284520149	0.03676055588483	h
1.64334784649527	-17.87239798962007	1.32148629305633	h
-3.55499285915878	0.26765418089637	5.47897125927912	h
-5.64504618150686	-1.96455730122103	5.09296492422298	h
5.26685473862037	-9.27908117876209	15.74997294687849	h
4.15049147443456	-9.25062456275534	12.99347415457765	h
-11.51171843620264	10.09307437176521	-8.72224392531559	h
-10.40387937944940	8.40005108430959	-6.53886913750360	h
-0.34750821738161	-8.79637677086824	9.93256593709274	h
-2.93651585580337	-7.23142934385477	10.55007841887127	h
-13.70569063921664	-3.16237900977257	7.07654549978480	h
-12.51388253375130	-0.37269593283095	6.92606621218607	h
-8.73845415230153	-5.06807680993934	3.74550054777615	h
-10.20538212106542	-2.63056456081604	4.58534524019730	h
-18.44605876551246	-2.58250080405296	3.55010233359686	h
-15.74587025445367	-1.46166646608005	3.50127494311449	h
-6.98697124956994	-15.56461545793489	-6.73984369064461	h
-5.70826406975576	-18.07199988690093	-7.72093651767524	h
-8.36289486642363	-9.31866376367694	2.96493978979033	h
-10.34068456289536	-8.78164362637619	5.21348483272726	h
-7.92505713095723	-0.08524421753866	9.84515762929900	h
-5.33789613289412	-1.15315773279669	8.87733290202244	h
-5.76412928386677	4.68420118260339	14.34101721271201	h
-5.94874608734798	7.37824344588128	13.03858540408160	h
-10.48119991330514	-9.58418164819412	9.66369840848119	h
-11.61373986671788	-11.72840033596010	7.85377455784732	h
-7.78204818014788	-1.55413486196853	1.24000872872665	h
-5.54062506848676	-0.46264054681043	-0.62044607931229	h
-20.50524859008098	-6.45100129106138	5.35462821842823	h
-18.10335700171092	-5.60896543211843	6.85289922201963	h

-7.82924688105721	2.17872212950183	-6.04371869624319	h
-5.35160171256199	3.65430881650664	-6.83290644593978	h
-0.71695903736906	-2.39361152226624	-13.47405530715869	h
-3.10416501463872	-1.16362314782204	-12.30007387117073	h
-15.99442549578593	1.28218363018943	-10.75648503511851	h
-13.16185083493528	1.82184470569102	-9.96357685268221	h
-16.70247555453369	2.57435631253482	-1.76771330488594	h
-17.56093033608265	1.91652615003747	0.95883715426587	h
3.63266287395999	-10.10912534659081	-7.82060744758939	h
4.12608861684492	-10.31845672391219	-10.69406791442236	h
7.13627884472103	2.51749839186940	-5.27142081760837	h
9.36358351694559	2.10143499086728	-3.16410723582737	h
-10.56972965044128	5.55913443640538	-3.46882755670566	h
-9.53491576480695	7.95783559358309	-2.08261341511938	h
-0.06699150443074	9.13049790271205	-13.44660824701911	h
-0.59319860342292	6.20839309329250	-13.39068347360821	h
1.88634274398489	2.80791407613263	11.63501179353641	h
-0.84257882775720	3.35315181004874	12.67188796888179	h
-15.70216348088557	6.36548670231351	0.41509499500292	h
-13.64902757304116	7.81916041682081	-1.17119778499689	h
-13.11545293044142	9.42478208885452	2.55376341889843	h
-12.58098036989166	12.20718596296219	3.50492965084008	h
-0.63572087070278	1.72788947231729	-14.36418264237791	h
-0.18700228115026	3.39733113869487	-16.79431321195771	h
-2.33536630503595	4.63943464426101	6.09828179430351	h
-1.88719323256410	3.57136506411730	3.30283186342720	h
-6.77514423707497	9.83632958037692	9.44893906696548	h
-4.64568857288220	11.35604085232073	10.88735906284442	h
-1.74398221457345	1.30378668755239	-0.90660985338162	h
-1.23885274218575	-1.42874906327391	-1.99934559184475	h
-0.10145394702857	6.11906623387366	-6.85710880575531	h
1.07369535503031	7.17375974254805	-9.30943685465497	h
1.14926505522426	-5.04292949373213	-1.71160523919920	h
3.18133933082636	-3.39236779887277	-3.19994735210306	h
1.20201752472248	10.92272015631060	1.88067474605642	h
0.63282287365563	13.33541910415827	0.16889951281062	h
-9.40161131502899	1.36492489324470	-2.23755415127272	h
-11.95389471118279	1.54580060565077	-3.85607033085514	h
-1.16434400722056	5.59620695636013	-0.16156157733264	h
1.43494525208636	4.01398918265436	0.39766058032962	h
-6.06020513241977	3.74075416983929	4.07867412811422	h
-7.99028382313350	2.48302133517288	2.21336192423619	h
5.66665177754633	3.27230562716978	-1.02988724388898	h
5.45053875938204	5.12677877051503	1.38940858485167	h
3.64989434223634	9.09205356944670	4.54346844539279	h
0.93268214913201	8.95577385103035	5.80634725181981	h
-6.85187337419159	9.35386840703644	-9.46760846471214	h
-4.16615191509609	8.98766494341086	-10.82889390393748	h
8.50571016606752	-7.37503842180188	9.57670364110098	h
8.31517361353901	-7.19551312240296	12.48369290685866	h
-9.57405736807150	5.76382762741021	12.69115907102183	h
-12.59619912013247	5.70818436390049	12.77470227957583	h
-0.30629481913677	5.98220263449970	9.39086633187139	h
-2.46563192261137	7.97090976065941	8.72334438739651	h
-3.34114584242278	6.94597574834503	-4.19804816329870	h
-3.95950434322895	7.62981546899206	-7.05755909511828	h
5.90323753328738	1.09921014535816	2.60060316948715	h
8.21909367075847	-0.83562572238618	3.13568560285363	h
-9.60453437994001	2.72878115462374	6.58099693975696	h
-10.82043683828206	3.29074666123951	9.29134807261305	h
-0.35656148235018	-3.12220969105586	15.89274982140784	h
-2.36676052603351	-3.15662353359504	18.12453624696047	h
3.86810706762187	-0.92807345293496	9.87970018769791	h
5.84536330212609	0.63296805589704	11.48501994317851	h
-4.65688840970394	8.52922229809507	-0.22632824256767	h
-1.89415679577651	9.68779536086581	-0.75318660313542	h
-16.77034628083944	-3.28911361622532	-0.42478426782995	h

-14.69768550956718	-5.20805804606626	-1.41050291047536	h
-7.97150480992365	6.74121244403280	1.92639966406043	h
-8.96940347870610	9.52393634584047	1.90168296036849	h
-9.14175148812549	-16.49663101652695	1.31158507830710	h
-6.80443048406411	-16.40639181726953	3.16589239105244	h
5.65593898206000	-0.30127927383430	-12.59743970287866	h
3.96731195953453	-2.65311064213375	-11.75120588431658	h
-4.54047943686045	-17.58692496683023	6.55550772061344	h
-5.02232026524273	-14.67817940683744	6.47655857745360	h
11.19518026924279	11.48527369725984	1.40615521826911	h
9.92052314177181	9.91644380880877	-0.81354975119917	h
6.95765749383326	-1.47053467653857	-3.74245327650353	h
6.87260699294373	-3.90865856278569	-5.56418357105003	h
7.24733663803966	-3.93340110317778	-9.68307991668862	h
6.42772782309997	-6.68352736597832	-8.86250703144615	h
2.63412441617012	-13.83687321091576	0.57855622442985	h
5.37016832707172	-14.84991496222437	1.19397042328165	h
9.92348760332525	2.40320330040257	12.70514657625734	h
10.23058114454065	0.08242750869277	10.82783737509351	h
12.90217396588701	-11.04972045445980	-2.62148242856302	h
10.76767649513247	-10.60171868589478	-4.67616940147587	h
11.89792460074350	4.69459264460873	3.95095720988278	h
12.23689016913660	6.89116191781434	1.96913978722124	h
-1.17342135369094	-13.58634995304866	-1.49558996935379	h
0.44468473155563	-11.58914548277821	-3.02167137138710	h
8.74397397861929	-16.18137547048782	-1.50742006892045	h
9.25053204146942	-13.32353172447860	-1.04626565983943	h
-4.78992116550947	-16.44944954285477	-0.90124531763357	h
-3.79517193889675	-16.61771651694738	-3.69884171702425	h
-2.31618078074583	-4.04570027905903	-7.81618045873493	h
-0.02826274294373	-4.34258480699657	-5.83800474410048	h
1.32341575016804	-6.64804120840306	-12.66451004611444	h
0.67615116722987	-4.98800534984930	-10.28764329456750	h
10.99956595257894	-4.13591074380588	10.57298580370617	h
12.25570252023431	-2.62016701169105	8.33165424300698	h
-5.79324262819610	-3.98024416366828	11.85734795000184	h
-4.13788154952078	-4.82834745208609	14.15657816779364	h
10.60837614103345	-3.75848109284971	1.22309630410387	h
11.65797739324058	-0.98936626575327	0.82827740574439	h
10.00069872678804	-7.75776902246697	-1.42311706824814	h
8.26025705217677	-5.29868269775749	-1.86659570786084	h
-3.02546698193516	-10.56085344731851	7.19434182580635	h
-1.36591811313888	-12.69759745258089	5.94493397062993	h
8.38002715731835	2.46345315897532	5.57495990413960	h
9.42003801943517	3.54001624495302	8.13196600991698	h
5.09621521297770	1.43155221173916	-8.89089996428595	h
4.02952100009637	4.10705045951658	-7.96963317072383	h
5.26297378865475	-5.73649743140249	7.32365825470583	h
5.87578502754366	-8.02577413672986	5.50575055104120	h
-9.50673831566895	-15.60449310736195	7.20100342208834	h
-11.89943551075372	-15.51228132976478	5.48440632437561	h
12.58629825519171	3.19681671588147	0.07248746520878	h
14.01491663564909	1.81489675462601	-2.17242129173252	h
-13.72624498701072	-16.29309371910781	0.87513278372347	h
-13.02496588941123	-18.75181679745060	2.40221497207241	h
-3.23470095199154	0.47785653764174	15.17148355411192	h
-4.69617849402958	0.89812090542638	12.65300224829318	h
10.11838541933663	-8.25383280940575	-8.06924501030424	h
8.47236571662220	-10.62171759549307	-8.23445316382828	h
5.88744448985706	3.70290474701023	-12.42998182375301	h
3.76912161826253	3.15656973880316	-14.41567628274201	h
-1.82692179566710	-3.74718681393814	5.22919169661696	h
-0.99521250851969	-6.22212417079788	6.61420470829111	h
2.51183746806267	-11.94638407472876	9.26174285694151	h
3.87568754415851	-9.30030655779544	8.96795025062207	h
-8.48426417089066	-10.47090159441061	13.54066342761860	h
-7.57387458038591	-7.76058275362105	12.75187598648137	h

```
  4.78636124812513   -1.74842702076934    5.76274410430073  h
  2.24668195941834   -3.36832370355957    6.24281302015185  h
  0.83728498729970  -16.05245351640691    6.74505925284318  h
  2.53148806471650  -14.41431947741022    4.93466913162644  h
  3.24730854511824   -2.12742978905954   13.97575452848185  h
  3.40838199377204   -4.98454447167060   14.91112062090122  h
  8.85102808060597   10.09944767996861   -3.70981424257885  o
  9.50155485038137    8.96932589040537   -5.00221139971989  h
  6.95824057691869   10.13585675475042   -3.94797989844995  h
  3.83220059909543   10.22083986886136   -4.31829864570955  o
  2.76189253682438   10.51323225041537   -2.82417818180326  h
  2.97284717466465    8.97844544806116   -5.41772316775248  h
$user-defined bonds
$end
```

Cartesian coordinates in atomic units of the most stable forms of the $(NaCl)_2(H2O)_{108}$ cluster (structure 1 in Table S4 in SI)

```
$coord
 -1.02010630540908    7.27569997771130    1.33056379652566  na
  7.56010070674500    3.40954978620374   -0.42182358935599  cl
  3.58916515354588   -2.25095813351870    6.37865175537623  na
 -2.49409030557376   -6.02717187587146   -6.95981535221756  cl
 15.20196362061719    0.46622239989788    2.65494469790774  o
 13.50855187982512   -0.29927295410944    2.38225163051299  h
 16.23014044951855   -0.92840297576147    3.35520124116282  h
  8.21657825126865    1.41679614778904  -13.54556394567407  o
  7.59442127595965    3.30192684765278  -13.46161876350548  h
  9.10352570332636    1.27846624731254  -15.15129749288163  h
  0.93252607103106   -0.61741292622149   -1.50509950269552  o
  1.42661095205302    0.17435950819517   -3.14622380890791  h
  1.17068481083581   -2.45941523374848   -1.78955541450406  h
 -7.55017548302657   -2.48724481183468  -13.73700658234485  o
 -7.31585808674727   -3.70769815440609  -15.09283138199551  h
 -7.76225303694420   -3.50051356226455  -12.13330833066852  h
  4.31188161156490   14.81143487756279   -5.02663153258681  o
  2.67696237354819   14.50756119363526   -5.86789942707517  h
  4.24873009351300   13.92232097959399   -3.37871622187149  h
 -3.00649523002506   -5.94724294045898    3.43748678223520  o
 -1.19724805698007   -6.17160835842434    3.97908831541093  h
 -3.65637642573814   -4.63958766164878    4.63459353489477  h
 11.82591161734028   11.37605583400939   -3.40821482221812  o
 11.86822451637117    9.55975026833640   -3.86313753861317  h
 10.78311194803662   12.17883725697777   -4.74122998210019  h
 -7.96536176839872   -4.94531483470415   -9.28841435364468  o
 -8.86804620696859   -6.60321653592608   -9.19567552014282  h
 -6.25905801918793   -5.23732504371495   -8.54972226873923  h
-10.37338497627259   -8.10670846837186    5.78610032439747  o
-11.74997010632240   -9.17838587045136    6.37208626653466  h
-11.04717892031059   -7.19819102138917    4.24677660629071  h
  7.72719317481972   -6.76368437021626  -12.78689679793595  o
  7.34040370210609   -7.79135630891368  -11.21973192862189  h
  7.71864426200849   -7.94734559874434  -14.19396168592086  h
 -1.67773877301095  -11.76726675542026   -8.03338646056523  o
 -2.21107199876682  -12.15428180729915   -9.75074672638281  h
 -1.96564640781748   -9.91460994157127   -7.81454473124332  h
-16.01114525540491    0.62037590248181   -0.39247019747541  o
-15.95748016200945   -0.73160864154017   -1.73293563062521  h
-17.35695739320957    1.77089511489360   -0.88846419355881  h
 -6.87506544136560  -10.19942669739913   -1.39556583763729  o
 -6.21871056138893   -8.44268549645992   -1.63014419092922  h
 -6.67340173784917  -10.46851817464841    0.47659769978201  h
-11.04820155204760  -11.23253345044663   -3.89326691937990  o
-11.09490394759843  -13.07307120537055   -3.93251701176780  h
 -9.47057616397821  -10.79945185287628   -2.85677461855774  h
```

-12.26425083300160	-5.57368704633227	1.78460773724753	o
-11.15884044702319	-4.43949243641355	0.74209868109917	h
-13.23590718843407	-6.58092174385532	0.54272770180092	h
-4.54819627108396	4.73857515808047	-4.35852607659988	o
-4.97729564010386	4.04037783105700	-6.04995754616765	h
-6.13620860893558	5.48103389016245	-3.65967482688804	h
2.23021262899144	1.38962045145427	-6.20859035076529	o
1.53735329076289	0.38305667730499	-7.64590690641307	h
1.60709024906915	3.15910844900098	-6.39869474534142	h
-10.67537707639968	-9.34552195564170	-8.87249145339009	o
-10.73442808925773	-10.04975199102238	-7.13408417900129	h
-12.42971317271897	-9.22549972862630	-9.40819815655760	h
-2.72682384955719	2.86287861037648	0.07465086831356	o
-3.53266576321045	3.28897295799053	-1.57352547639950	h
-1.58555765898066	1.44014071599501	-0.35445590463654	h
-9.30231823166229	-2.42619709547224	-1.14884584037711	o
-7.59271674128712	-3.21238582461120	-1.20194280944366	h
-9.80123417479052	-2.17937900553885	-2.94892605786125	h
-4.94903223059732	-5.33854574730852	-1.34863747737912	o
-3.82281295912489	-5.15609792126464	-2.82149896896072	h
-3.90803414886701	-5.37235707945365	0.23076659025093	h
-11.02088355290631	2.33824456139450	0.47215722434299	o
-12.82993787156326	1.97578404971556	0.13489117450795	h
-10.14276034996364	0.73809056108289	0.03627727734570	h
-5.85753624258673	-10.32486223825793	3.64122522337141	o
-4.72349726536040	-8.81095172840098	3.72856039563063	h
-7.36353664024474	-9.83937463659061	4.62373645685428	h
-5.72251531268823	14.13572559570915	-0.59155790570794	o
-7.37374868999401	14.82517186161777	-0.16551086979555	h
-4.50626778765287	14.84222006129219	0.74185733197471	h
-15.59300841193804	-2.13815442017620	4.37741044246221	o
-16.05664587600263	-0.98839324561072	2.99207027035884	h
-14.52377624496634	-3.40671943831077	3.52837887111594	h
-14.87355847298867	-8.21738313801843	-2.04234054024155	o
-16.40106018579093	-9.16304557861043	-1.65020872442615	h
-13.63557490373864	-9.45675876184709	-2.74629988870387	h
9.17423941030066	-16.94409715495291	4.04856527353981	o
8.06487512685014	-16.40771924560379	5.52495129953457	h
9.00318635796728	-18.76850833421097	3.93721582855306	h
0.05973947099170	4.62055810832122	5.29647052568420	o
1.05605043010822	3.30343173442145	4.38878910639657	h
-1.67153694641434	3.91677848199774	5.48991717250473	h
-10.56389504593442	8.00522007441136	7.72887924245148	o
-10.85655813450321	7.63903923215781	9.50556451083751	h
-10.44317959739724	6.31656766066967	6.89665608151212	h
7.61423972823556	-10.58221556112895	9.99587259018066	o
8.29588645923115	-10.38372917493977	11.69199435442712	h
8.68442547485916	-9.48754782213885	8.86229646071384	h
-12.41249981878100	-0.10592584720575	8.08403531492682	o
-13.70136177359648	-0.78488029541871	6.89251283394404	h
-11.39255878416667	-1.56698518757127	8.60419063028665	h
-4.42941073888670	-2.41153184554693	6.97632649926800	o
-6.03853858856614	-2.86202954192420	7.80989868798440	h
-4.63239862228287	-0.69324101558615	6.19408612713081	h
-0.77873900724317	-16.15742099599921	-0.53895439157734	o
-2.01662655146947	-15.47185645353736	-1.82025735464584	h
-0.88785059376596	-17.98923775268885	-0.66127525087969	h
-9.75522471912382	3.36607208018137	5.49672561996589	o
-10.68471229360334	2.05126200165671	6.52388163811730	h
-10.37000362920041	3.11245210679871	3.74082773878236	h
-5.98124927085911	9.80627224885206	6.00561741448965	o
-7.61371794262293	9.30065504875594	6.78582983362914	h
-5.52197748220015	11.46408840802173	6.71921693050035	h
0.61847574477401	-1.69497452772968	-10.09316301838627	o
-0.00524716123785	-3.26030233826239	-9.29999810546786	h
2.12904841592509	-2.11221968023305	-11.20943847966802	h
-0.30159955597285	14.23999282498565	-7.91809601327265	o

-0.27161134207448	15.71231162818986	-9.02066551734294	h
-2.00621609365214	14.29992405426952	-7.04909223199723	h
-10.90565839534422	-1.77137055768623	-6.14381734043740	o
-10.88617719680979	-0.07097699594061	-6.96250813169643	h
-9.87971858035592	-2.87347386997736	-7.26291140625669	h
-10.65679824747321	2.83151231439401	-8.73262628194367	o
-11.21301752509720	2.62081060720620	-10.49091912398937	h
-8.78041177080116	3.10257500060324	-8.93140975193949	h
-4.89596232231479	14.34607922772650	-5.84967384305296	o
-5.11153132905404	14.34131059896712	-3.98974075576736	h
-6.17220395208714	13.18621928763603	-6.54807247762942	h
-3.21424950563216	0.61341680771398	-12.50960181736776	o
-4.44399775229049	-0.65884617996201	-13.07955542041578	h
-1.76247353026877	-0.31793809783480	-11.70462789622830	h
-10.44673695163392	1.88203395715931	-14.30099668833283	o
-11.93151285804962	1.45889882872792	-15.30157499110424	h
-9.45221957624420	0.28405705291943	-14.18231294561558	h
-8.74122516838071	6.49804136632426	-1.86491916714564	o
-7.72434350138056	7.47396380815847	-0.59926703184501	h
-9.57093788797757	5.07539183946503	-0.96358347353842	h
-11.37882238422153	7.76036287346335	-6.22001545761399	o
-11.38929762461066	6.10758603771979	-7.08262358193675	h
-10.60397065665054	7.44311827667454	-4.53826350049224	h
0.67802452952453	11.36158307126280	2.82079446340024	o
2.19043290965118	11.78023394270436	1.77610611587654	h
1.33165735604071	10.87988357405447	4.51941523418167	h
4.52108783502920	-2.44452531217268	-13.13186646604916	o
5.58386015137314	-3.97494671928390	-13.04947433878381	h
5.71084758690099	-1.00516292052606	-13.30980854461448	h
-8.70497138152768	11.32741478090638	-8.47197896591371	o
-9.78370376162226	9.98683834373411	-7.59968527970024	h
-9.88209437229619	12.57840883577437	-9.12818582115452	h
-1.94724571167944	4.34531308163730	-16.24195621146437	o
-3.63570829708447	5.09357687975391	-16.34200466677594	h
-2.16713199242281	2.92530772845294	-15.05505087702741	h
7.75695372665092	8.03884613090265	-8.60662772365231	o
8.89253557312278	7.24911284425730	-7.36129826082855	h
7.97816480680403	9.87120025563067	-8.36100303910899	h
-15.61207954857147	-2.98633007463313	-4.02592028823619	o
-14.03865220694573	-2.64669132013989	-4.98719877509848	h
-15.48896600227347	-4.75722734049648	-3.48740134829630	h
-4.68669561353073	2.25381289248081	4.85770564172249	o
-4.37934962557275	2.33531280694889	3.00009973795091	h
-6.45063373244279	2.86691708228565	5.16514559345185	h
2.51028244939540	-9.31128530623477	9.24859131188714	o
1.86841504444435	-8.42308926739235	10.77928617333937	h
4.31716940614740	-9.67681396757745	9.59849734730538	h
3.07752323355024	0.85033534866922	2.96862214411606	o
2.35267053045152	0.27240403882990	1.31626082094636	h
4.65137150917383	1.68970791707329	2.42673395325710	h
10.46868224366210	1.08923285156432	9.47153078494468	o
9.74321021329883	-0.28712273569155	8.43987685919244	h
11.62857371509744	2.08271997720528	8.34286827738758	h
6.79412511716681	8.24588350155140	8.25046890638950	o
7.02627915784400	6.82521955236674	9.44260417032098	h
7.85973582925390	7.86453158942026	6.75634805389526	h
-0.19409496315302	12.75628577752981	10.56223090084671	o
0.56714380135609	11.35047911719678	9.54779543525443	h
1.21555661006926	13.86306156851056	10.96970415548676	h
8.80433823948115	-13.87368307902509	0.09061841438653	o
8.94165130453960	-15.12563560607438	1.53133216748918	h
7.04394510955059	-14.01323232606586	-0.56006796668909	h
11.14956812185958	6.26544798734569	-4.67789076686740	o
12.45105896544824	5.03299338108648	-5.23004420148831	h
10.20086389436242	5.43126399343381	-3.31258735469815	h
-2.58686811561145	-14.80858192953666	4.48908466569449	o
-1.97118301621667	-15.17250633827755	2.77160954612920	h

-3.74339890954896	-13.36709870395320	4.32211886941943	h
-5.30160171223658	9.05069199267254	1.06064649168157	o
-5.66691448419612	9.33145736146446	2.91774279555175	h
-5.40363461181791	10.77822115251300	0.33645089927546	h
13.44666312866494	3.95349812969450	6.38819274688939	o
12.46473161013358	5.25910645015885	5.49638598150783	h
14.28280967860185	2.93589802946113	5.06220640891431	h
4.59245802091930	11.89761115148765	-0.55617770120873	o
4.20312661459871	10.13664966147574	-1.08562198024147	h
6.36302625967045	11.89682907580780	0.07381723045579	h
-2.50857540877100	15.53761145775789	2.92736572513348	o
-3.17844870441926	15.51615079622462	4.66659646837817	h
-1.25361647522739	14.13179486704376	2.90805009203217	h
-4.19205936057238	14.53441999475157	7.97626767861219	o
-5.23814200007677	15.55798377196456	9.08725482284162	h
-2.69364932217800	13.98407434023905	9.01742881582322	h
0.26591294306801	6.21778844062886	-5.97375340154905	o
-1.50286456821560	5.99359597094737	-5.39477516903659	h
0.18467035105915	7.38080137856404	-7.46638167188371	h
2.80219465033525	1.70415928110162	9.22421538913498	o
4.21116261156948	2.50313717013740	10.19695239219136	h
1.97102268437350	3.06320178733755	8.27435716826499	h
7.16741403975123	0.10910561133130	-5.66996293770120	o
5.42763559057767	0.74244288720791	-6.08350576160875	h
7.57538713492837	0.99576742944738	-4.08145781017702	h
3.75559562710389	-13.51827743656964	-1.50466939572129	o
3.65408916192827	-13.24815926343876	-3.38682081687801	h
2.33002092786397	-14.65696683644511	-1.07955775539503	h
11.36812748086164	-11.80911280268977	-8.08151892579779	o
11.76859833959289	-12.52327709001011	-6.40622263190865	h
12.79798499369397	-10.67461804706130	-8.47217364436814	h
8.64979395850609	-9.00627415625354	1.95912229263799	o
9.48175285258450	-7.86481761443543	0.69790806760823	h
8.92177989781394	-10.78343283542164	1.37873868794684	h
14.48635363052163	2.55501215205204	-6.53769792371178	o
15.87650414057415	3.21117271939509	-7.54870988738233	h
13.32291017107646	1.66976071779886	-7.75090980950182	h
12.78964568707042	-13.15528259786199	-3.02190680573500	o
13.91057410839397	-14.59733408271252	-2.80944975528123	h
11.28905833145706	-13.51043779261552	-1.89881420489985	h
18.43247123233860	-4.88269608524628	-1.01281082750371	o
17.24627951695791	-6.11437512992385	-1.75266974945609	h
18.00849219240967	-3.21551948954583	-1.78957492263882	h
15.30832832691063	-7.52908054551966	6.69271107407551	o
16.12984778000868	-9.13320888017240	6.33800462828887	h
16.26504856572122	-6.24396796580897	5.70233749439306	h
18.03176393049380	-4.03559453258653	3.86383664415618	o
19.73012410743506	-3.71387808807052	4.49347717545349	h
18.23337705158897	-4.47994258541238	1.98751542184029	h
7.02737024277141	-9.17233269772224	-8.38818942491459	o
8.65035034505134	-10.14929922801897	-8.15739967351988	h
7.07908711011790	-7.71547240550207	-7.20119424836560	h
17.18204732358159	-0.02671070477537	-2.37439997904719	o
16.12905149588095	0.69723715702881	-3.72114660571860	h
16.46082684405209	0.54651619788131	-0.74878879781305	h
10.52499616988002	-5.62616681533177	-1.42085328046674	o
9.22374205894586	-5.50093160457508	-2.78694080197529	h
12.10037576929922	-6.29749323316599	-2.21509216202135	h
15.03904329918090	-7.93762827787277	-8.59506658414140	o
14.05987483203836	-6.57607388466109	-9.55661058812239	h
16.70002390206871	-8.02120740345642	-9.37788690374417	h
9.78875964980712	7.25330767467424	3.92776424661023	o
9.96464005337296	8.85800817008350	2.97097120110414	h
9.08082430295662	6.05567871169928	2.69018268374370	h
10.12531796513764	-7.58830959895563	6.72446857391376	o
9.65500120146899	-8.21501702472193	5.00478301123207	h
12.01442378481564	-7.54027884605389	6.75345942070382	h

3.48469002654753	-12.74567628064419	-6.54443790906349	o
1.78961177407588	-12.40880486598507	-7.22634025451773	h
4.63549551187527	-11.47175489512385	-7.29371260778745	h
2.10331282702327	-5.55959735900454	-2.78440654983615	o
0.84658962712983	-6.24116034610018	-3.97121281920398	h
2.52446418335803	-6.88650653415678	-1.48217876295465	h
12.43084350542474	-4.61497291808246	-11.18068114894545	o
10.86431759362243	-5.32983829773781	-11.90252611413256	h
11.93855542943869	-2.97504738376469	-10.40658668136147	h
1.98462514768981	9.27646784683300	7.42704895478037	o
3.84773024676647	8.89265621937136	7.72668111192203	h
1.20047698790571	7.64490473017375	6.99716128980456	h
8.05823266121371	-2.72424222087795	6.34280064242156	o
8.78146004991989	-4.42409596222251	6.70718976656210	h
8.83091515067236	-2.27190540627561	4.68661158538922	h
3.62408005225568	-8.87107752186237	0.76096385514310	o
5.47058180227294	-8.77044936650154	1.17780724643555	h
3.41784512839446	-10.63172365355033	0.09127439780396	h
6.38712327586423	-15.55939588188605	7.95581145558601	o
6.98011171733770	-13.99270934172799	8.75945740411001	h
4.50674453820748	-15.38365697103766	7.88115020885522	h
6.95945724061052	3.88208182585091	11.36226027441222	o
7.23211927428027	4.03460691189216	13.17376944205101	h
8.41595908540852	2.76183161016026	10.67275582632257	h
9.67451040819562	11.75341297498314	1.03871787432519	o
10.60004173061176	11.65935076596572	-0.66361421079563	h
10.32946983900544	13.24514101496631	1.89246464231903	h
10.37682484389957	-1.49621832295873	1.75878647570189	o
9.50666372560316	-0.07769642910760	0.91209538243796	h
10.47569310417866	-2.90982556396852	0.51275866207204	h
8.54271854150978	13.22484350606151	-7.20082776497368	o
9.12550455979473	14.50857706323923	-8.37967550390151	h
6.89244771033754	13.87539019300567	-6.46399749296260	h
-8.98234176149835	-4.30047147248427	9.12433685970314	o
-9.47694138249104	-5.76210274324930	8.04159564082994	h
-8.85345971682665	-4.95026272587514	10.83996570082369	h
0.09812378044304	-2.57165262611630	9.68472735093472	o
0.68813442520088	-0.82388187267275	10.01634203650226	h
-1.59110921657083	-2.46041265749033	8.87341923926505	h
1.29094789900053	-14.50916645160221	8.16433878389705	o
-0.04960710019750	-14.68123018819244	6.86972308090376	h
1.36260253892016	-12.69514965064654	8.57176720810152	h
1.89358506479195	-6.37832941857572	5.01146912768827	o
2.54011338205906	-7.42495553219631	3.58899616402242	h
2.06302899744004	-7.47289155109038	6.55315338202008	h
0.96032403156396	-6.27543180612357	13.14986369343845	o
-0.43719917234161	-6.54763140221080	14.30857839233397	h
0.50321689050871	-4.78834052910554	12.08345329486088	h
-4.08936697437675	-14.12934427787768	-3.82189323452212	o
-3.29620152584374	-13.42696627806883	-5.35869384491001	h
-4.95612657955116	-12.68770452224764	-3.00851945197637	h
-0.42560082413399	9.38807443888271	-10.03596718364409	o
-2.19506812049427	9.21604486607420	-10.61537068665893	h
-0.24419787990368	11.16490337041673	-9.45801536491657	h
-5.69265653861001	3.69865051173597	-9.27978040092372	o
-4.69663078120297	2.52832991043439	-10.39621908836108	h
-5.51107430576565	5.42749839952650	-10.01500098686431	h
-7.41333821061352	6.12905091059835	-15.85908774403240	o
-8.12202464873377	6.81653615653215	-17.40935180657115	h
-8.49636663505840	4.67561154765979	-15.38650663619852	h
6.90772882919905	6.04503255502379	-13.34830968959015	o
5.15156635530458	6.52644915114145	-13.85257230345952	h
7.22729369662460	6.81609693946750	-11.66111020418669	h
2.09411452322917	7.55954301439484	-14.55011228835969	o
0.77916124452636	6.43362380678657	-15.25097170274276	h
1.34408465872298	8.27880191440989	-13.00655000194034	h
14.69549912130066	-8.05234259769756	-3.35685311384594	o

14.24691139502939 -9.84055925087616 -3.05281737780182 h
14.91788003944182 -7.89998649454079 -5.21887006374556 h
6.82539656243459 -5.08129839289504 -5.00658873157585 o
5.10875161558900 -5.24387741621897 -4.24659359100224 h
6.98683451915837 -3.24667043588986 -5.41041370188050 h
2.66432760159373 7.09027055006564 -1.48952940922943 o
4.03739340265373 5.84640461861585 -1.27998637682039 h
2.02587233533053 6.87076810506455 -3.25510342005660 h
10.92833537826368 0.10775969418919 -9.34029352695191 o
10.10913363266429 0.76515074965180 -10.91910771986858 h
9.54601528655891 0.12149066457766 -8.06279434905187 h
-5.51217185355131 8.45885581836509 -11.40500482792454 o
-6.74272249606381 9.60068729356359 -10.54985966517282 h
-6.18867458338195 8.05769577189812 -13.09420243555026 h
$end

Cartesian coordinates in atomic units of the most stable forms of the (Glycerol) $_2$(H2O)$_{108}$ cluster (structure 1 in Table S4 in SI)

$coord
4.46997926578880 6.32278531930159 0.04774448917045 c
1.80486343226111 7.34356989880432 0.55540770467246 c
-0.12345060703505 5.59663335674869 -0.30416366247801 o
1.32834132628252 9.87491570532252 -0.76880301666394 c
-1.24086834358953 10.73224025969987 -0.35438528045081 o
4.74385514911049 3.93384543479040 1.36705543310816 o
1.57069145179074 7.61378767509471 2.60672316212802 h
-0.35115146348444 4.25830074886168 1.00360598762936 h
1.57964213287683 9.67457253706189 -2.81555881310008 h
2.68109954828659 11.30030794010092 -0.10317832558638 h
-1.38339620034537 11.50653506900819 1.38367231014369 h
6.38182169602674 3.12756735712426 0.86969113623286 h
5.88578740244405 7.69307856010615 0.71405763851041 h
4.74822845103735 6.03707686794034 -1.99066263020322 h
1.33777089575749 2.18764836115816 9.99312083992526 c
1.54062862209918 2.04962564235562 7.09577580466210 c
3.59097105753123 3.74016884448757 6.33719247205824 o
-0.98015889075904 2.70633968953071 5.83280751460271 c
-0.99527062091445 1.90731115086567 3.18347954222757 o
3.40086991621075 0.87929707099374 11.20182155701736 o
2.05779724763174 0.10716794021747 6.56175839689157 h
3.90453175093706 3.67145756072653 4.46336468584512 h
-2.52567415314577 1.74448378809568 6.83129991509490 h
-1.32703204156326 4.75542258986720 5.93818287407082 h
-2.76397573466772 1.34226345343106 2.78837719810180 h
2.88048863840380 -0.93795329583824 11.26128568165038 h
-0.47367046922418 1.37086884289358 10.59930213813365 h
1.38706074889108 4.16399397723550 10.61830908357808 h
8.76337772206743 2.38783375833288 -13.33509238010924 o
8.87443055957668 0.63363781212043 -12.68876222623497 h
7.95564834996867 3.37886796102754 -11.94738493185853 h
9.12256846112393 12.98894038451486 12.88938641270171 o
7.78400627158369 11.70535596838246 13.10106171319541 h
10.67993061476625 12.00560226815977 13.12301409823775 h
3.86687202553163 8.65458732615919 8.28768750493848 o
3.61595363249117 6.90912337629900 7.65295702186852 h
4.60884639024791 8.52496201733431 10.02354711374901 h
12.13849116313778 4.91208458106463 -2.57212536352096 o
12.86508562004883 6.25538569070558 -1.44755205488994 h
11.23245262245733 5.85913642350360 -3.93308009932216 h

7.44222769697676	13.63169730900535	0.34131228785448	o
9.27653591539369	13.98197314609481	0.44331730105085	h
7.09554979715445	12.69350054154726	1.92754665541515	h
12.77080775467010	14.02506490936325	1.08868288099538	o
13.77543163992929	15.15482791080329	0.04151851143363	h
13.10938387653240	14.53980044627229	2.87372632289126	h
-12.69766227792844	11.26952682756232	2.17428857214581	o
-11.40545029455804	9.98813488669701	2.59626099218688	h
-12.05842400831819	12.84521657815450	2.93719720669147	h
0.39276543504393	12.51716639534797	8.64367262828089	o
1.57902167326484	11.06637668596100	8.60879714824117	h
1.44389865504919	14.09391294983566	8.82328608425727	h
7.42801938824077	-8.55426424883466	8.78769397808247	o
5.90800318076704	-8.70225856871129	7.71815768793507	h
8.69858147955099	-7.85554955947655	7.61709459534096	h
14.87617377408299	1.43086303103424	-5.42302448122426	o
14.07152867897136	2.74600367904150	-4.34400684894311	h
16.43440785848296	0.96535156299666	-4.56992643023751	h
-14.89141125996844	-1.67448470945046	0.20263428575132	o
-14.05611184317638	-0.94812497992558	-1.29401802877191	h
-14.42696525607457	-0.66049164725872	1.69679290042194	h
13.80167742229094	8.44182751392696	0.79200734889442	o
12.94129698713854	8.05379821735933	2.40705701913162	h
13.57683710852206	10.27281555758999	0.59118149840178	h
9.22271682317677	1.58537363751724	0.15082361102068	o
9.98302157490259	1.01245470828739	1.75608683244056	h
10.39747113866035	2.78324688262250	-0.73891757157789	h
-1.26588828077526	-6.39857524276816	7.90204905073452	o
-2.37382465223748	-7.60322690568418	8.82208057907539	h
-2.38909640598101	-5.49440091906849	6.69942443113798	h
0.60955827761351	0.73493316964947	-11.80557827638217	o
-1.12704994117739	0.25768485001982	-11.26825814256093	h
1.74677038066105	-0.62774661975601	-11.21404890086952	h
1.42960645711111	-3.81756315044798	11.58560853436525	o
0.51271232300633	-4.65393688829106	10.17365872752881	h
2.87279398972198	-4.96143904492457	12.08067699395450	h
13.20508336423267	4.96278522563254	-13.98121807921101	o
11.67430053411281	3.86617655839003	-13.75190014654319	h
13.20420457876033	5.38709933081631	-15.77016126009659	h
9.90353374402776	5.00508361546162	12.53885075662509	o
9.72851887692838	4.31166474919012	10.79098159123161	h
9.03196528272831	3.76543413444418	13.64714206968744	h
7.18011581237553	11.28006269146452	5.15132164988175	o
7.60279255565804	12.75465724578256	6.27215415390160	h
5.85507507195753	10.35987561079054	6.11547519649583	h
6.11167133968386	-7.44808958938929	-2.68793149649453	o
6.82027870214078	-5.70119795795448	-2.60363617959522	h
5.31284439775574	-7.58461168522124	-4.36575911340685	h
-3.01329838107828	20.61877862420915	0.99571650496892	o
-1.82332505556518	19.77804284138621	2.24010809537496	h
-3.11607328170729	22.38188743956898	1.50948087211486	h
11.66251858540935	-2.42933775305078	-6.98881810029939	o
10.51967573362412	-2.58676131165414	-5.51331350862934	h
12.85672472654806	-1.04472923011909	-6.55271171317904	h
-4.23551922594512	-5.17542101731457	-11.59029644768948	o
-4.44995153069731	-3.34442211717964	-11.22962287702961	h
-5.82845814279148	-5.72911599803635	-12.31714326882142	h
10.08306412589086	-6.34132742512202	4.41375510503561	o
8.47609301093719	-5.60866552120914	3.82133405356251	h
10.51695713642443	-7.54852701512003	3.05362232957909	h
-8.93530180369432	3.15783309320483	5.12393467943023	o
-10.67049058236891	2.46920466597975	5.07659646046788	h
-8.22715778977957	2.70359057193700	6.85404733924995	h
5.38269232256149	5.10371558842864	-16.81565332079128	o
6.42940710368431	3.82295112992744	-15.96159046907273	h
6.11227364048999	6.70491464132264	-16.21330506711128	h
-3.60471533140678	-0.19631927763746	-5.01048568170434	o

-3.76471489022378	-2.00267519387596	-4.47172356513288	h
-4.90310355035682	0.78054960493558	-4.04902540795919	h
1.49127218164515	5.32339694687369	-9.35810327270116	o
0.97174368175763	4.79248263565573	-7.62680024478861	h
1.19014180991037	3.77270472941487	-10.38865106666124	h
0.25892232732481	3.86525523125862	-16.10030980001082	o
1.99790116810397	4.49842546452830	-16.44833909599440	h
0.49746123855669	2.48763841588488	-14.86469491065252	h
9.77889080317669	7.67091731884644	-6.36584691320314	o
10.88611716317538	8.32163184688383	-7.72360829507221	h
8.76537279440053	9.14411393868487	-5.69027925233770	h
-6.32030349572512	-5.71394114009870	14.19629637524186	o
-4.75630780554397	-4.97996573932761	14.90794785262543	h
-5.76194547885548	-7.22026248378782	13.26136796472134	h
-0.22487274764504	18.54368977056726	-3.13210207645374	o
-1.28566839067932	16.98047314292592	-3.17028928741416	h
-1.14645107487205	19.68887347495357	-1.97905298150646	h
-6.98538735747438	2.67414409692721	-2.42561934520235	o
-6.34522703090452	4.42385534852141	-2.10067345409934	h
-8.35947167939258	2.88700288077715	-3.67522193785347	h
-4.30418623325283	-0.12120441435473	-10.08605285630749	o
-4.10235333112934	-0.18591684743001	-8.20403827893645	h
-5.18251063465778	1.50651840383954	-10.37556051888054	h
0.35046739665539	3.20753678041325	-4.81164929971704	o
-1.06527267249633	1.96893304768239	-4.86385955633029	h
0.17390697303860	4.16592807223858	-3.19296853649206	h
8.37288959025941	15.23567355577281	8.26398332813878	o
10.04983855028435	15.72005706118934	7.61308865039087	h
8.69932002884469	14.52503097449459	9.99122436879795	h
-2.02570114600807	13.88673486942082	-8.76925414942575	o
-0.84430929874104	15.33139764387398	-8.92819910708967	h
-1.33031130368676	12.38968465636957	-9.70204228410389	h
6.62566124573918	5.64626148269140	-9.96072056879357	o
7.59649834485955	6.19755179084308	-8.44078530242604	h
4.79334514992774	5.57423269045186	-9.52677108393089	h
-8.16608558680709	15.19367106828462	-3.71478046626174	o
-8.26370215889271	15.37213388099193	-5.56154518867519	h
-6.37812894651128	14.65070173103508	-3.47508185273053	h
-9.54006612759961	-5.39335106361627	-3.40891526948016	o
-10.58860915518864	-6.01615686248681	-2.00138470534024	h
-10.42189016791286	-3.86510341767777	-3.99681886971642	h
-7.16148003402414	8.88223762487934	-6.83195096640799	o
-9.02505996140867	8.68908849345573	-6.73249877029060	h
-6.95895820551790	10.58053170523456	-7.58898633431141	h
3.84474365251334	12.82794625570317	-12.77991399443627	o
2.36667303819189	11.71742280767439	-12.56525452546582	h
5.21029425546022	11.67867220649076	-13.34628850212883	h
12.40489978477546	9.05546898343249	-10.98327893815387	o
12.88389652433245	7.52190277543488	-12.00177651924161	h
13.90529712710716	10.11648245553397	-10.94071600688270	h
3.96181406443108	17.76830445493901	0.32655022575138	o
2.94255151512107	17.82889432816977	-1.22741089115902	h
5.21029482849284	16.38583036653348	0.17110401776955	h
14.57516804340119	10.72906062534437	8.12929319394293	o
13.52362741781962	9.51708696874670	7.15019017438425	h
14.23894046230430	10.29296736441177	9.90835322586562	h
3.14888435722858	-7.96910192994758	5.51559679508517	o
2.84349509130853	-8.83759752695251	3.88408353110662	h
1.47385684104134	-7.65623310204975	6.29886772842820	h
2.35343163754226	-9.51148047337638	0.48456767645485	o
3.72677315247909	-8.74934759360975	-0.54977574960333	h
1.87693345820745	-11.12485457929792	-0.36973588473653	h
-0.46407933691427	9.74407841554470	-11.38682420655498	o
-1.65651104400577	9.01062879640899	-12.62830102105025	h
0.25947219577410	8.23000280346322	-10.52498217540138	h
-13.84138663266333	0.81798045705534	4.96342044751899	o
-15.17714857327413	2.00809367364625	5.39650268299389	h

-13.83942532639479	-0.47531215444072	6.34318662752900	h
-5.81105686080593	0.30873384096706	2.25049178642473	o
-7.01032581828945	1.30514374008568	3.34494724533083	h
-6.35039515552975	0.75713296980811	0.51749185295827	h
1.93499137980034	-1.93890485637102	0.24092267108094	o
2.47788226448866	-1.53143205752656	-1.51429199844628	h
1.26218222311957	-0.39028869837418	1.02195811746618	h
3.96030440796824	-2.80125195823134	-9.46720568215584	o
3.68632024385385	-4.61377398721407	-9.09830861273042	h
5.73685785259021	-2.67691018179507	-10.09083770459448	h
10.18787343562612	-9.71057115754881	0.16585628105132	o
8.89905546439568	-9.12025700056423	-1.04930443936577	h
9.68188331653765	-11.42321625448904	0.60039938223470	h
-1.36046959006277	-5.92835521155492	0.24942810689999	o
-0.17873342912344	-7.40659480038455	0.31540063470273	h
-0.21145799344961	-4.42860236862800	0.21366421111211	h
-6.77717469679482	1.46894532313359	9.29401817373434	o
-7.42135587561298	-0.25205715906539	9.64927813597436	h
-6.83309786469565	2.36603402933593	10.96217438508003	h
-4.37147420175394	-5.18314096946820	-3.82605822935680	o
-6.22673906543341	-5.39871474818155	-3.53374604770566	h
-3.43865655151930	-5.62369931262467	-2.24368966190960	h
-10.50824036800962	3.31803274237576	-6.49212741848536	o
-9.50443062663726	3.48114549497544	-8.04910614686291	h
-11.37288377028477	4.98035688156327	-6.26098004046012	h
8.11516726126863	-2.55001867868501	-2.98667449742842	o
6.60542355285799	-1.64462236986802	-3.65970546392314	h
8.74609733275924	-1.34751362442204	-1.69234767985627	h
8.94419522877696	-2.42522279094187	-11.06973092776144	o
9.61736843903996	-3.77972736919681	-12.11561663793393	h
10.09669615095860	-2.35040435516118	-9.49651815089935	h
-4.88687200243509	-9.42345182234151	10.34359606296739	o
-6.21093928454129	-8.97945910704113	9.05426706524118	h
-4.79511602316111	-11.25804095119753	10.41139347865063	h
-5.69757808308831	7.65696614165709	-1.66997728169059	o
-6.09848666503195	8.40371989682950	-3.34174831558245	h
-3.97373418710644	8.23112030542807	-1.22942702585176	h
-4.54215168010611	-4.64127632363605	4.06458923128507	o
-3.46439842115595	-5.29418477895954	2.65552844180432	h
-5.11637920950408	-2.95175594973522	3.49569511655612	h
3.62272121035846	-0.74321733518972	-4.65047923147702	o
2.75718132333053	0.92276550644481	-4.79205556574323	h
3.60724968242205	-1.48792804003110	-6.38333506685803	h
-4.27835239040134	11.48957323847139	10.84673444427692	o
-2.55154568099331	11.95900700003817	10.28999806385023	h
-5.32425375847750	11.55482187764754	9.30378300072089	h
2.55245795146381	-5.80214654734649	18.05501903579097	o
3.97668063942265	-4.59990255174804	18.06606023767541	h
3.07364256349913	-6.99557778775164	16.74051985019985	h
-1.88993786280702	-12.84322667075337	-6.49123926013745	o
-2.10347985154677	-11.03970931723863	-7.00468051787644	h
-3.56981077853082	-13.58323311241466	-6.57112996985559	h
-1.85531002813208	13.09485099758466	4.10632460679827	o
-0.90541130691032	12.72258972212123	5.71197635856753	h
-3.66102333370560	12.85460863238018	4.61817485535565	h
13.13239773371723	9.11009623965659	13.13316349464134	o
12.15518321448820	7.50450925015779	13.03112290263331	h
14.43682875355509	8.82924449731062	14.39710762851174	h
3.06517406568959	16.88902532598096	8.54120160165166	o
2.34121851156885	17.67319292550154	7.01334290373284	h
4.89929478612684	16.73305192154353	8.27347124739130	h
-12.76963092079847	-7.52410873468135	5.57746645728483	o
-13.04600593209871	-7.20449428946466	3.75329127573500	h
-13.43698668767962	-6.04211841843281	6.47795248943235	h
6.03256385326776	8.57293525865214	12.96785906035735	o
5.07419214308077	7.99566026977980	14.47420115707653	h
7.45447466755115	7.34869813377142	12.81482779548681	h

-13.40771535363241	-6.48083475460779	0.38609693229087	o
-14.07162079741239	-4.68449000264514	0.34909388749108	h
-14.73514043873603	-7.52457941816757	-0.34432072563698	h
9.84790301730530	-0.85495310134615	4.89045848149518	o
8.13725893529256	-1.62305022797769	4.72740502130133	h
10.93359856149680	-2.35846302778207	4.98531226579530	h
3.86771252077434	6.26610507116088	17.23154703678906	o
1.94360864865133	6.08412665455638	17.24702472641702	h
4.29767287177215	7.03807456909430	18.84454276279943	h
7.55411815290965	9.15354187731257	-13.70855933250985	o
9.28173815402028	9.66939579140050	-13.26457001601047	h
7.11357503402935	7.97973709907029	-12.29725574367335	h
13.45120991444524	15.19101673583524	6.18766680003994	o
13.95307833728169	13.53051537007774	7.03874858009616	h
14.75827829396530	16.40206456726071	6.64377479937121	h
-1.04068871415575	5.78303874485687	17.37906623524486	o
-1.78188308791104	4.06922115008874	17.43034026470295	h
-2.21385081799868	6.84545536204059	16.37708578962297	h
5.42721188622880	-6.56234970156396	13.14672942243408	o
6.40151961912299	-7.26765638659948	11.68409761100095	h
6.52224939187188	-5.37774497354405	14.06584300585081	h
-4.81882282919993	8.46588962350132	14.75399822153645	o
-4.57954727167810	9.53959857229004	13.17344283732966	h
-5.73347143024083	9.55531748113216	15.91989524429246	h
8.94045683194535	3.46221820076495	7.66437425991843	o
7.08129368791724	3.41227619301960	7.46112703986425	h
9.53125853746965	1.83870039496316	6.91851293687584	h
-3.51532619537039	1.03469166784564	17.42327623596327	o
-4.34355661358678	0.60468564967383	19.00823540251365	h
-2.71637199147268	-0.58546721818495	16.81976203552268	h
6.38583686825791	2.02189933848915	15.22277609724340	o
5.50114115899061	3.33037309151983	16.23501697109428	h
5.22882601629562	1.70493252892633	13.75373772241681	h
7.00581380725388	-2.85890776252116	16.91296516785608	o
6.83148824852281	-1.03449937648879	16.44263084468254	h
8.52760939354438	-2.97224566688768	17.93755481191543	h
5.46786357907101	-3.56754498842486	3.89430353729735	o
4.42213773781442	-3.00887848169469	2.44671841058385	h
4.52581501542099	-5.00182097621582	4.67378739996451	h
-8.48213503981053	8.03093492928722	3.06139405414812	o
-7.62525205320997	7.87001969808283	1.40938676633006	h
-8.62221635902175	6.30957580965855	3.80413056314123	h
-12.71988771317302	-1.02008804543142	-4.72716737272776	o
-14.07932249733307	-1.51723359089845	-5.86244229606523	h
-11.97932372337360	0.53625964070331	-5.49397485182596	h
-7.06371999925094	13.92577132192854	-8.93766585888959	o
-5.18555182393972	14.15854870870868	-9.11949237910872	h
-7.78479501406515	14.23353582732749	-10.59976555223431	h
6.68907023085087	11.24610805194025	-4.56889297829096	o
6.16290217523524	12.57735009082242	-5.80911668478589	h
6.99339235616456	12.09412464654374	-2.94029824055632	h
-6.93798332879834	3.59380737560079	13.96791646876747	o
-6.36603119607230	5.36184433158854	14.16029134916079	h
-5.87463661996814	2.60910231659190	15.13582545430202	h
-12.35745981405541	8.14434717527022	-6.08522121229270	o
-13.63897203544578	8.63373242599409	-7.30874896520638	h
-12.42442319555599	9.51652245329878	-4.67519360726846	h
0.29463088376746	18.00123428818350	3.85355338210901	o
-0.55451646033516	16.32690593845446	3.88175333966390	h
1.74177037590709	17.82051178328684	2.62891755961063	h
5.38637299769715	14.72818478295479	-8.15512336162946	o
4.13593643094942	16.07352172327420	-7.90768943037292	h
4.91939233232918	13.99759782416454	-9.83207069923666	h
-13.61884964505060	-2.90483416643306	8.64273821344783	o
-14.88074868370965	-2.97533203786670	9.97914378575704	h
-11.92047284563079	-3.00605895493594	9.52377294545885	h
-10.07561055596522	15.61087643498353	4.27321907430083	o

-9.27924559033057	16.70414798287949	2.91451470997416	h
-10.95422792314038	16.76873373384718	5.39864113457356	h
-1.61041828227189	-3.45372473416874	15.82205746770844	o
-0.66321526142741	-3.43069919727622	14.18883757619694	h
-0.36885371928455	-4.31231508671115	16.95257879513209	h
-3.55150581663812	6.70833350906208	-14.56692559712178	o
-4.36120721180007	7.31047735507444	-16.10475784364199	h
-2.06857675754406	5.62264501630733	-15.18400969686866	h
-3.01311087008186	14.19131047437844	-3.64703505758616	o
-2.53685188985137	13.73657119425104	-5.40829193353402	h
-2.31360161205928	12.86501484162209	-2.49272581497218	h
-8.05995947572108	18.44484708962787	0.61632863182015	o
-8.20118168298247	17.61971634884733	-1.05173353158017	h
-6.32521565713153	19.12100564542009	0.74129895702340	h
-12.35579210394961	11.80872192925866	-2.91171025231819	o
-10.87016226772613	12.92975817899374	-3.08992521105091	h
-12.62136969163126	11.59134103038535	-1.04433838353610	h
-6.57790738141427	4.78793594092989	-10.42610229131882	o
-5.77118005735956	5.40877275644987	-11.99911983548072	h
-6.33531473283470	6.17394314458641	-9.19247861226254	h
-7.91154037418892	-7.26707011569850	6.95144491816001	o
-9.67600103817260	-7.57040818599926	6.29966328763859	h
-6.88192156940831	-6.48929110174125	5.58198134850452	h
11.13998509703975	7.84847131441068	5.37444057337036	o
10.43560097505204	6.25882313364185	6.06604805014258	h
9.69059697876295	9.05455728604080	5.26258733189858	h
1.04375850313982	18.16037626860725	-8.05827085495730	o
1.18996040990959	19.67177841933989	-9.09199244337705	h
0.57251537232575	18.71502731225588	-6.31129115218004	h
3.15589208528710	-7.73124427485126	-7.51442337629570	o
1.25815127421382	-7.70469246844025	-7.42919727546560	h
3.55245070919096	-9.21945625081416	-8.52229965487300	h
-2.04047790954419	-7.76988883730427	-7.61072929188691	o
-2.93540665014842	-6.88126587205103	-6.19361583303541	h
-2.76429811177732	-6.98348303944644	-9.16586255784945	h
0.75824387462188	-13.72180071608563	-2.08682076027933	o
1.82885274734940	-15.16529214215627	-2.46318469048193	h
-0.22384633568997	-13.38806458657867	-3.66169449523202	h
-8.86913927729532	-3.38283722289923	10.32351691921594	o
-8.12330671858949	-4.01447916532993	11.94963480344093	h
-8.38970208428199	-4.71844499082720	9.07775505641679	h
-6.58081519969722	12.01430843374408	5.95039468783319	o
-7.82922589448203	13.36750022948571	5.58552144835982	h
-7.21396027795079	10.49429488696377	5.04127602825951	h

$end
