# Supplementary material for: Anomalous Water Fluorescence Induced by Solutes
Source: J Phys Chem Lett. 2025 Jun 30;16(27):6935–45. doi: 10.1021/acs.jpclett.5c01189 (PMC12257589; doi:10.1021/acs.jpclett.5c01189)
Supplement: Supplementary file 2 [file jz5c01189_si_002.pdf]

Supporting Information for
Anomalous water fluorescence induced by solutes.

Anna Maria Villa, Jessica Di Paola, Diletta Ami, Luca De Gioia, Antonino Natalello, Luca Bertini

Department of Biotechnologies and Biosciences, University of Milano-Bicocca, Piazza della Scienza 2, 20126 Milan, Italy.

Luca Bertini

Email: luca.bertini@unimib.it

Absorption emission spectra.

The absorption spectra in range 200-400 nm for H_2O and the solutions of the 21 solutes are shown in the following figures: H_2O MilliQ milliQ water (Merck Millipore Ultrapure Water, ρ = 18.2 MΩ·cm; Total Organic Carbon ≤ 5 ppb) (fig. S1), HCl (fig. S3), NaOH (fig. S6), KOH (fig. S9), KCl (fig. S12) NaCl (fig. 2 - main text) $CaCl_2$, (fig. S15) $MgCl_2$,(fig. S18) $MgSO_4$ (fig. S21); 2:1 salts (K_2SO_4, (fig. S24) Na_2SO_4 (fig. S26) $(NH_4)_2SO_4$ (fig. S28) L-lysine (fig. S31) L-glycine (fig. S36), D-lactose monohydrate (fig. S38), D-(+)-glucose (fig. S42), D-(+)-Trealose dihydrate (fig. S47), xylitol (fig. S50), D-sorbitol (fig. S53), glycerol (fig. 2 - main text),urea (fig. S57) and Ethanol (fig. S61)..

Fluorescence emission spectra.

Among the examined ionic solutes we included: acid and basis (HCl, NaOH, KOH); 1:1 salts (KCl, NaCl); 1:2 salts ($CaCl_2$, $MgCl_2$, $MgSO_4$); 2:1 salts (K_2SO_4, Na_2SO_4, $(NH_4)_2SO_4$); non aromatic amino acids (L-lysine, L-glycine); carbohydrates (lactose, glucose, trealose); alditols (xylitol, sorbitol, glycerol); diamide and alcohol (urea and ethanol). HCl (Sigma-Aldrich, 37%,), NaOH (Sigma-Aldrich, ≥98%, pellets anhydrous), KOH (Sigma-Aldrich, anhydrous 99.99%), KCl (Sigma-Aldrich, ≥99.5%), NaCl (Sigma-Aldrich, ≥99.5%), $CaCl_2$ (Sigma-Aldrich, anhydrous, ≥93%), $MgCl_2$, (Sigma-Aldrich, ACS reagent, 99.0-102.0%), $MgSO_4$ (Sigma-Aldrich ≥99.5%), K_2SO_4, (Sigma-Aldrich, ≥99%) Na_2SO_4 (Sigma-Aldrich, ≥99%), $(NH_4)_2SO_4$, (Sigma-Aldrich, ACS reagent ≥99%), L-lysine monohydrochloride (Fluka BioUltra, ≥99.5%), L-glycine (BioUltra, ≥99%), D-lactose monohydrate (Sigma BioUltra ≥99.5%), D-(+)-glucose (Sigma ≥99.5%), D-(+)-Trealose dihydrate (Sigma≥98.5%), xylitol (Sigma-Aldrich ≥99%), D-sorbitol (Sigma BioUltra ≥99.5%), glycerol (Sigma-Aldrich, ACS reagent, ≥99.5%) and urea (Sigma-Aldrich, ≥98%) solutions in milliQ water (Merck Millipore Ultrapure Water, ρ = 18.2 MΩ·cm; Total Organic Carbon ≤ 5 ppb)

1. HCl solution shows a single emission band with a maximum at 300 nm (exc = 220 nm). (Fig. S5)
2. When excited at 227 nm, NaOH presents two emission bands at 300 nm and 395 nm; when excited at 240 nm or 308 nm only the 400 nm band is observed. (Fig. S8)
3. KOH shows a weak band at 300 nm and a large band with maximum at 400 nm and 415 nm (Fig. S10 and S11)
4. KCl presents two emission bands peaked at 300 nm and 420 nm (exc=227 nm); similarly, the two emission bands of NaCl are peaked at 295 nm and 430 nm (exc=227 nm). (Fig. S13-S14, and main text - Figure 2, respectively)
5. $CaCl_2$ presents an emission maximum at 405 nm (exc=320 nm); $MgCl_2$ emissions are peaked at 300 nm (exc=220 nm) and 427 nm (exc=310 nm) (Fig. S17 and S20)
6. K_2SO_4 emission shows three bands at 305 nm, 360 nm and 420 nm (exc=227 nm) (Fig.S25); Na_2SO_4, emission presents two bands at 302 nm and 425 nm (exc=227) and

a single band at 425 nm when excited at 320 nm) (Fig.S27); $MgSO_4$ emission band presents a shoulder at 303 nm and a maximum at 340 nm (exc=227 nm) (Fig.S23); $(NH_4)_2SO_4$ emission spectrum shows a weak band at 310 nm and a strong emission at 377 nm with excitation at 210 nm. A single band at 377 nm or 390 nm is observed for 258 nm and 308 nm excitation, respectively. (Fig. S29 and S30)

7. L-lysine and L-glycine present very similar large emission bands with maximum between 415-440 nm (exc=245, and 338 nm) (Fig. S33 and S37)
8. Glucose (Fig. S44) and trealose (Fig. S49), when excited at 220 nm, show an emission band at 295 nm for glucose and at 302 nm for trealose. In addition, glucose shows two other bands at 340 nm and 425 nm when excited at 227 nm and 330 nm, respectively. Lactose emission band presents a maximum at 330 nm with a shoulder at 300 nm (exc=220-227 nm) (Fig. S40-S41)
9. Xylitol emission presents a maximum at 300 nm and a large shoulder at 345 nm (exc=220 nm) (Fig. S52); the emission spectrum of sorbitol shows a large band peaked at 3250 nm with a high shoulder at 306 nm (exc=2250 nm) (Fig. S55, S56), glycerol emission presents a band peaked at 305 nm (exc=220-227 nm), and a band at 400 nm (exc=295 nm) (Fig. main text - Figure 2)
10. Ethanol presents a maximum at 285 nm (exc=213 nm) (Fig. S63)

Water absorption and fluorescence spectra

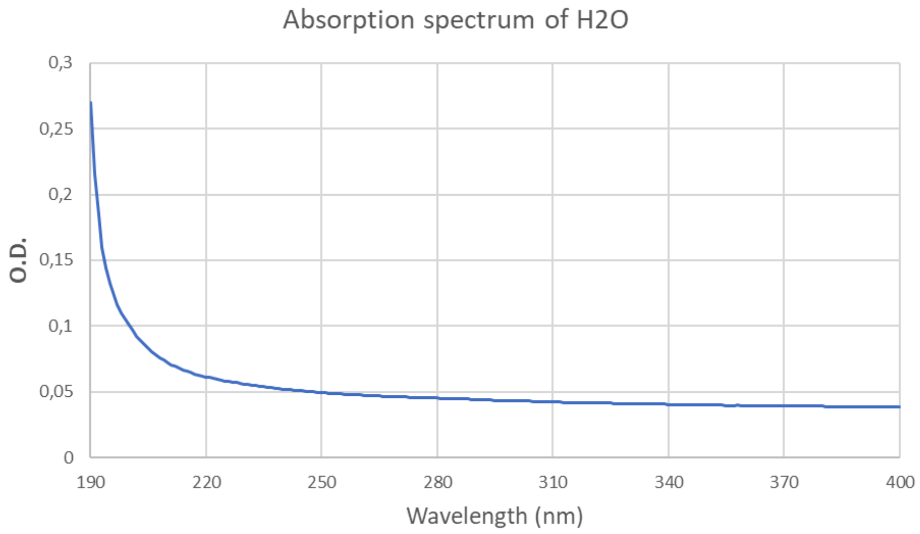

Fig. S1. Absorption spectrum of water

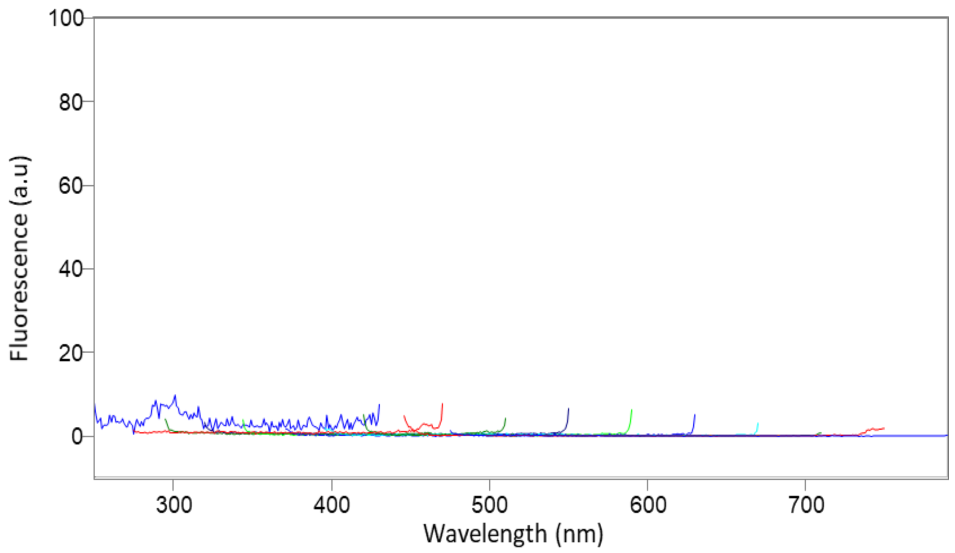

Fig. S2. Fluorescence emission spectra of MilliQ water. Excitation from 220 nm to 400 nm (Δλ between different spectra = 20 nm).

HCl absorption and fluorescence spectra

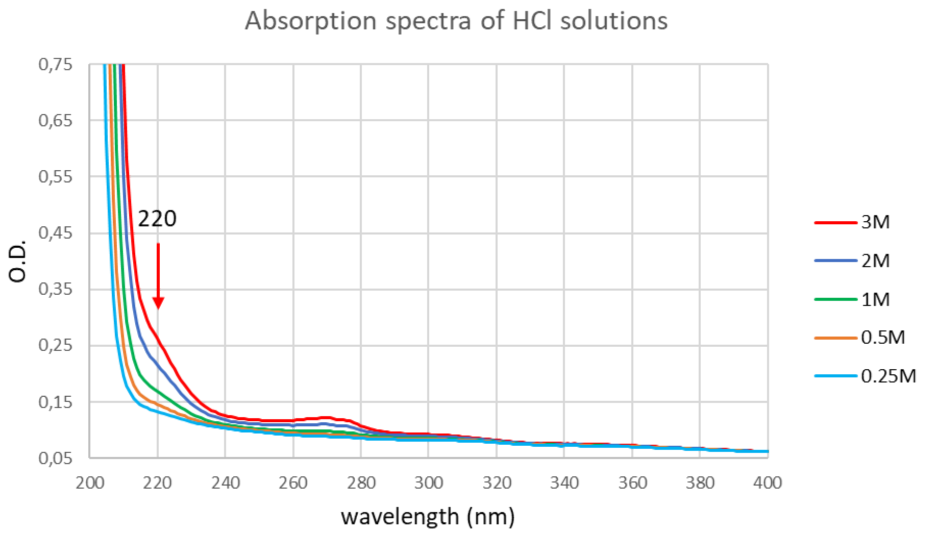

Figure S3. Absorption spectra of HCl aqueous solutions at different concentrations from 0.25M to 3M. The red arrow indicates the excitation wavelength used in fluorescence measurements.

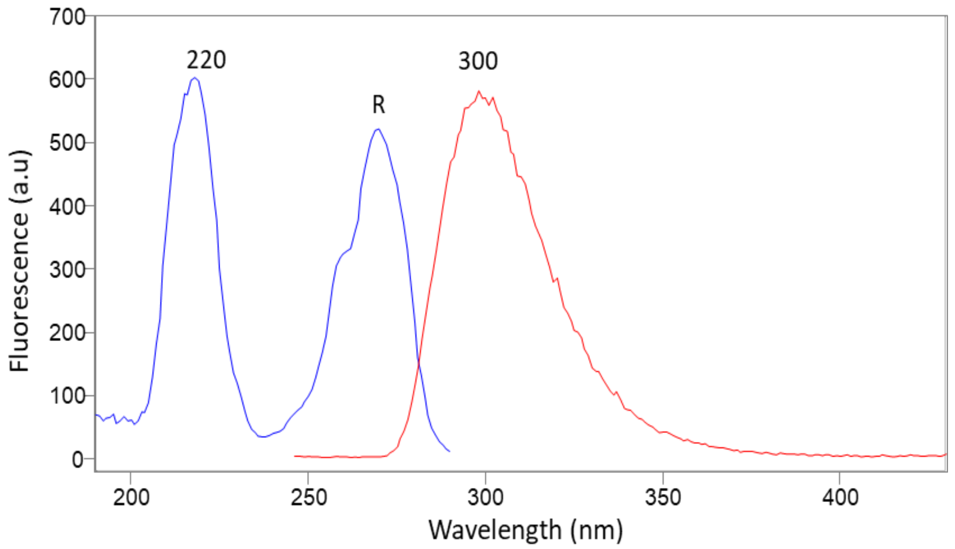

Figure S4. Excitation and emission spectra of 3M HCl solution. Excitation spectrum with emission at 300 nm (blue) with the Raman scattering peak of water (R) (1); emission spectrum with excitation at 220 nm (red). Conditions: spectrofluorometer, Varian Cary Eclipse; photomultiplier gain, 800 V; excitation slit, 5 nm; emission slit, 5 nm.

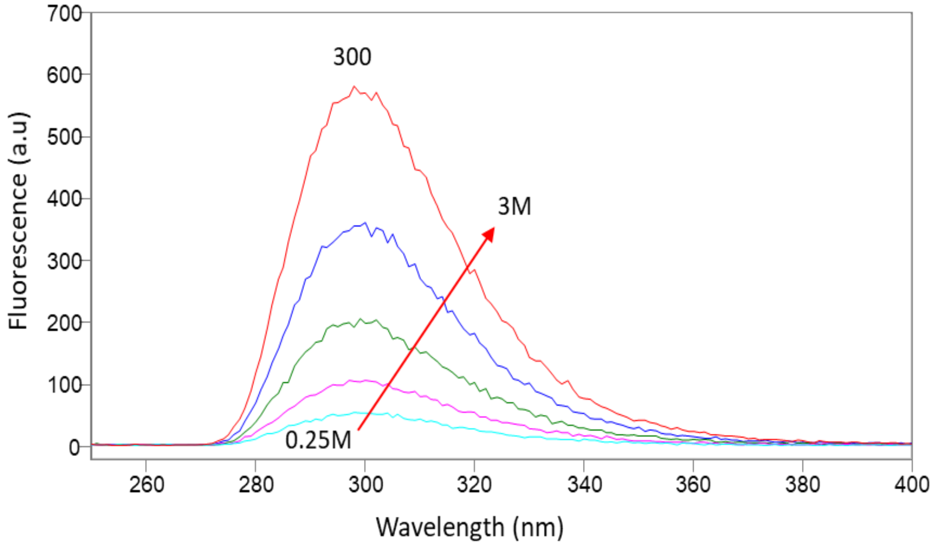

Figure S5. Fluorescence emission spectra of HCl in water at different concentrations from 0.25 to 3M (excitation at 220 nm). Conditions: spectrofluorometer, Varian Cary Eclipse; photomultiplier gain, 800 V; excitation slit, 5 nm; emission slit, 5 nm.

NaOH absorption and fluorescence spectra

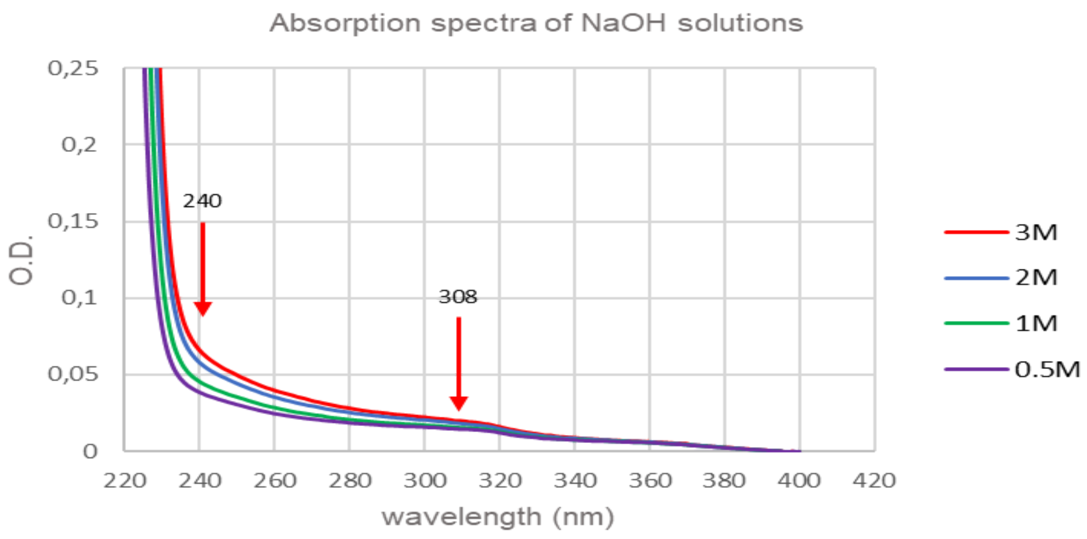

Figure S6. Absorption spectra of NaOH aqueous solutions at different concentrations from 0.5M to 3M. The red arrows indicate the excitation wavelengths used in fluorescence measurements.

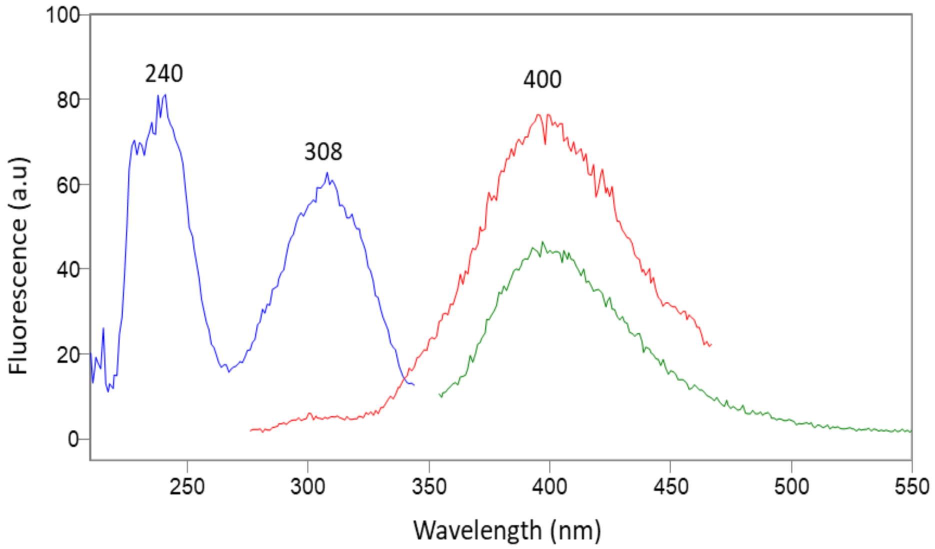

Figure S7. Excitation and emission spectra of 3M NaOH solution. Excitation spectrum with emission at 400 nm (blue); emission spectrum with excitation at 240 nm (red); emission spectrum with excitation at 308 nm (green). Conditions: spectrofluorometer, Varian Cary Eclipse; photomultiplier gain, 900 V; excitation slit, 5 nm; emission slit, 5 nm.

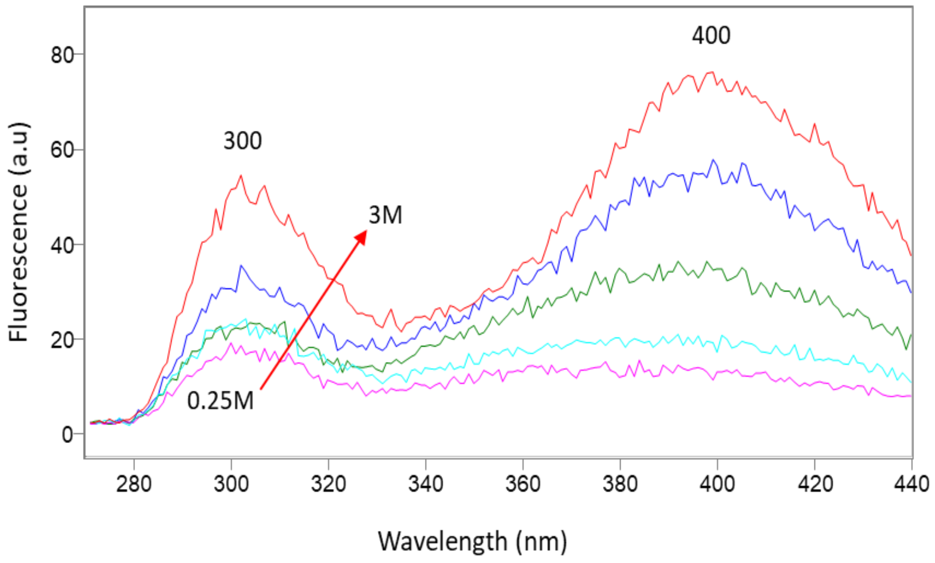

Figure S8. Emission spectra of NaOH solutions at different concentrations from 0.25 to 3M (excitation at 227 nm). Conditions: spectrofluorometer, Varian Cary Eclipse; photomultiplier gain, 900 V; excitation slit, 5 nm; emission slit, 5 nm.

KOH absorption and fluorescence spectra

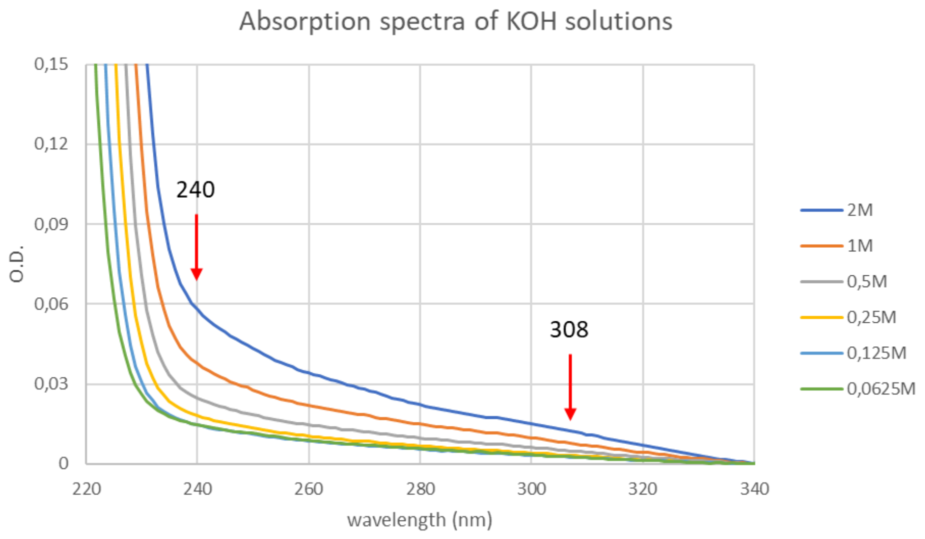

Figure S9. Absorption spectra of KOH aqueous solutions at different concentrations from 0.0625M to 2M. The red arrows indicate the excitation wavelength used in fluorescence measurements.

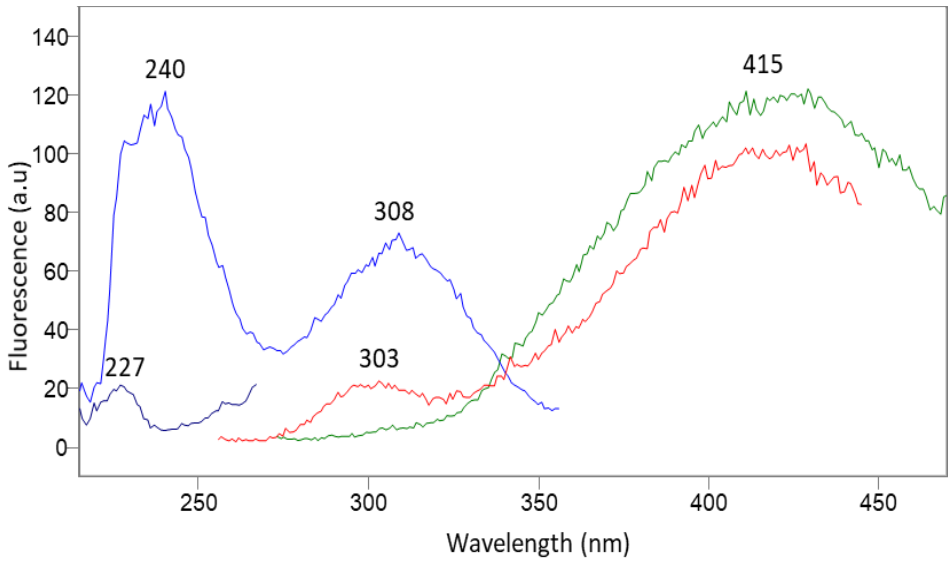

Figure S10. Excitation and emission spectra of 3M KOH solution. Excitation spectrum with emission at 303 nm (blue); excitation spectrum with emission at 415 nm (light blue); emission spectrum with excitation at 227 nm (red); emission spectrum with excitation at 240 nm (green). Conditions: spectrofluorometer, Varian Cary Eclipse; photomultiplier gain, 900 V; excitation slit, 5 nm; emission slit, 5 nm.

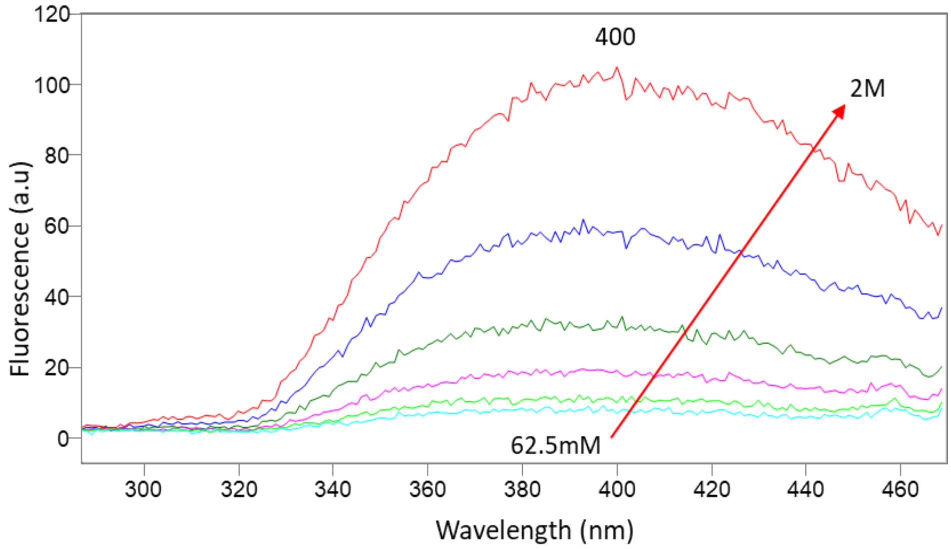

Figure S11. Fluorescence emission spectra of KOH in water at different concentrations from 0.0625 to 2M (excitation at 240 nm). Conditions: spectrofluorometer, Varian Cary Eclipse; photomultiplier gain, 900 V; excitation slit, 5 nm; emission slit, 5 nm.

KCl absorption and fluorescence spectra

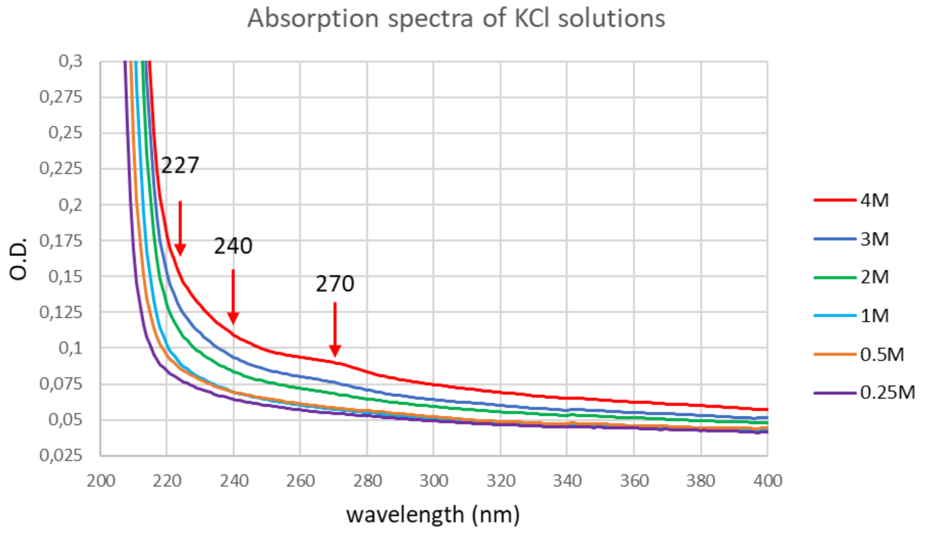

Figure S12. Absorption spectra of KCl aqueous solutions at different concentrations from 0.25M to 4M. The red arrows indicate the excitation wavelength used in fluorescence measurements

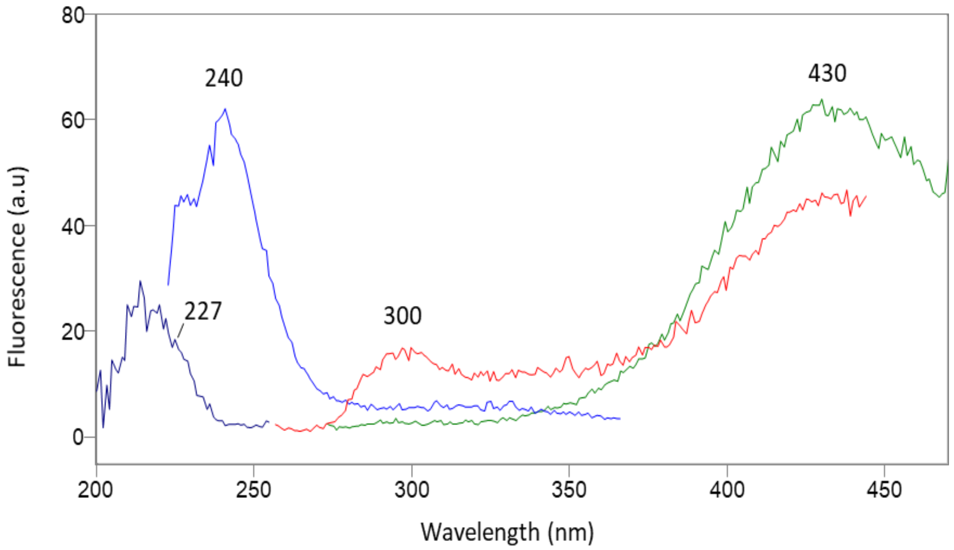

.

Figure S13. Excitation and emission spectra of 4M KCl solution. Excitation spectrum with emission at 295 nm (blue); excitation spectrum with emission at 430 nm (light blue); emission spectrum with excitation at 227 nm (red); emission spectrum with excitation at 240 nm (green). Conditions: spectrofluorometer, Varian Cary Eclipse; photomultiplier gain, 900 V; excitation slit, 5 nm; emission slit, 5 nm.

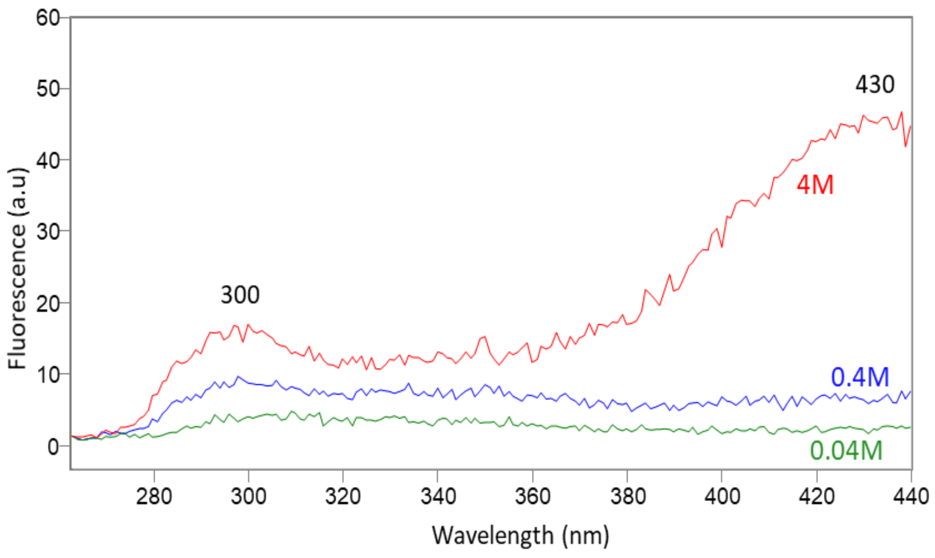

Figure S14. Fluorescence emission spectra of KCl in water at different concentrations from 0.04 to 4 M (excitation at 227 nm). Conditions: spectrofluorometer, Varian Cary Eclipse; photomultiplier gain, 900 V; excitation slit, 5 nm; emission slit, 5 nm.

$CaCl_2$ absorption and fluorescence spectra

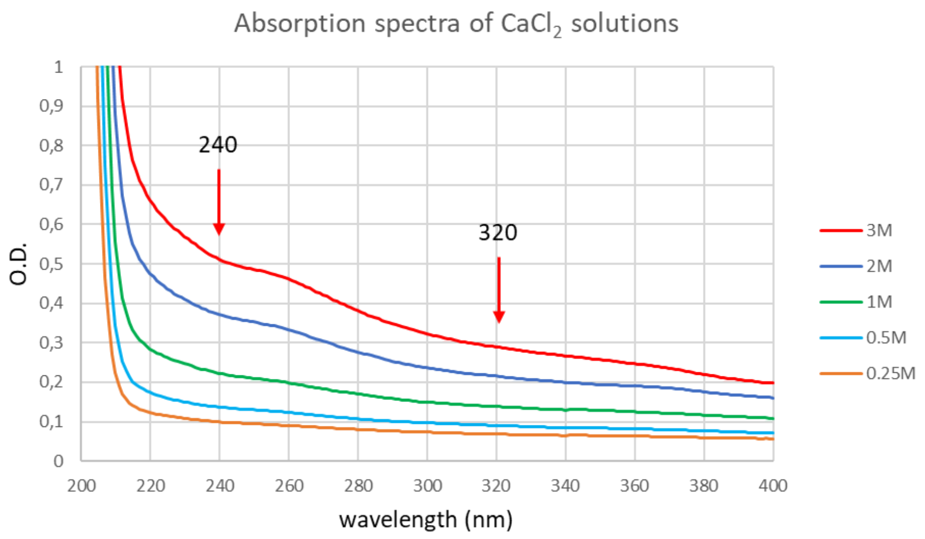

Figure S15. Absorption spectra of $CaCl_2$ solutions. The red arrows indicate the excitation wavelength used in fluorescence measurements.

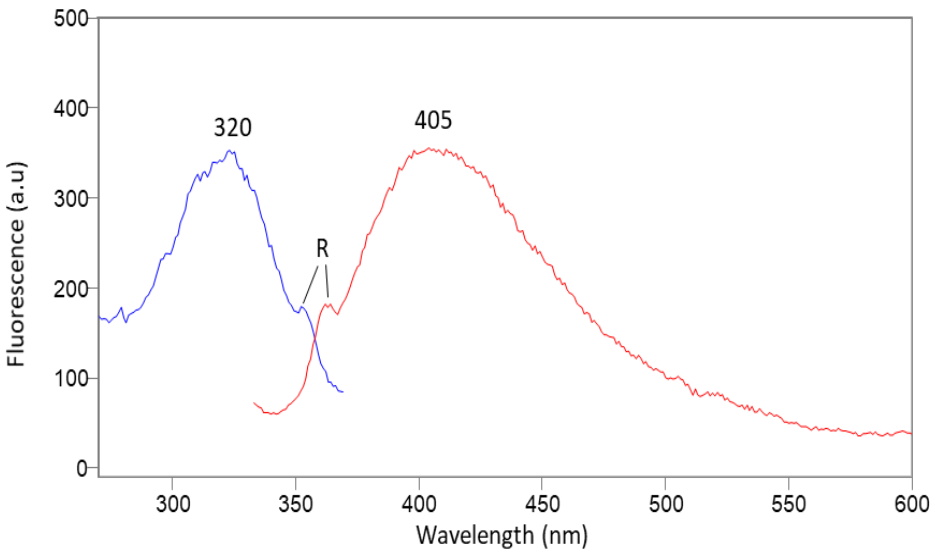

Figure S16. Fluorescence spectra of 3M $CaCl_2$ solution. Excitation spectrum with emission at 405 nm (light blue); emission spectrum with excitation at 320 nm (red). R: Raman band of water. Conditions: spectrofluorometer, Varian Cary Eclipse; photomultiplier gain, 900 V; excitation slit, 5 nm; emission slit, 5 nm.

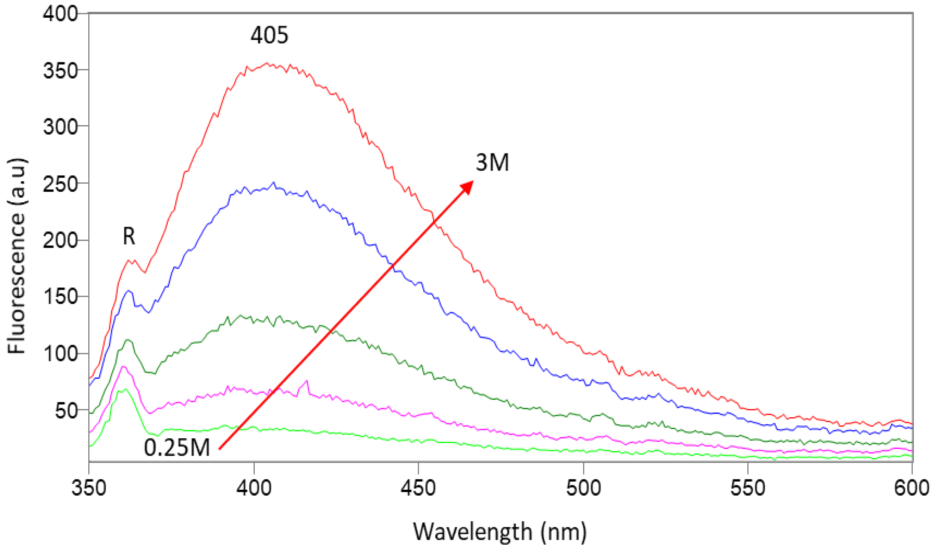

Figure S17. Fluorescence spectra of $CaCl_2$ solutions (3M, 2M, 1M, 0.5M, 0.25M); exc=320 nm. R: Raman band of water. Conditions: spectrofluorometer, Varian Cary Eclipse; photomultiplier gain, 900 V; excitation slit, 5 nm; emission slit, 5 nm.

$MgCl_2$ absorption and fluorescence spectra

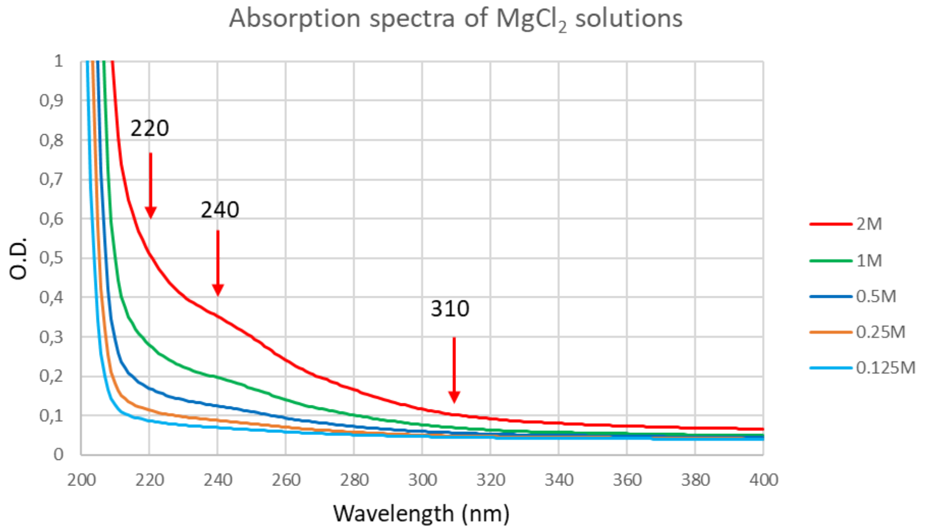

Figure S18. Absorption spectra of $MgCl_2$ solutions.

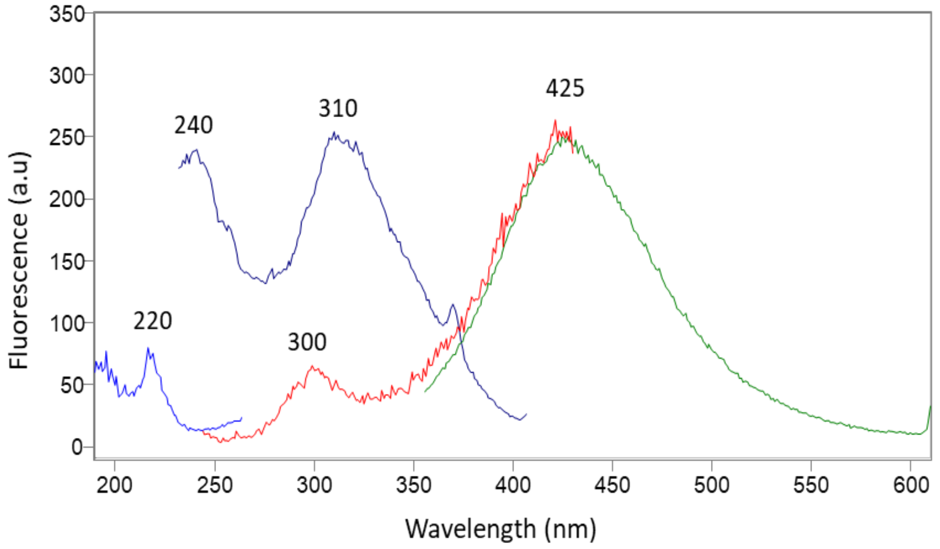

Figure S19. Fluorescence emission spectra of 3M $MgCl_2$ solutions. Excitation spectrum with emission at 300 nm (light blue); excitation spectrum with emission at 425 nm (blue); emission spectra with excitation at 220 nm and 310 nm (red and green, respectively). Conditions: spectrofluorometer, Varian Cary Eclipse; photomultiplier gain, 900 V; excitation slit, 5 nm; emission slit, 5 nm.

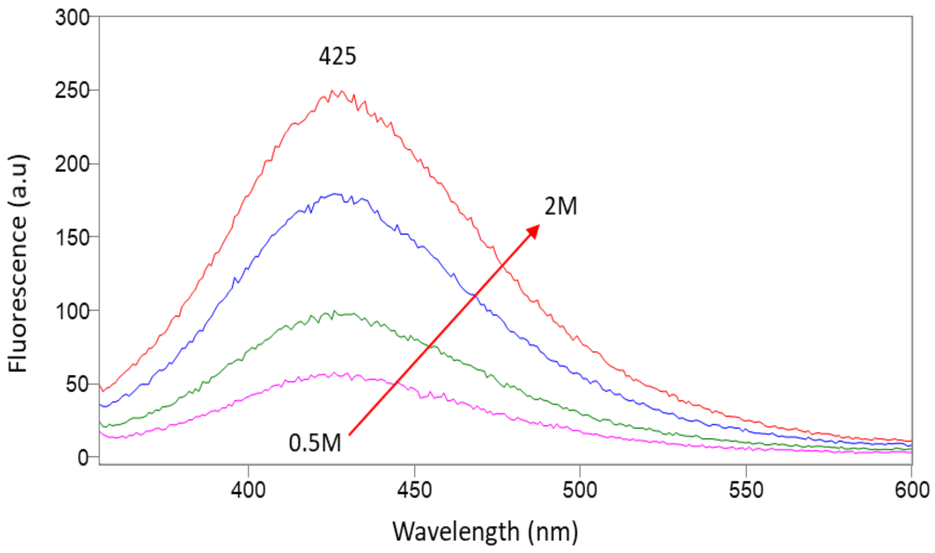

Figure S20. Fluorescence emission spectra of $MgCl_2$ solutions (3M, 2M, 1M, 0.5M); exc=310 nm. Conditions: spectrofluorometer, Varian Cary Eclipse; photomultiplier gain, 900 V; excitation slit, 5 nm; emission slit, 5 nm.

$MgSO_4$ absorption and fluorescence spectra

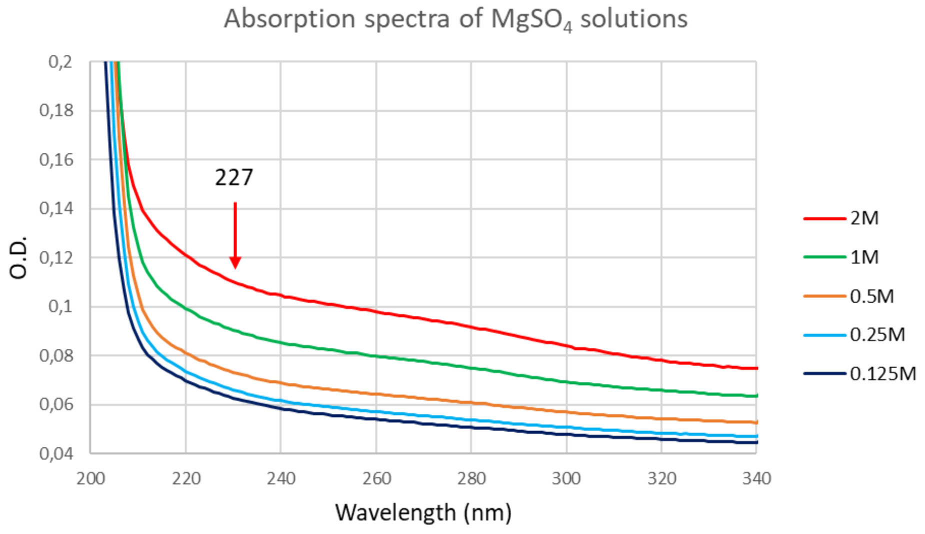

Figure S21. Absorption spectra of $MgSO_4$ solutions.

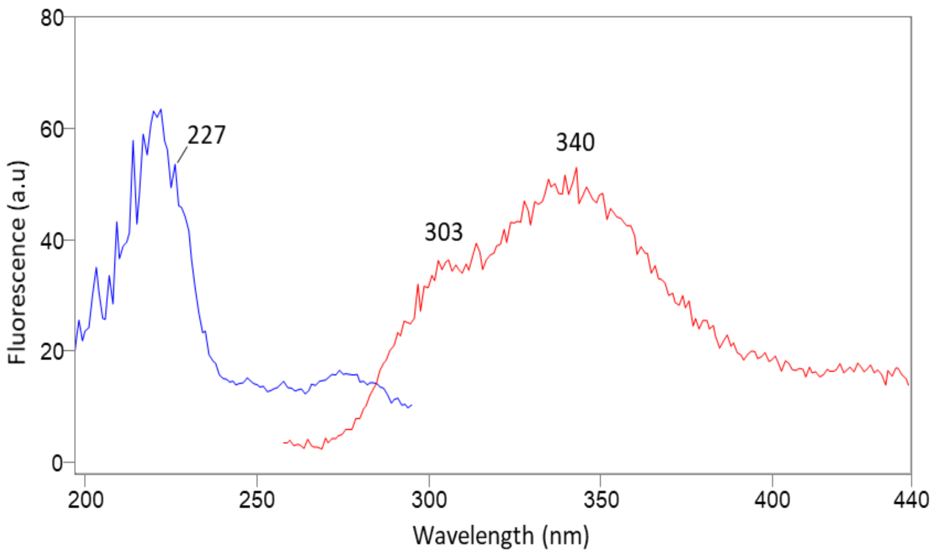

Figure S22. Fluorescence spectra of 2M $MgSO_4$ solution. Excitation spectrum with emission at 340 nm (light blue); emission spectrum with excitation at 227 nm (red). Conditions: spectrofluorometer, Varian Cary Eclipse; photomultiplier gain, 900 V; excitation slit, 5 nm; emission slit, 5 nm.

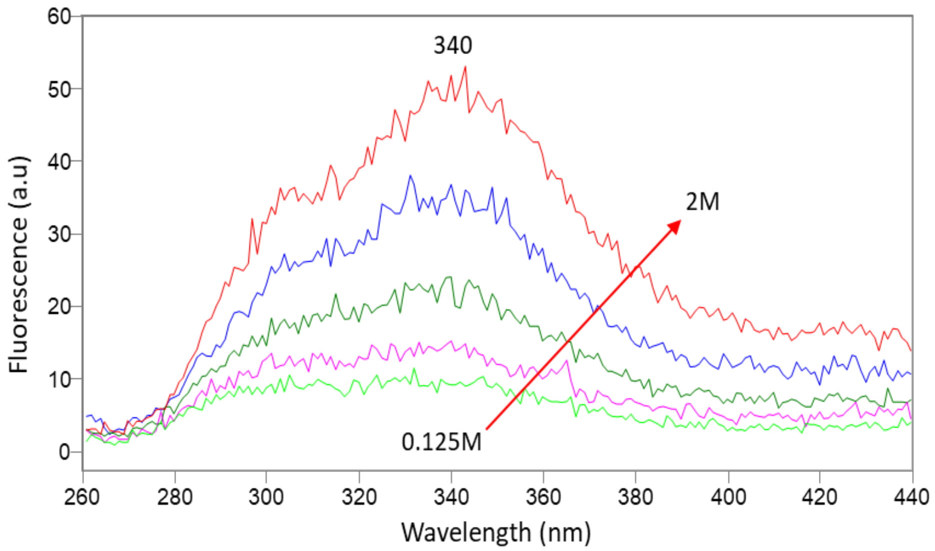

Figure S23. Fluorescence emission spectra of $MgSO_4$ solutions (2M, 1M, 0.5M, 0.25M, 0.125M); exc=227 nm. Conditions: spectrofluorometer, Varian Cary Eclipse; photomultiplier gain, 900 V; excitation slit, 5 nm; emission slit, 5 nm.

K_2SO_4 absorption and fluorescence spectra

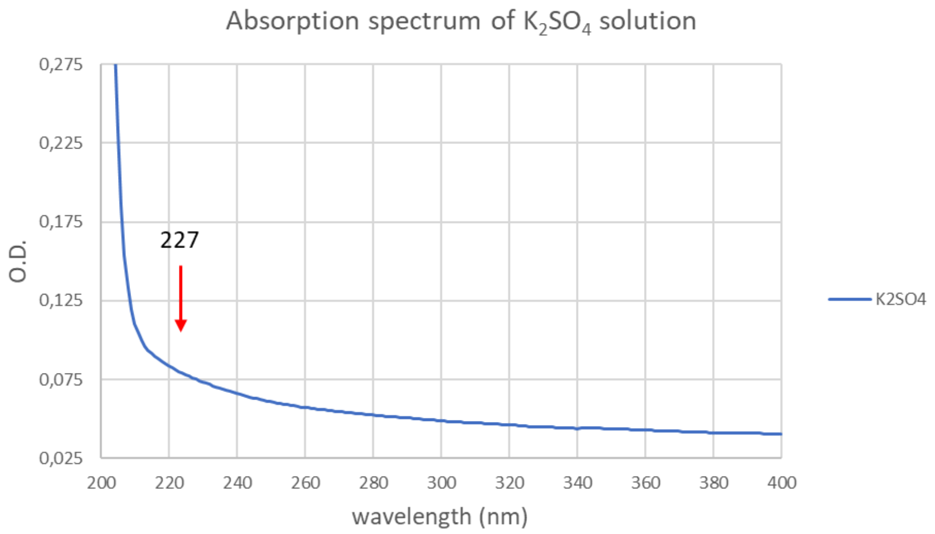

Figure S24. Absorption spectrum of 0.5M K_2SO_4 solution.

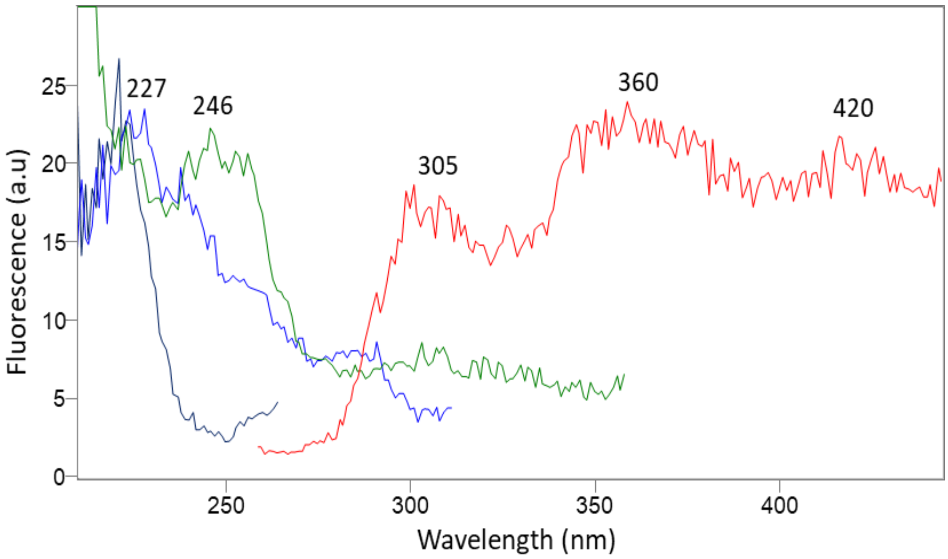

Figure S25. Excitation and emission fluorescence spectra of 0.5M K_2SO_4 solution. Excitation spectrum with emission at 306nm (blue); excitation spectrum with em=360 nm (light blue); excitation spectrum with emission at 420 nm (green); red: emission spectrum with exc= 227 nm. Conditions: spectrofluorometer, Varian Cary Eclipse; photomultiplier gain, 900 V; excitation slit, 5 nm; emission slit, 5 nm.

Na_2SO_4 absorption and fluorescence spectra

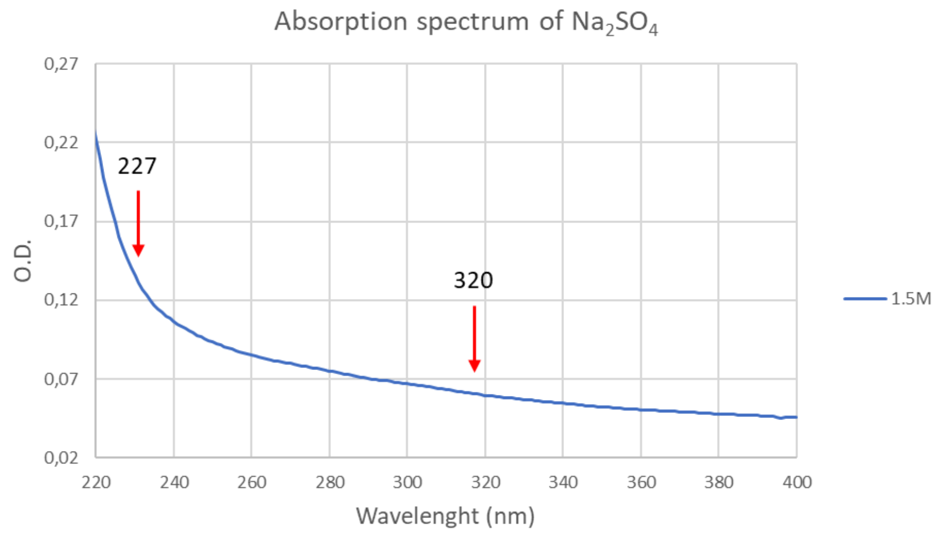

Figure S26. Absorption spectrum of 1.5M Na_2SO_4 solution.

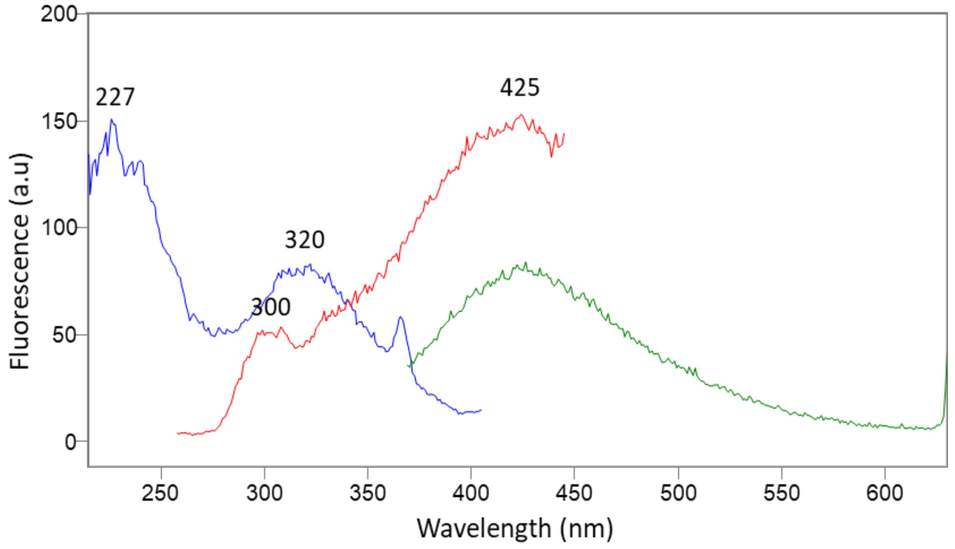

Figure S27. Excitation and emission fluorescence spectra of 1.5M Na_2SO_4 solutions; red: emission with exc=227 nm; green: emission with exc=320 nm; blue: excitation with em=420 nm. Conditions: spectrofluorometer, Varian Cary Eclipse; photomultiplier gain, 900 V; excitation slit, 5 nm; emission slit, 5 nm.

$(NH_4)_2SO_4$ absorption and fluorescence spectra

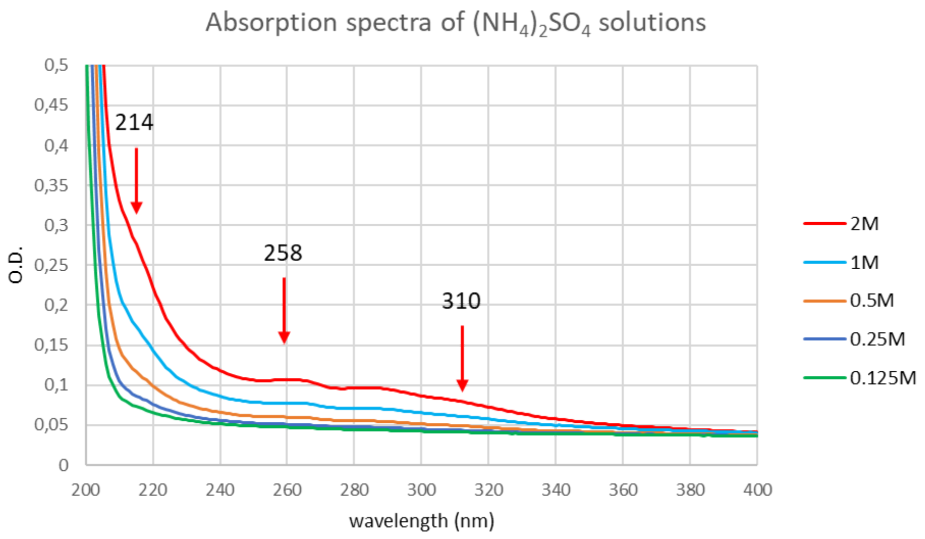

Figure S28. Absorption spectra of $(NH_4)_2SO_4$ solutions.

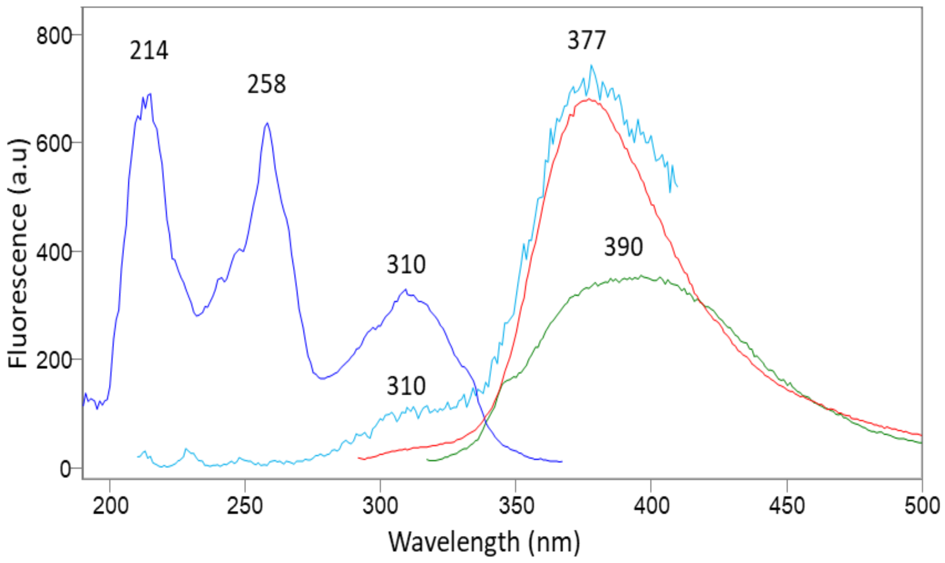

Figure S29. Fluorescence spectra of 2M $(NH_4)_2SO_4$ solution. Excitation spectrum with emission at 377 nm (blue); emission spectrum with excitation at 210 nm (light blue); emission spectrum with excitation at 258 nm (red); emission spectrum with excitation at 308 nm (green). Conditions: spectrofluorometer, Varian Cary Eclipse; photomultiplier gain, 900 V; excitation slit, 5 nm; emission slit, 5 nm.

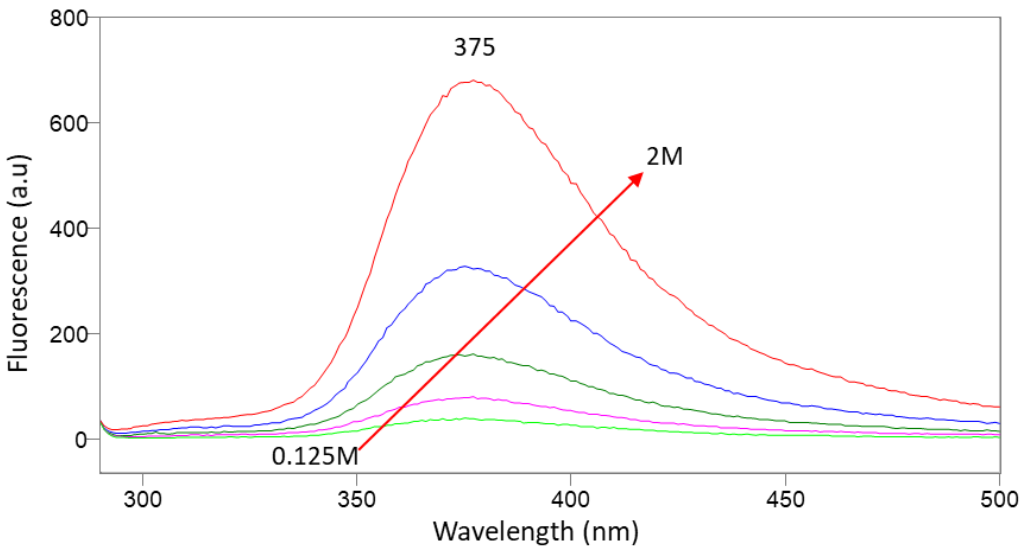

Figure S30. Fluorescence emission spectra of $(NH_4)_2SO_4$ solutions (2M, 1M, 0.5M, 0.25M, 0.125M); exc=258nm. Conditions: spectrofluorometer, Varian Cary Eclipse; photomultiplier gain, 900 V; excitation slit, 5 nm; emission slit, 5 nm.

Lysine absorption and fluorescence spectra

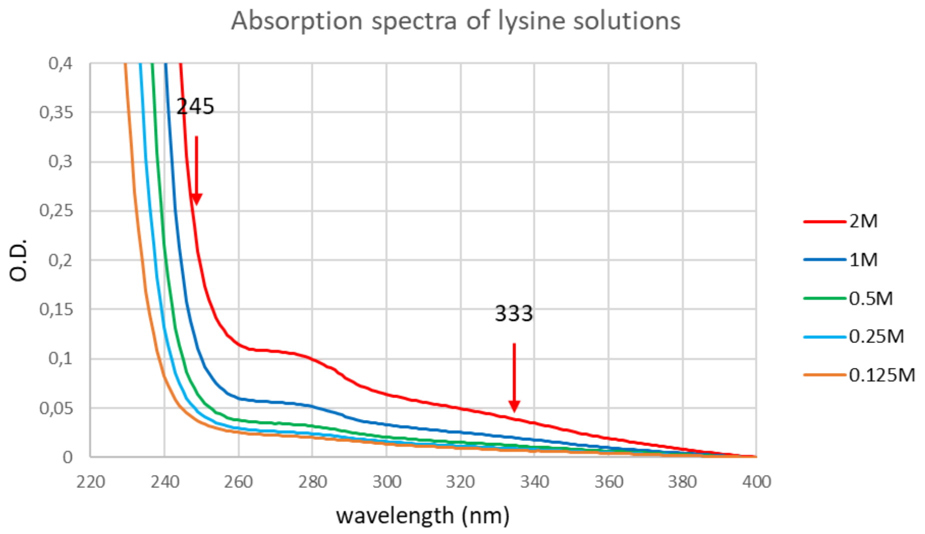

Figure S31. Absorption spectra of lysine solutions.

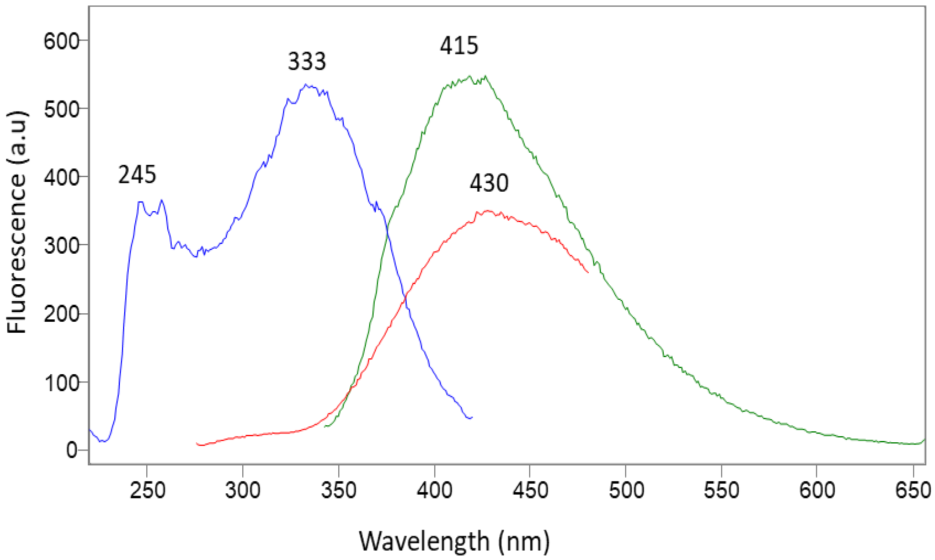

Figure S32. Fluorescence spectra of 2M lysine solution. Excitation spectrum with emission at 430 nm (light blue); emission spectrum with excitation at 245 nm (red); emission spectrum with excitation at 333 nm (green). Conditions: spectrofluorometer, Varian Cary Eclipse; photomultiplier gain, 900 V; excitation slit, 5 nm; emission slit, 5 nm.

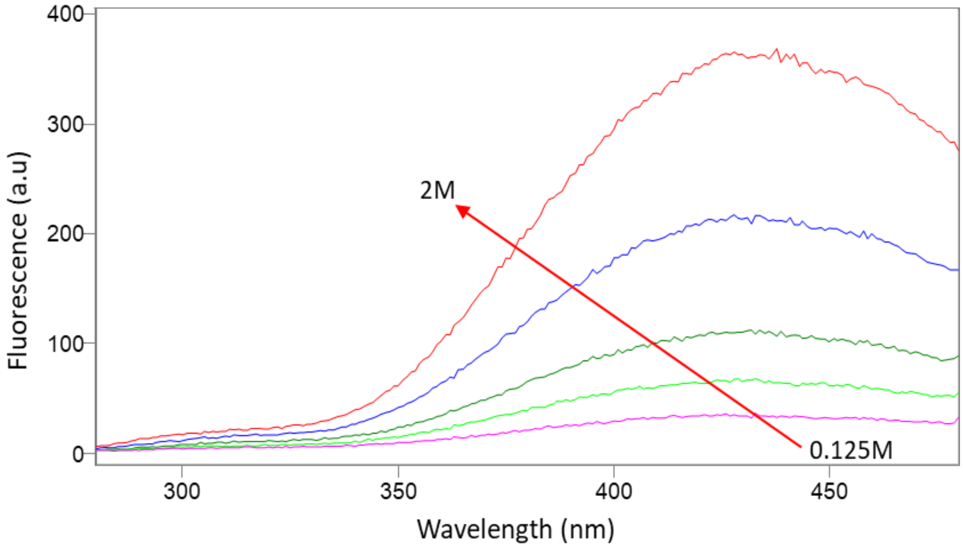

Figure S33. Fluorescence emission spectra of lysine solutions (2M, 1M, 0.5M, 0.25M, 0.125M); exc=245 nm. Conditions: spectrofluorometer, Varian Cary Eclipse; photomultiplier gain, 900 V; excitation slit, 5 nm; emission slit, 5 nm.

Glycine absorption and fluorescence spectra

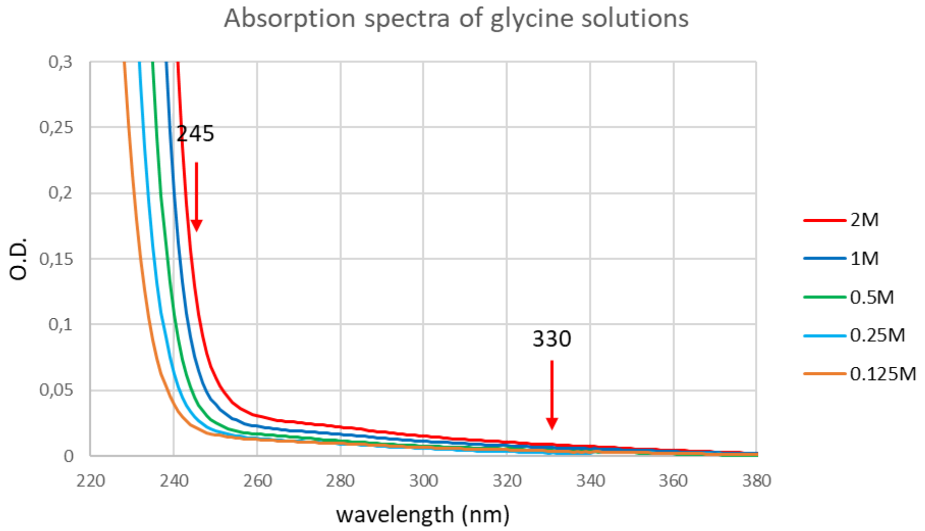

Figure S34. Absorption spectra of glycine solutions

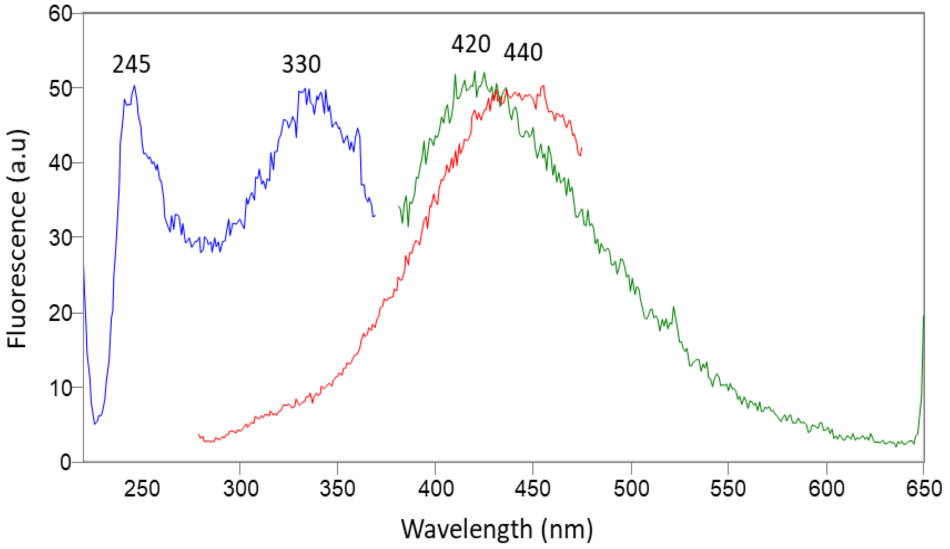

Figure S35. Fluorescence spectra of 2M glycine solution. Excitation spectrum with emission at 440 nm (light blue); emission spectrum with excitation at 245 nm (red); emission spectrum with excitation at 330 nm (green). Conditions: spectrofluorometer, Varian Cary Eclipse; photomultiplier gain, 900 V; excitation slit, 5 nm; emission slit, 5 nm.

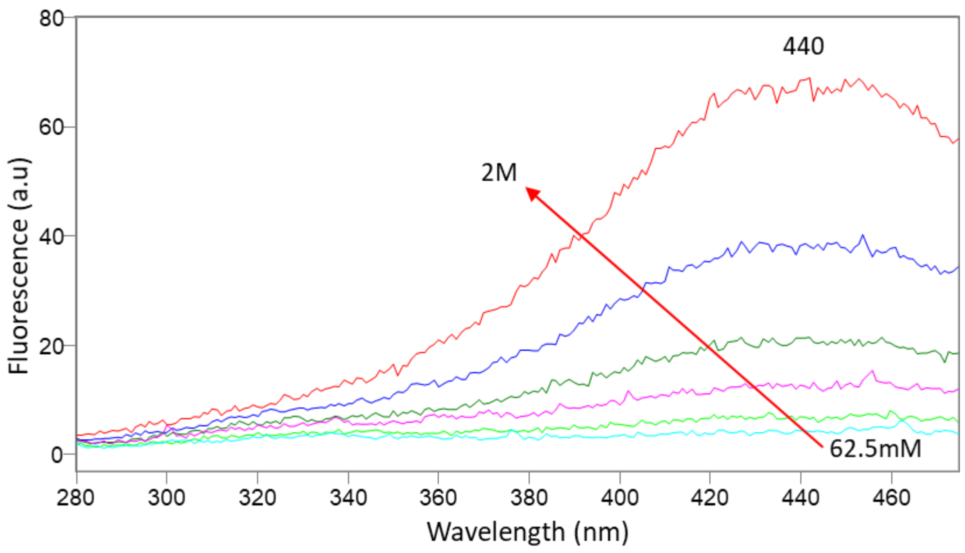

Figure S36. Fluorescence emission spectra of glycine solutions (2M, 1M, 0.5M, 0.25M, 0.125M, 0.0625M); exc=245 nm. Conditions: spectrofluorometer, Varian Cary Eclipse; photomultiplier gain, 900 V; excitation slit, 5 nm; emission slit, 5 nm.

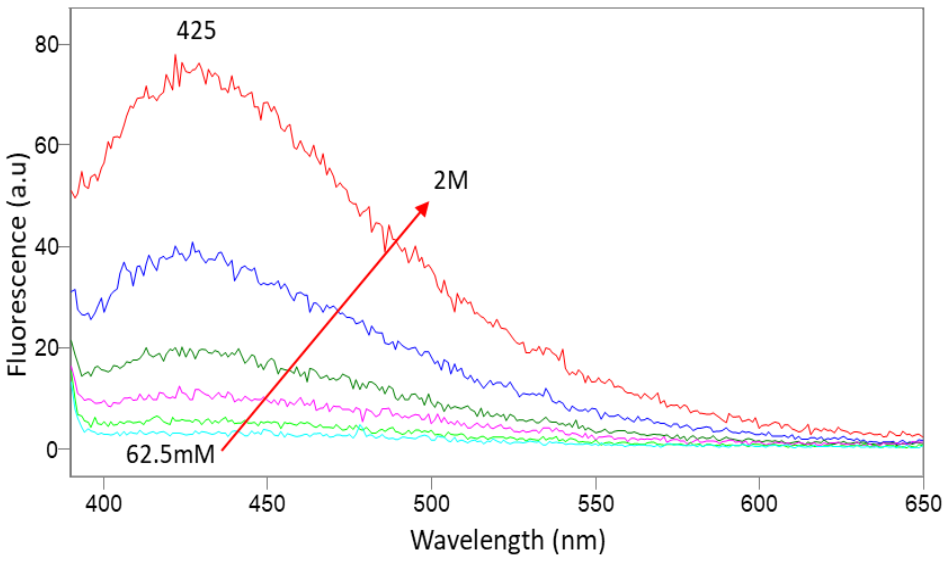

Figure S37. Fluorescence emission spectra of glycine solutions (2M, 1M, 0.5M, 0.25M, 0.125M, 0.0625M); exc=338 nm. Conditions: spectrofluorometer, Varian Cary Eclipse; photomultiplier gain, 900 V; excitation slit, 5 nm; emission slit, 5 nm.

Lactose absorption and fluorescence spectra

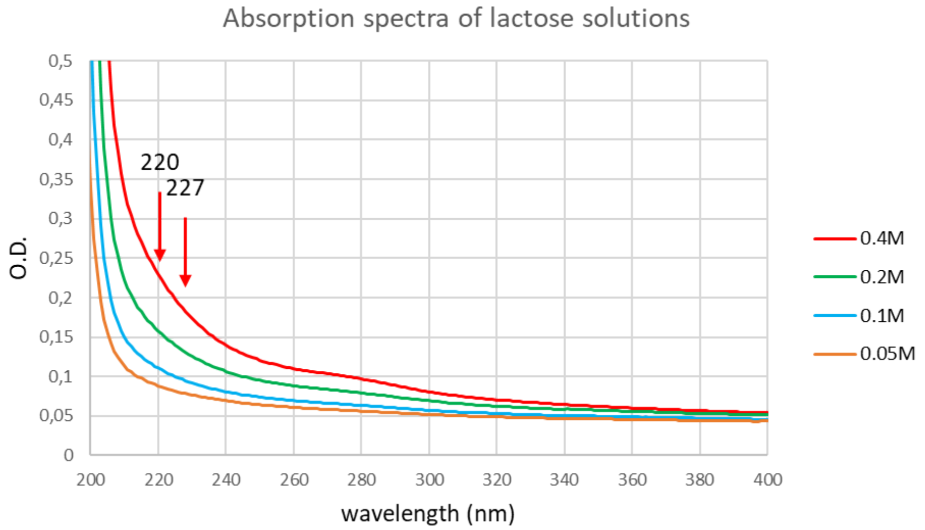

Figure S38. Absorption spectra of lactose solutions.

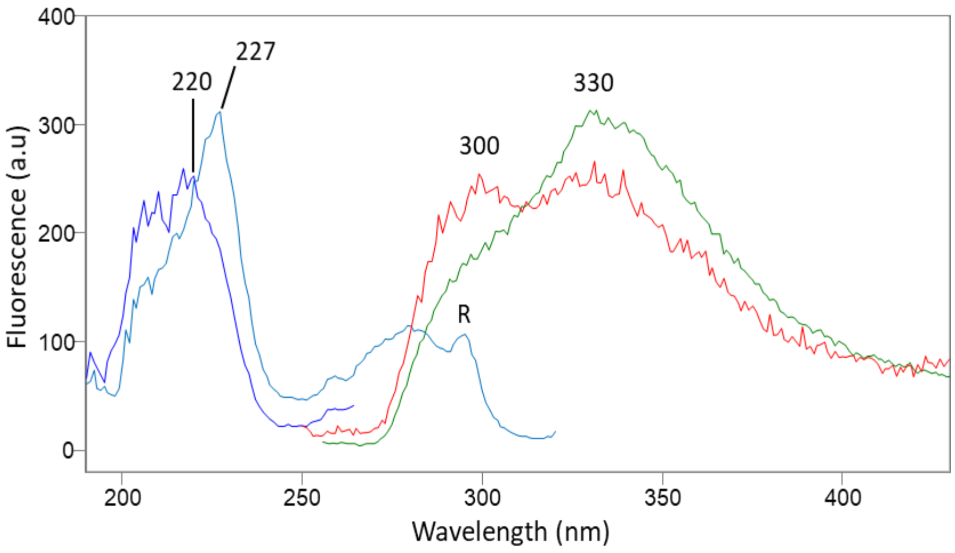

Figure S39. Fluorescence spectra of 0.4M lactose solution. Excitation spectrum with emission at 300 nm (blue); excitation spectrum with emission at 330 nm (light blue); emission spectrum with excitation at 220 nm (red); emission spectrum with excitation at 227 nm (green); R: Raman band of water. Conditions: spectrofluorometer, Varian Cary Eclipse; photomultiplier gain, 900 V; excitation slit, 5 nm; emission slit, 5 nm.

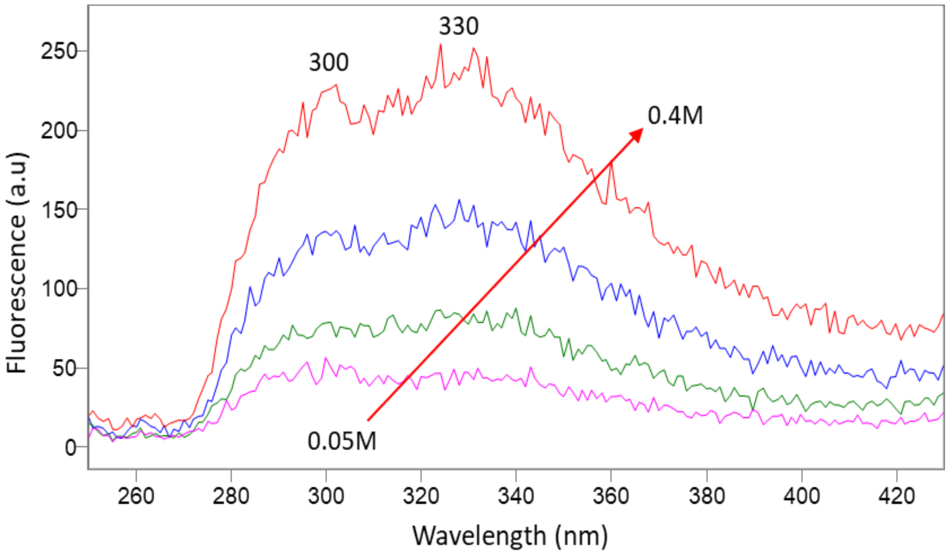

Figure S40. Fluorescence emission spectra of lactose solutions (0.4M, 0.2M, 0.1M, 0.05M); exc=220 nm. Conditions: spectrofluorometer, Varian Cary Eclipse; photomultiplier gain, 900 V; excitation slit, 5 nm; emission slit, 5 nm.

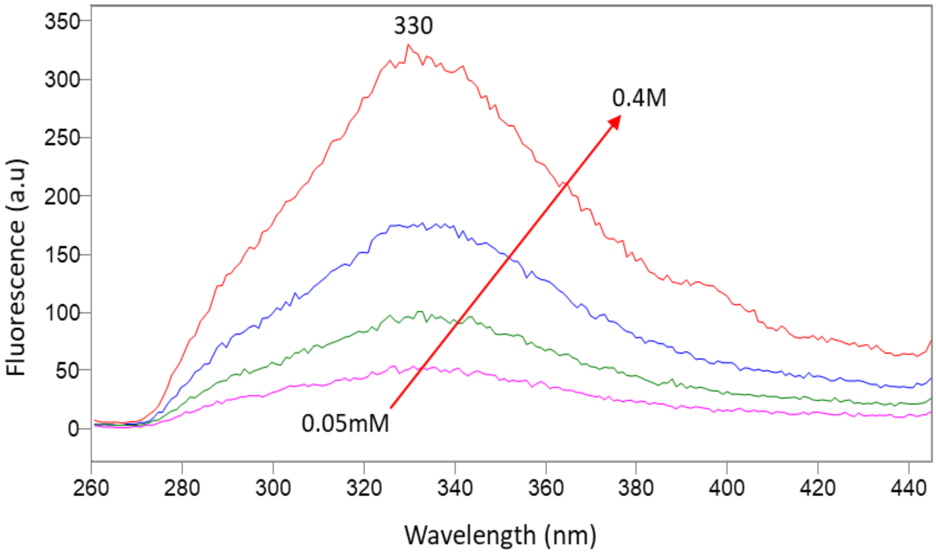

Figure S41. Fluorescence emission spectra of lactose solutions (0.4M, 0.2M, 0.1M, 0.05M); exc=227 nm. Conditions: spectrofluorometer, Varian Cary Eclipse; photomultiplier gain, 900 V; excitation slit, 5 nm; emission slit, 5 nm.

Glucose absorption and fluorescence spectra

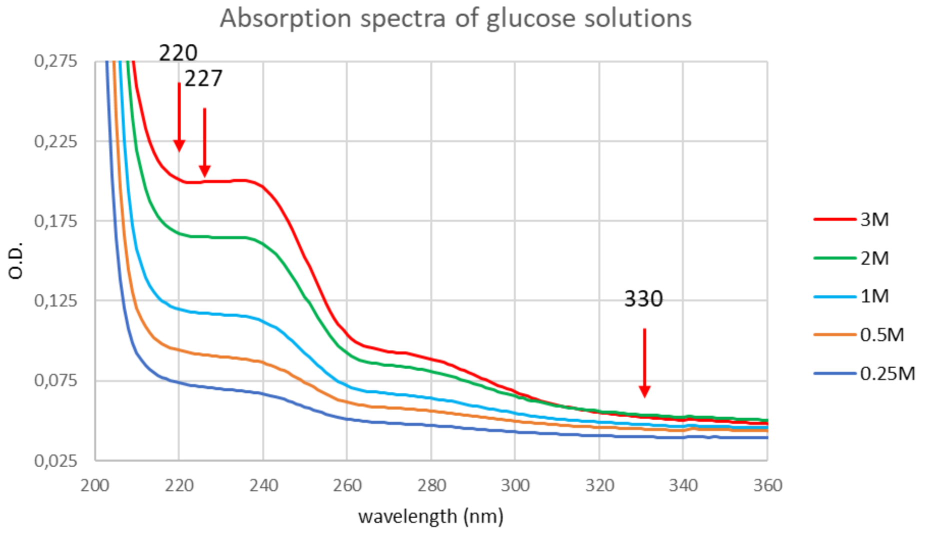

Figure S42. Absorption spectra of glucose solutions.

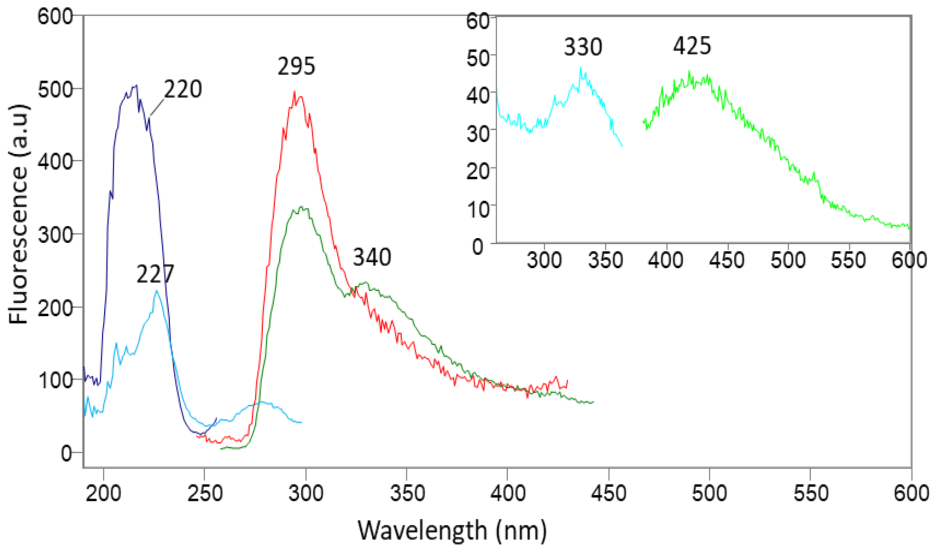

Figure S43. Fluorescence spectra of 3M glucose solution. Excitation spectrum with emission at 295 nm (blue); excitation spectrum with emission at 340 nm (light blue); emission spectrum with excitation at 220 nm (red); emission spectrum with excitation at 227 nm (green). Inset: excitation spectrum with emission at 425 nm (light blue); emission spectrum with excitation at 330 nm (light green). Conditions: spectrofluorometer, Varian Cary Eclipse; photomultiplier gain, 900 V; excitation slit, 5 nm; emission slit, 5 nm.

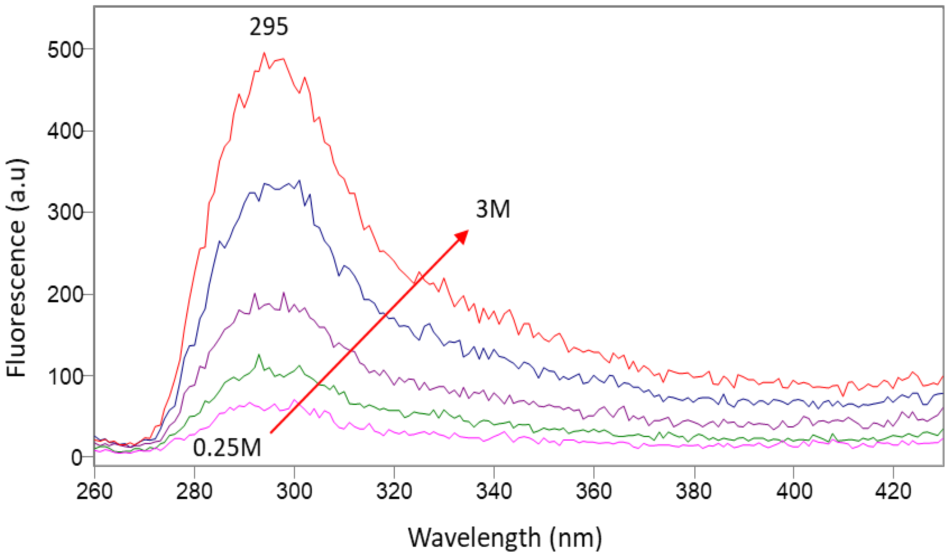

Figure S44. Fluorescence emission spectra of glucose solutions (3M, 2M, 1M, 0.5M; 0.25M); exc=220 nm. Conditions: spectrofluorometer, Varian Cary Eclipse; photomultiplier gain, 900 V; excitation slit, 5 nm; emission slit, 5 nm.

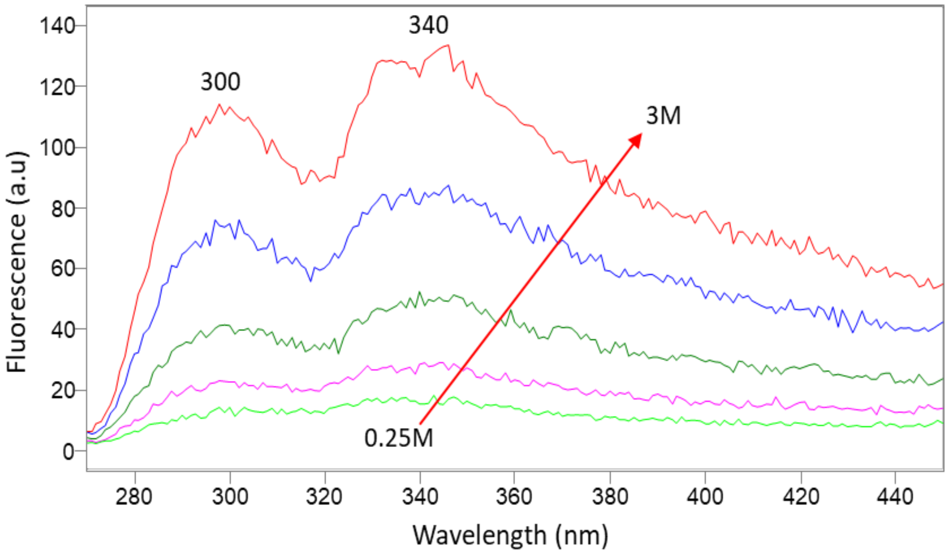

Figure S45. Fluorescence emission spectra of glucose solutions (3M, 2M, 1M, 0.5M; 0.25M); exc=235 nm. Conditions: spectrofluorometer, Varian Cary Eclipse; photomultiplier gain, 900 V; excitation slit, 5 nm; emission slit, 5 nm.

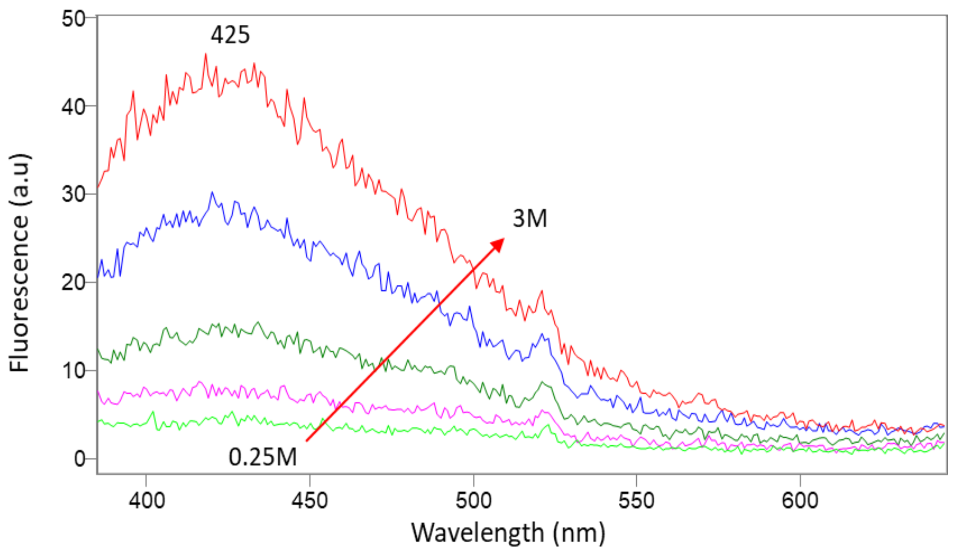

Figure S46. Fluorescence emission spectra of glucose solutions (3M, 2M, 1M, 0.5M; 0.25M); exc=330 nm. Conditions: spectrofluorometer, Varian Cary Eclipse; photomultiplier gain, 900 V; excitation slit, 5 nm; emission slit, 5 nm.

Trehalose absorption and fluorescence spectra

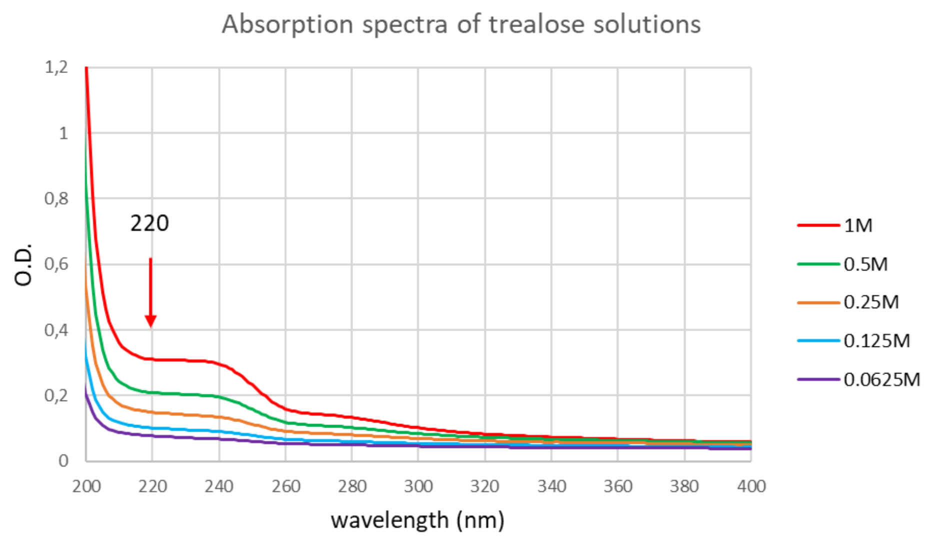

Figure S47. Absorption spectra of trehalose solutions.

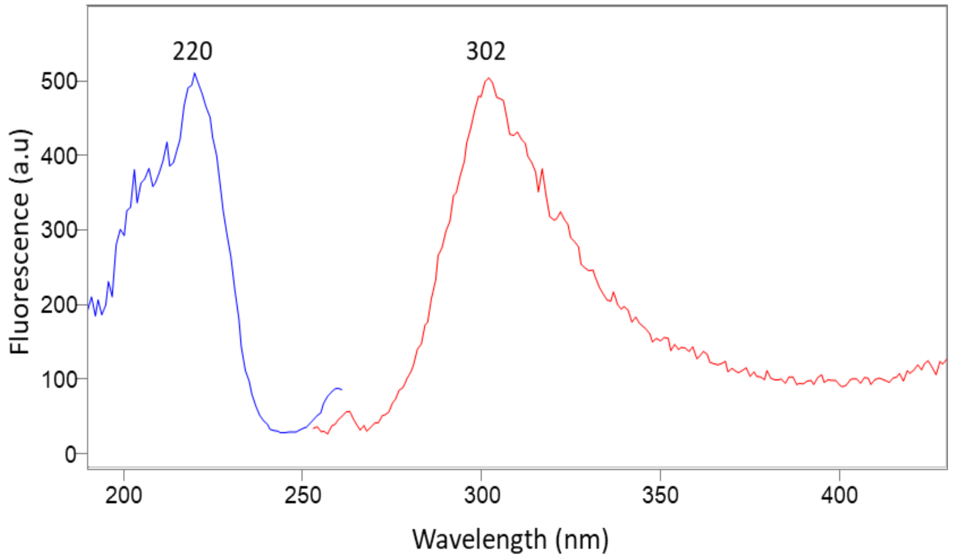

Figure S48. Fluorescence spectra of 1M trehalose solution. Excitation spectrum with emission at 302 nm (blue); emission spectrum with excitation at 220 nm (red). Conditions: spectrofluorometer, Varian Cary Eclipse; photomultiplier gain, 900 V; excitation slit, 5 nm; emission slit, 5 nm.

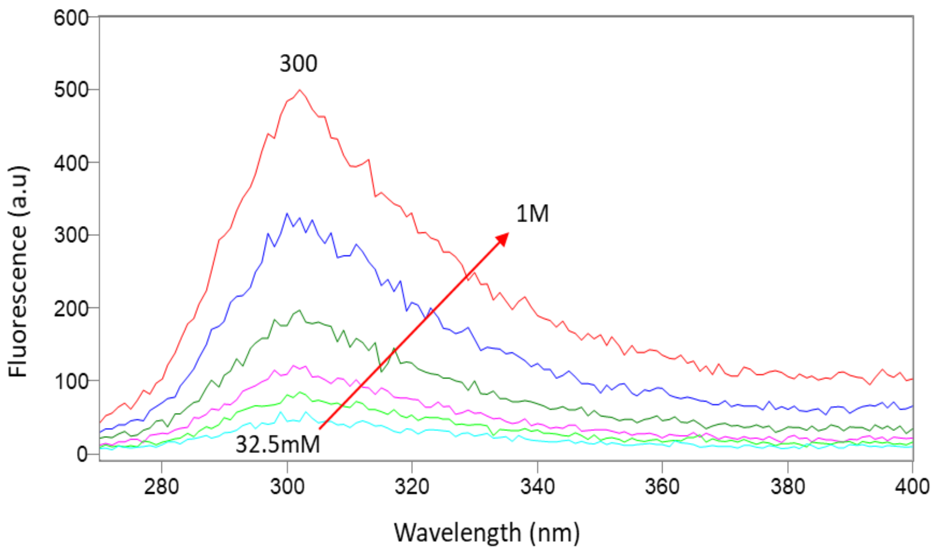

Figure S49. Fluorescence emission spectra of trehalose solutions (1M, 0.5M, 0.25M, 0.125M, 0.0625M, 0.0325M); exc=220 nm. Conditions: spectrofluorometer, Varian Cary Eclipse; photomultiplier gain, 900 V; excitation slit, 5 nm; emission slit, 5 nm.

Xylitol absorption and fluorescence spectra

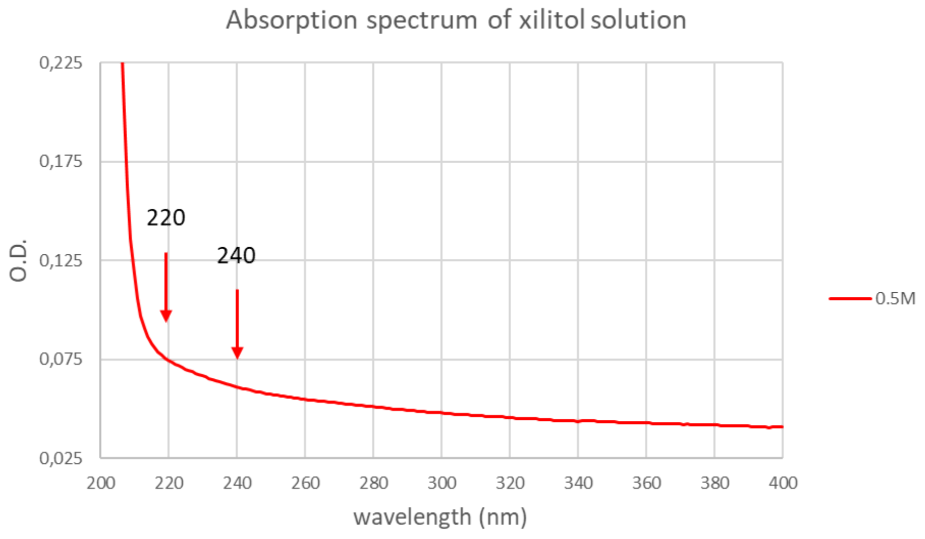

Figure S50. Absorption spectra of 0.5M xylitol solution.

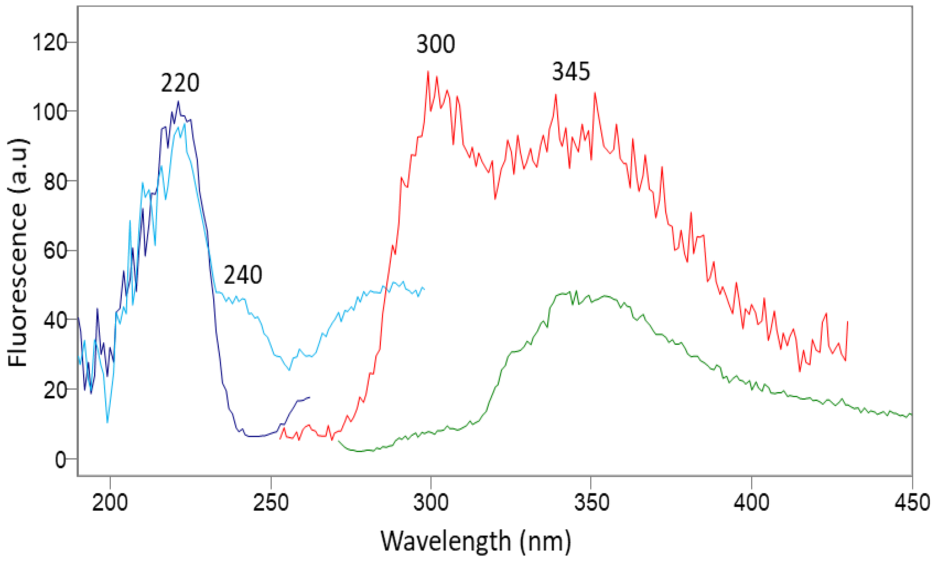

Figure S51. Fluorescence spectra of 0.5M xylitol solution. Excitation spectrum with emission at 300 nm (blue); excitation spectrum with emission at 345 nm (blue); emission spectrum with excitation at 220 nm (red); emission spectrum with excitation at 240 nm (green). Conditions: spectrofluorometer, Varian Cary Eclipse; photomultiplier gain, 900 V; excitation slit, 5 nm; emission slit, 5 nm.

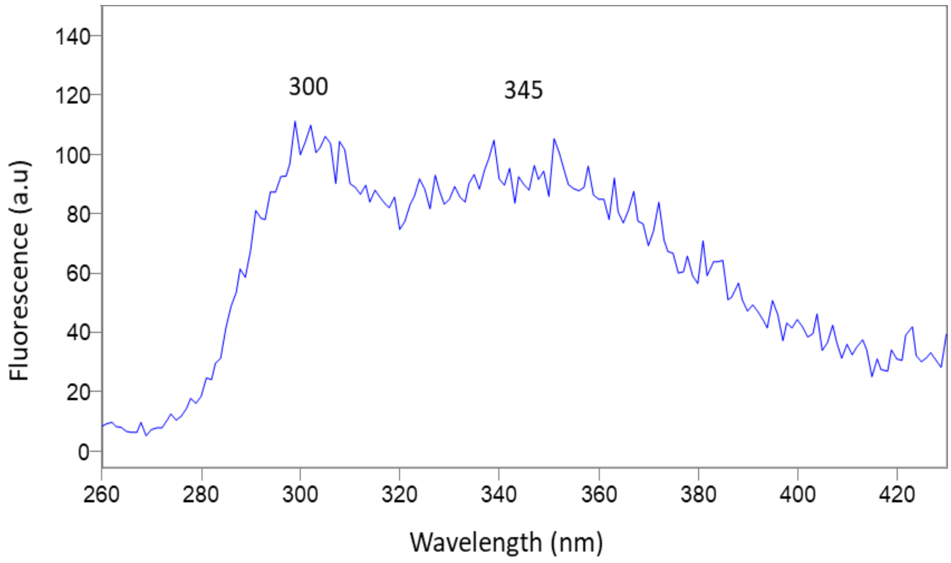

Figure S52. Fluorescence emission spectrum of xylitol 0.5M; exc=220 nm. Conditions: spectrofluorometer, Varian Cary Eclipse; photomultiplier gain, 900 V; excitation slit, 5 nm; emission slit, 5 nm.

Sorbitol absorption and fluorescence spectra

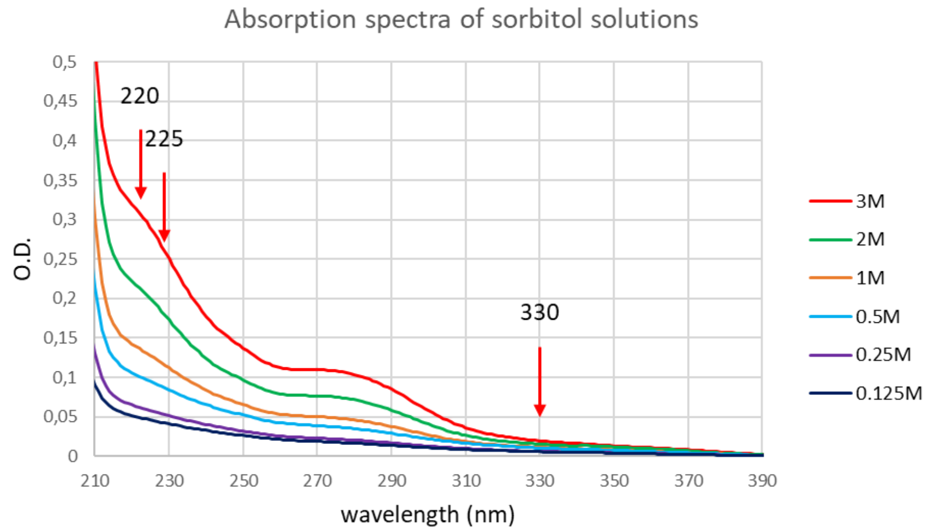

Figure S53. Absorption spectra of sorbitol solutions.

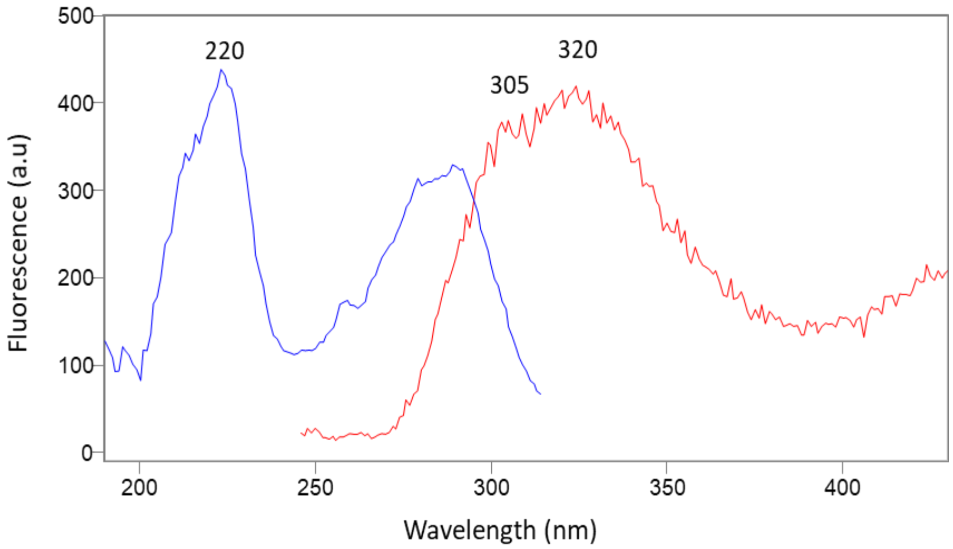

Figure S54. Fluorescence spectra of 3M sorbitol solution. Excitation spectrum with emission at 324 nm (blue); emission spectrum with excitation at 220 nm (red). Conditions: spectrofluorometer, Varian Cary Eclipse; photomultiplier gain, 900 V; excitation slit, 5 nm; emission slit, 5 nm.

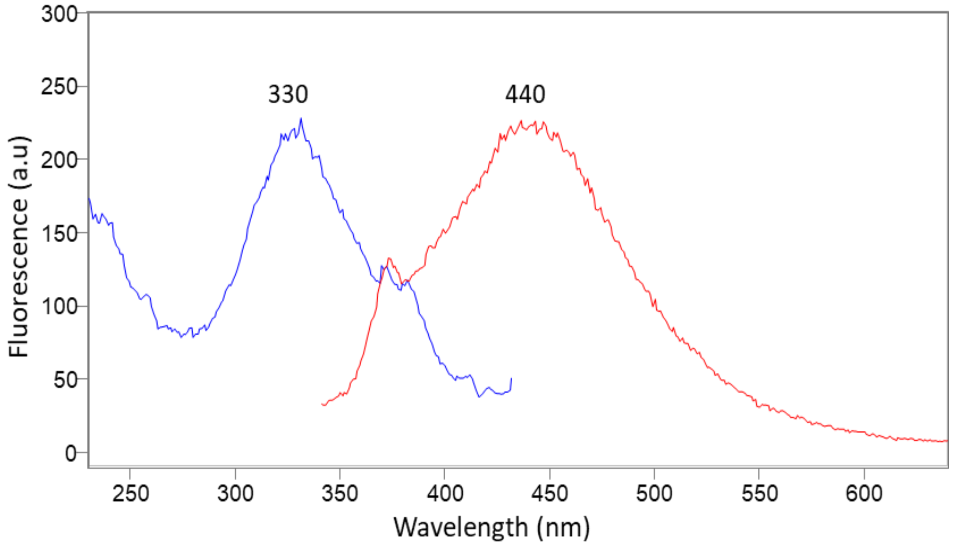

Figure S55. Fluorescence spectra of 3M sorbitol solution. Excitation spectrum with emission at 440 nm (blue); emission spectrum with excitation at 330 nm (red). Conditions: spectrofluorometer, Varian Cary Eclipse; photomultiplier gain, 900 V; excitation slit, 5 nm; emission slit, 5 nm.

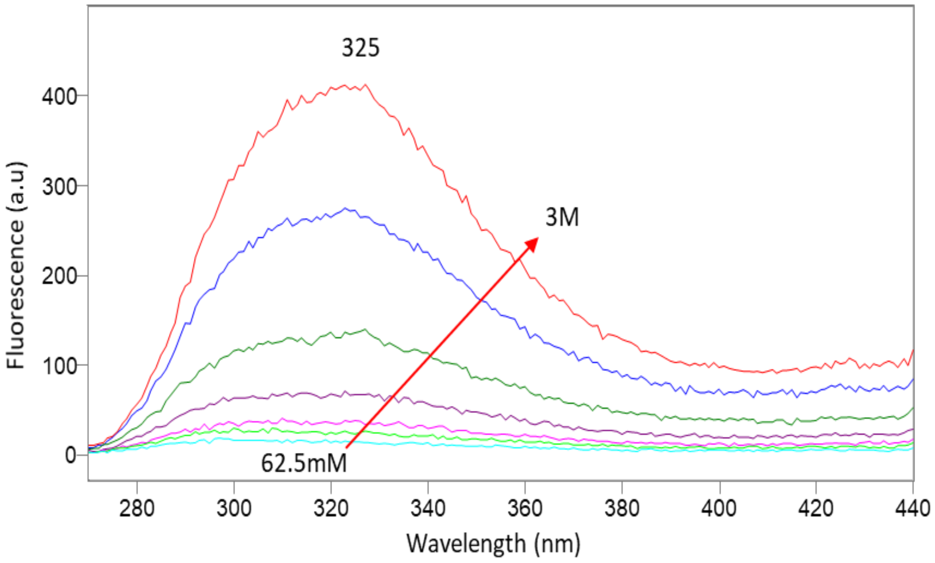

Figure S56. Fluorescence emission spectra of sorbitol solutions (3M, 2M, 1M, 0.5M, 0.25M, 0.125M, 0.0625M); exc=225 nm. Conditions: spectrofluorometer, Varian Cary Eclipse; photomultiplier gain, 900 V; excitation slit, 5 nm; emission slit, 5 nm.

Urea absorption and fluorescence spectra

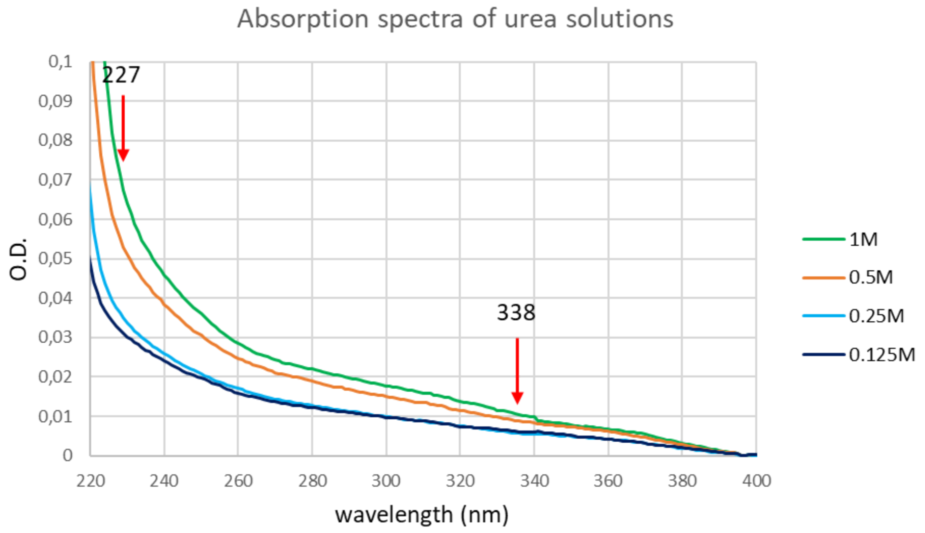

Figure S57. Absorption spectra of urea solutions.

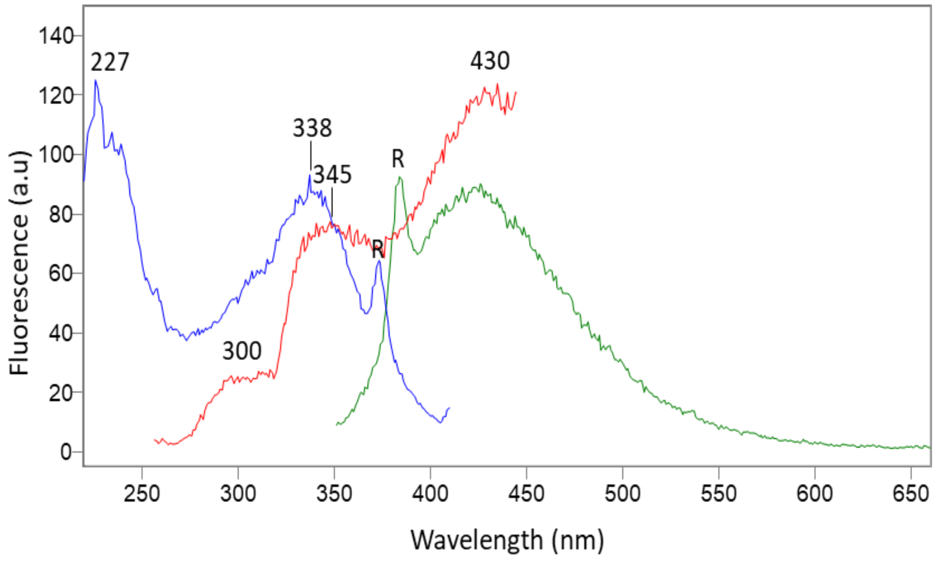

Figure S58. Fluorescence spectra of 1M urea solution. Excitation spectrum with emission at 430 nm (light blue); emission spectrum with excitation at 227 nm (red); emission spectrum with excitation at 338 nm (green). R: Raman spectrum of water. Conditions: spectrofluorometer, Varian Cary Eclipse; photomultiplier gain, 900 V; excitation slit, 5 nm; emission slit, 5 nm.

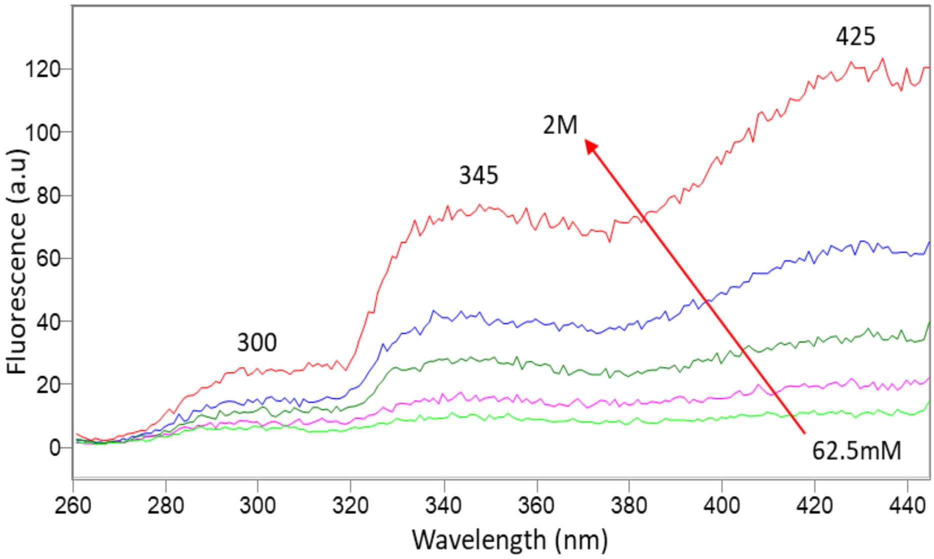

Figure S59. Fluorescence spectra of urea solutions (2M, 1M, 0.5M, 0.25M, 0.125M, 0.0625M); exc=227 nm. Conditions: spectrofluorometer, Varian Cary Eclipse; photomultiplier gain, 900 V; excitation slit, 5 nm; emission slit, 5 nm.

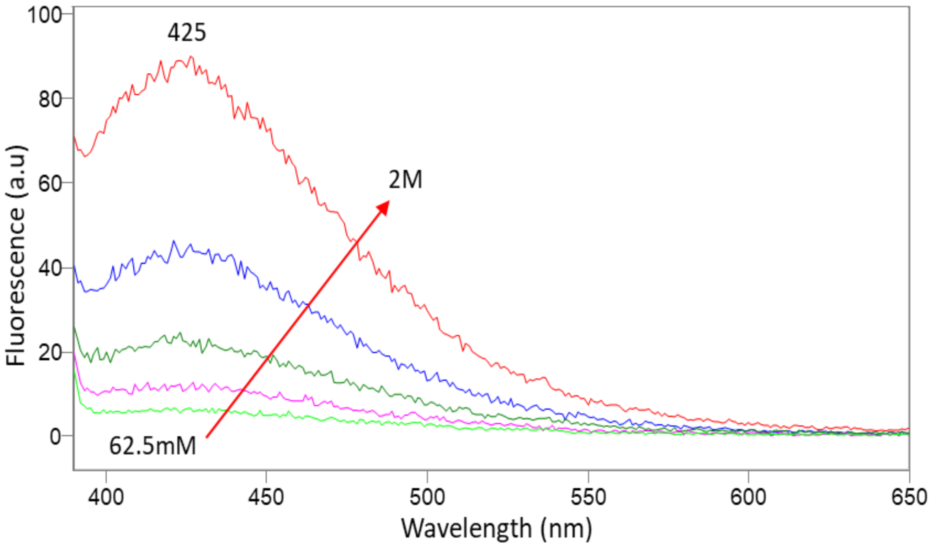

Figure S60. Fluorescence emission spectra of urea solutions (2M, 1M, 0.5M, 0.25M, 0.125M, 0.0625M); exc=338 nm. Conditions: spectrofluorometer, Varian Cary Eclipse; photomultiplier gain, 900 V; excitation slit, 5 nm; emission slit, 5 nm.

Ethanol absorption and fluorescence spectra

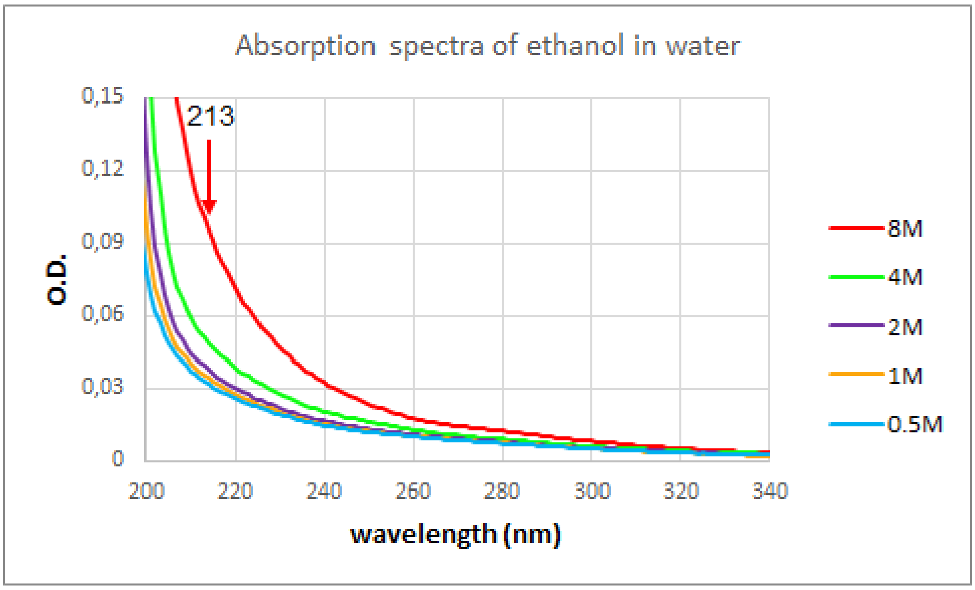

Figure S61. Absorption spectra of ethanol in water

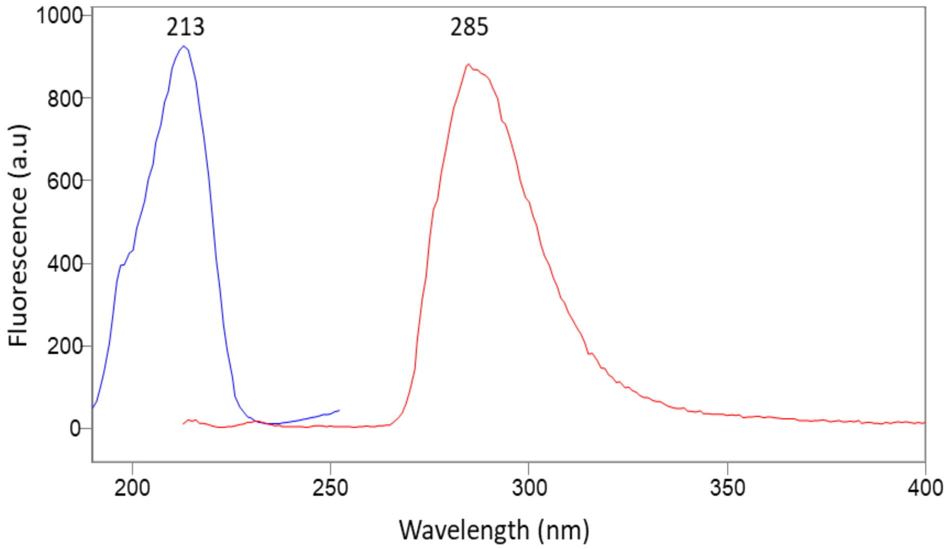

Figure S62. Fluorescence spectra of ethanol 60% (v/v) in water. Excitation spectrum with emission at 285 nm (blue); emission spectrum with excitation at 213 nm (red). Conditions: spectrofluorometer, Varian Cary Eclipse; photomultiplier gain, 800 V; excitation slit, 5 nm; emission slit, 5 nm.

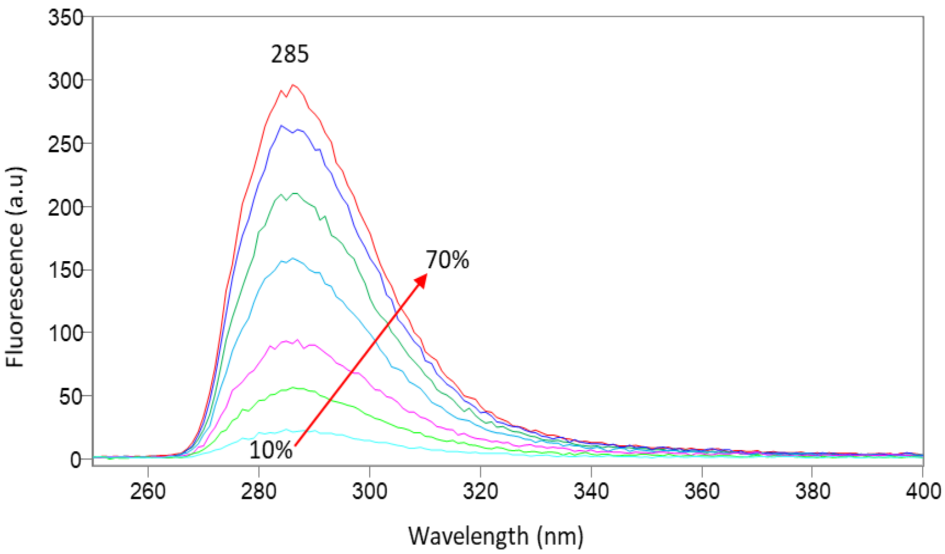

Figure S63. Fluorescence emission spectra of ethanol solutions (from 10% to 70%, v/v in water); exc=213 nm. Conditions: spectrofluorometer, Varian Cary Eclipse; photomultiplier gain, 700 V; excitation slit, 5 nm; emission slit, 5 nm.

Determination of relative fluorescence quantum yields.

We determined the relative quantum yield (QY) for the different solutions by a comparative method using a reference sample with known QY.[1,2,3,4] Phenylalanine (QY=0.024) was employed as reference fluorophore, since its absorption (λ_{max}= 257 nm) and emission (λ_{em}= 280 nm) bands are in the same spectral region of the solutions to test. The absorption and emission spectra of phenylalanine, and of the aqueous solutions were collected at different concentrations in the same instrumental conditions. For each solution, the concentration range was carefully chosen in order to keep the absorbance at the excitation wavelength in the linear range.

Table S1. Relative Fluorescence Quantum Yields (Q_Y with the standard deviation **σ**) of selected systems with excitation wavelengths (exc) in the range 220-227 nm and with emission (em) maxima in the range 240-260 nm and higher than 260 nm.

Solutions	Range 220-227 nm				Range 240-260 nm				Range over 260 nm			
	exc (nm)	em (nm)	Q_Y	σ	exc (nm)	em (nm)	Q_Y	σ	exc (nm)	em (nm)	Q_Y	σ
HCl	220	300	0.018	0.0020								
KCl	227	310	0.002	0.0006	240	430	0.008	0.0010				
NaCl	227	300	0.004	0.0030								
$MgSO_4$	227	340	0.005	0.0021								
Glucose	220	295	0.005	0.0001					330	420	0.014	0.0025
Lactose	227	330	0.007	0.0010								
Trehalose	220	302	0.003	0.0001								
Sorbitol	220	320	0.003	0.0002								
Glycerol	227	305	0.004	0.0020					295	402	0.005	0.0030
Urea	227	270/ 440	0.006	0.0012								
NaOH					240	400	0.005	0.0030				
KOH					240	297	0.002	0.0000				
$(NH_4)_2SO_4$					258	377	0.024	0.0008				
L-glycine					245	438	0.001	0.0000				
L-lysine					258	435	0.010	0.0004				
$CaCl_2$									320	405	0.009	0.0002
$MgCl_2$									310	425	0.011	0.0060
$\langle Q_Y \rangle$			0.006				0.008				0.010	

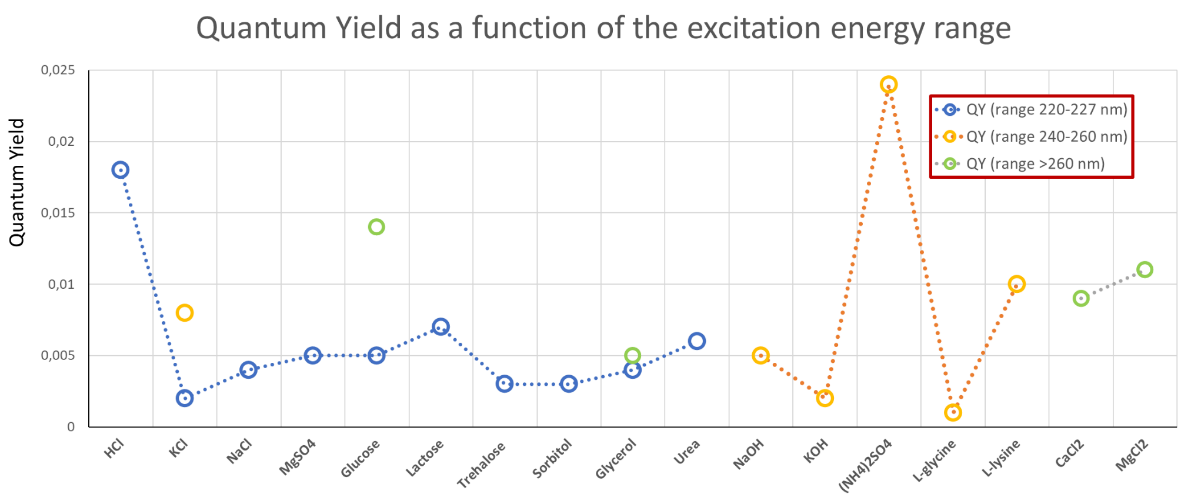

Fig. S64. Relative quantum yields of the examined solutions plotted as a function of the excitation energy range.

Fluorescence emission intensity of 1M NaCl and glycerol solution vs temperature.

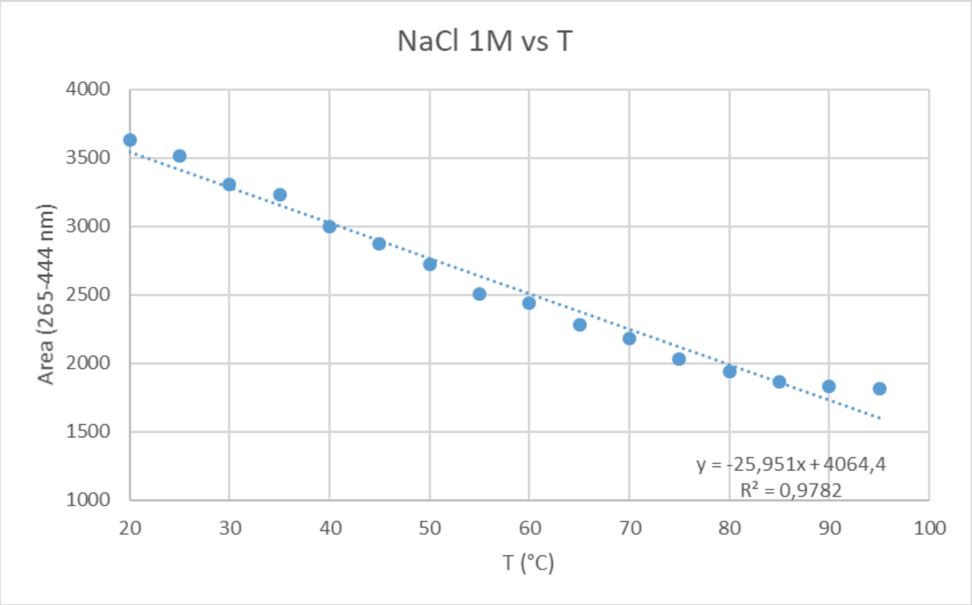

Fig. S65. Fluorescence emission intensity of 1M NaCl water solution at different temperatures from 20°C to 95°C (300 nm band area with excitation at 227 nm).

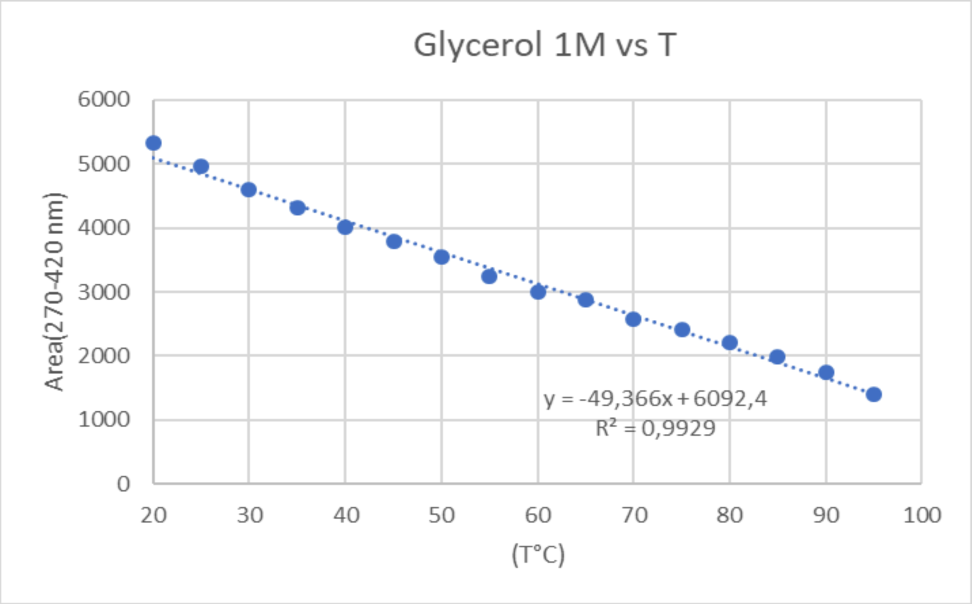

Fig. S66. Fluorescence emission intensity of 1M glycerol water solution at different temperatures from 20°C to 95°C (305 nm band area with excitation at 220 nm).

NaCl FTIR spectra.

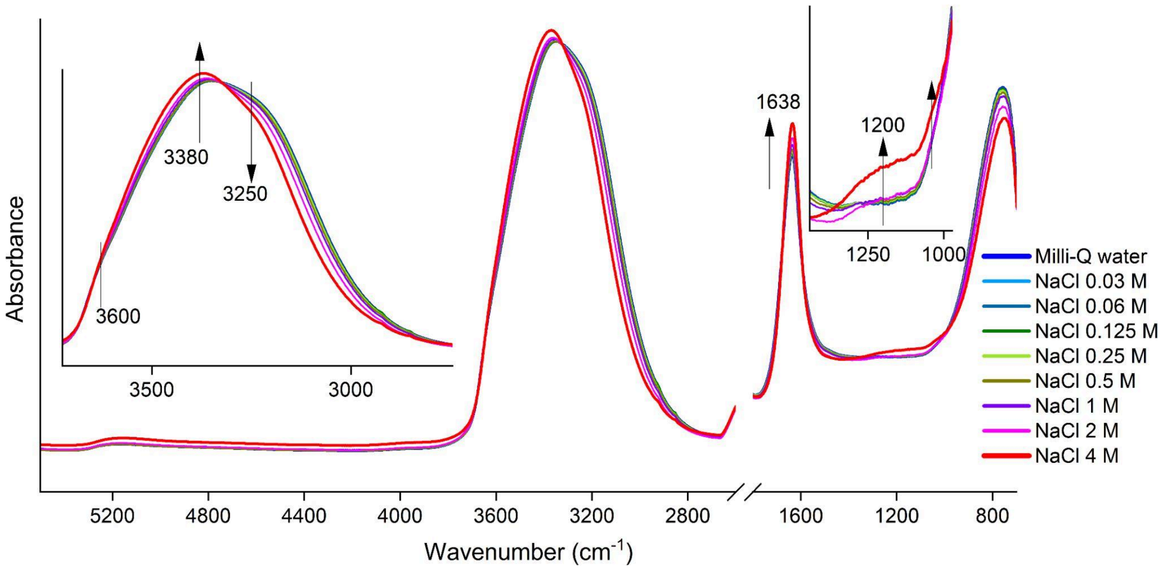

Fig.S67. ATR-FTIR spectra of NaCl aqueous solutions at different concentrations. Raw IR absorption spectra of NaCl aqueous solutions from 4M to 0.03 M are displayed. In each panel, spectra have been reported as measured (i.e. without normalization). For comparison, also the spectrum of water has been reported. Arrows point to NaCl increasing concentrations.

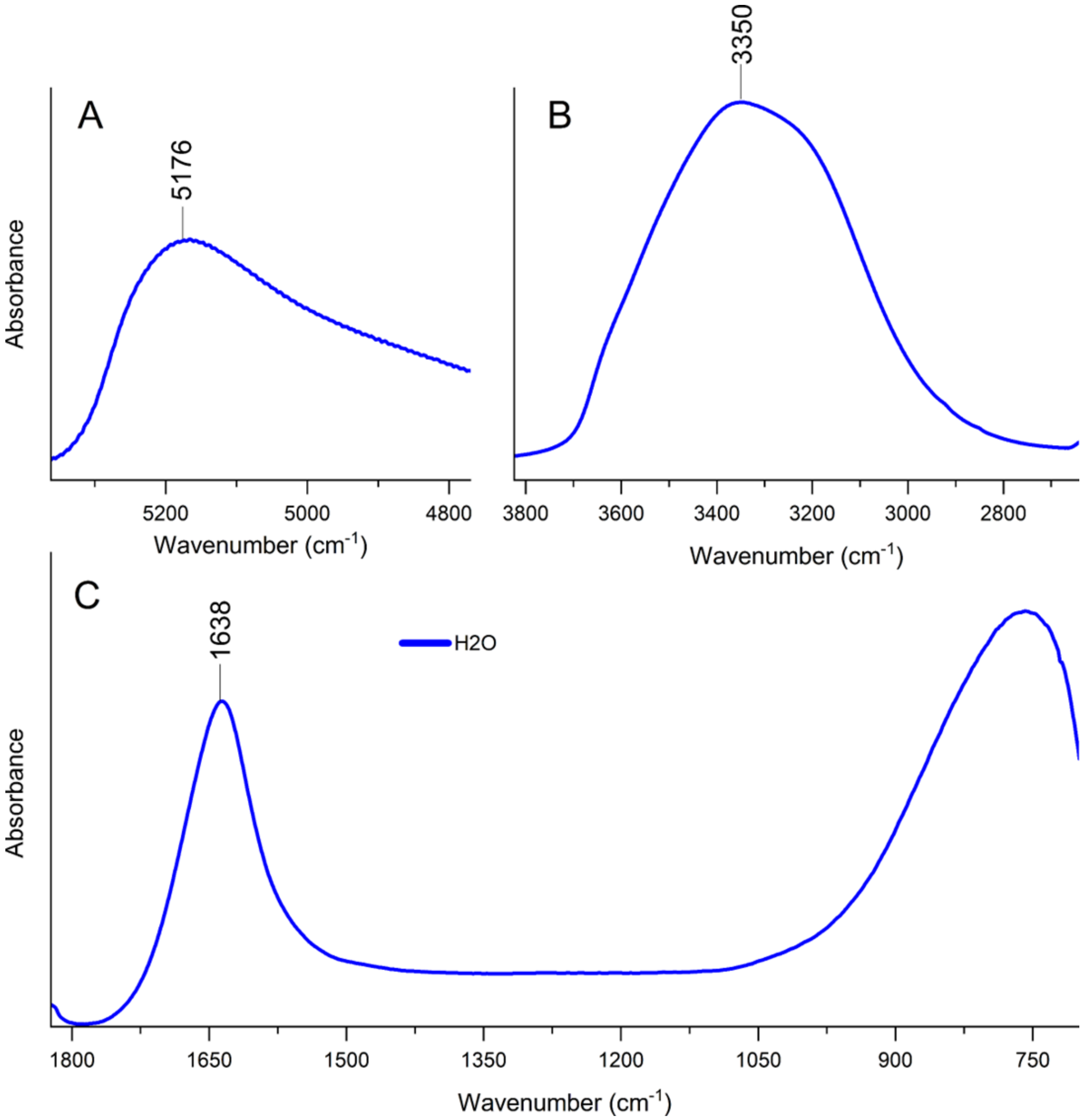
A
5176
Absorbance
5200
5000
4800
Wavenumber (cm-1)
B
3350
3800
3600
3400
3200
3000
2800
Wavenumber (cm-1)
C
1638
H2O
Absorbance
1800
1650
1500
1350
1200
1050
900
750
Wavenumber (cm-1)

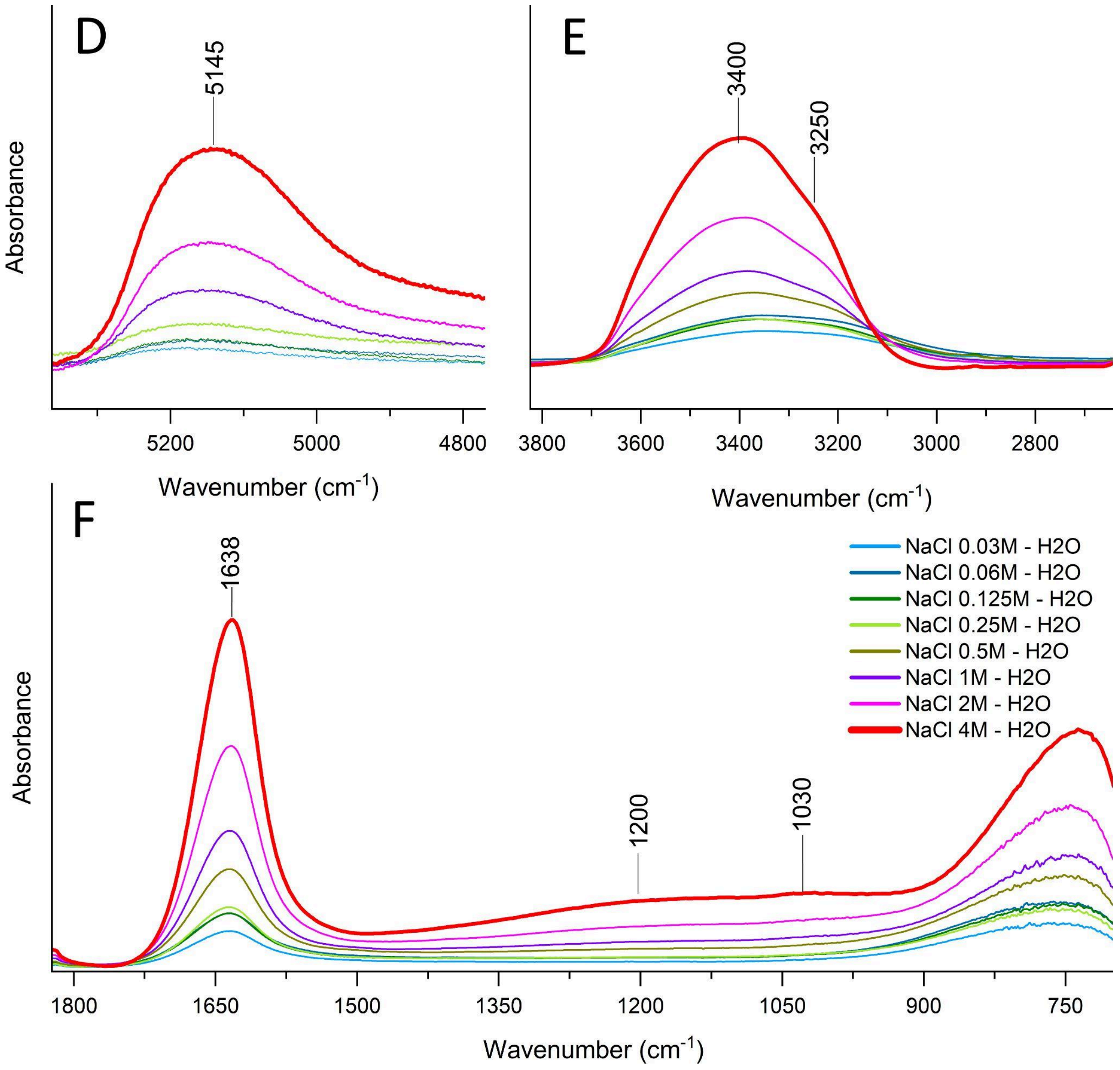

Fig.S68. ATR-FTIR absorption spectra of milliQ water. (A) stretching+combination band; (B) OH stretching band; (C) bending and libration bands. ATR-FTIR absorption spectra of NaCl aqueous solutions from 4M to 0.03 M after subtraction of water spectrum. (D) stretching+bending combination band; (E) OH stretching band; (F) bending and libration bands.

The mid-IR spectrum of liquid water is characterized by three major absorptions (Figure S68 A-C). In particular,

- the most intense absorption band centered at about 3350 cm^{-1} is due to the OH stretching vibration of water;
- the ~ 1638 cm^{-1} band is the bending vibration (v_2);
- the broad band below 800 cm^{-1} is due to water molecule librations (v_L);
- the high frequency band at ~5176 cm^{-1} can be assigned to the combination of the asymmetric stretching and the bending of the water molecule.[5]

By increasing the NaCl concentration, the IR absorption of the solution displays significant differences compared to the pure water spectrum. In particular, we observe a narrowing of the OH stretching band and of the HOH bending, both spectral changes suggesting a structural ordering of water. Moreover, the peak of the stretching band shows an upshift to ~3400 cm^{-1}.

The lowest-frequency band (at about 3250 cm^{-1}) can be assigned to water molecules with an H-bond coordination number close to four, as it occurs at a wavenumber near to that of the OH band observed in ice[6] where strong tetrahedral hydrogen bonds weaken the OH oscillator strength causing a shift to lower frequencies. In this perspective, a possible explanation for the observed intensity decrease in the ~ 3250 cm^{-1} band by increasing NaCl concentration (Figures S67 and S68E) could be related to a reduction in the hydrogen bonding of bulk water molecules, which instead become involved in the solvation shells. The ~1200 cm^{-1} band can be tentatively assigned to proton shuttling of vibrationally coupled water molecules flanking a shared proton[7, 8]

Fourier transform infrared (FTIR) spectra of NaCl aqueous solutions at different concentrations (from 4M to 0.03M) have been collected in the mid-IR range in attenuated total reflection (ATR) by a Varian 670-IR spectrometer (Varian Australia Pty Ltd.) equipped with a nitrogen-cooled Mercury Cadmium Telluride detector. In particular, 20 μL of the sample were deposited on the nine reflections ATR diamond device (DuraSamplIR II, Smith Detection, USA) and spectra were collected by the co-addition of 256 scans, 25 kHz of scan speed, 2 cm^{-1} spectral resolution and triangular apodization. The water spectrum was also measured under the same instrumental conditions.

The IR spectra of NaCl aqueous solutions at different concentrations were corrected for water absorption using the Resolutions-Pro software (Varian Australia Pty Ltd.). In particular, for each NaCl concentration, a water spectrum subtraction factor was selected to prevent the appearance of negative lobes, ensuring that only the bulk water contribution was subtracted.

Arrhenius plots

Arrhenius plot of 1M NaCl and glycerol aqueous solutions.

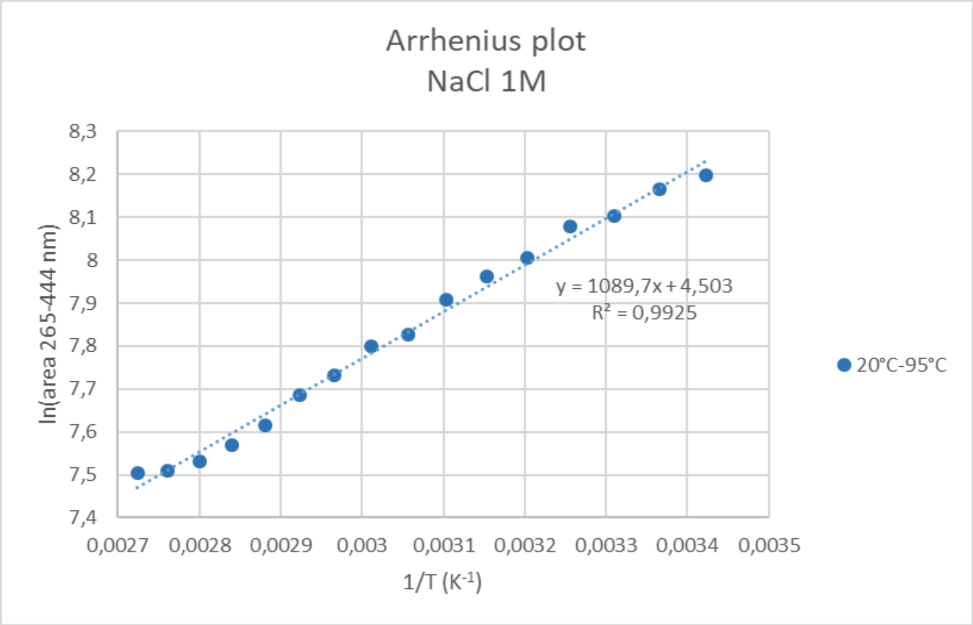

Fig. S69. 1M NaCl Arrhenius plot of the natural logarithm of the emission band area versus 1/T. (300 nm band area with excitation at 227 nm).

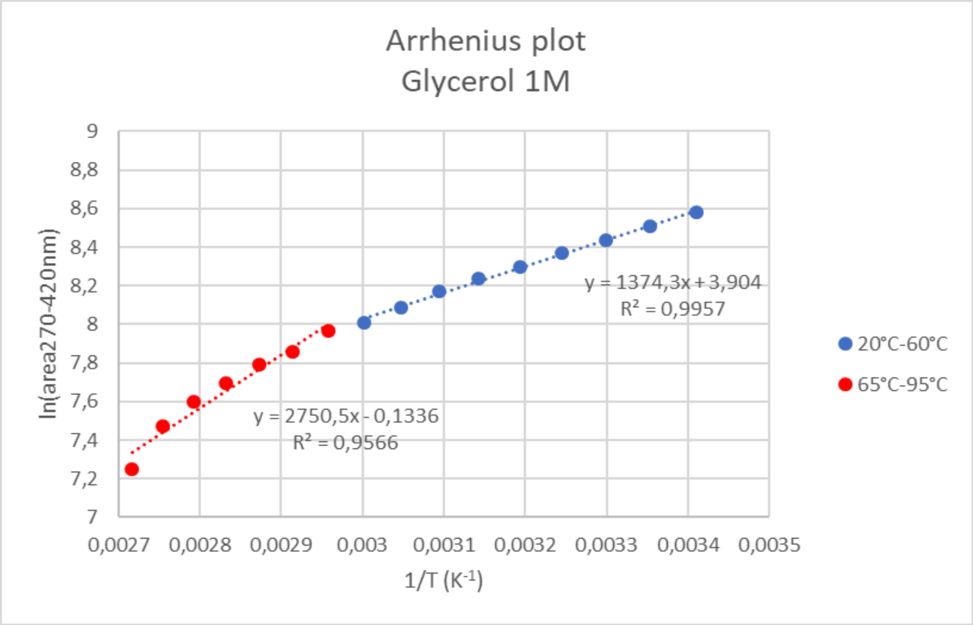

Fig. S70. 1M Glycerol Arrhenius plot of the natural logarithm of the emission band area versus 1/T. (305 nm band area with excitation at 220 nm).

Activation Energy for Radiationless Decay of selected solutes.

The fluorescence quantum yield $\phi_f(T)$ is given by:

$$\phi_f(T) = \frac{k_f}{k_f + k_d} \quad \text{(a)}$$

where k_f is the rate constant for radiative relaxation (fluorescence) and k_d is the sum of the rate constants corresponding to the processes that quench fluorescence and lead to radiationless deactivation. k_d is a function of temperature with the form:

$$k_d(T) = k_d^0 exp\left(-\frac{\Delta E_a}{RT}\right) \quad \text{(b)}$$

where ΔE_a is the activation energy for the temperature dependent radiationless deactivation of the excited fluorescent states.

Following J.M. Menter [9], we can calculate the activation energy ΔE_a using the following equation:

$$ln\left(\frac{1}{\phi_f(T)} - 1\right) = -\frac{\Delta E_a}{RT} + ln\left(\frac{k_d^0}{k_f}\right) \quad \text{(c)}$$

In the limit of low values of $\phi_f(T)$, we can use an approximation of equation (c) and neglect the term -1 in the argument of the logarithm. We obtain the simplified equation [9]:

$$ln\left(\frac{1}{I_N(T)}\right) = -\frac{\Delta E_a}{RT} + lnB \quad \text{(d)}$$

where *I(T)* is the measured fluorescence intensity at temperature T. We assume that the unknown proportionality constant between $\phi_f(T)$ and $I_f(T)$ does not depend on T, so B is a constant. Indeed this assumption is safe since we observe linear Arrhenius kinetics. From eq. (d), the slope of the straight line obtained by plotting $ln\left(\frac{1}{I_N(T)}\right)$ versus 1/T (where T is expressed in kelvin) gives the value of the activation energy.

Table S2. Activation energies (E_a) associated with the nonradiative processes that compete with fluorescence obtained from temperature dependence of fluorescence intensity. The values for HCl and KCl are reported here for sake of completeness and both have been previously measured and published in ref [8,10].

Solution	Concentration	nm	T (°C)	E_a(kJ/mol)	cm^{-1}
Glycerol	1 M	303	≤60	11.5 ± 0.4	959 ± 25
			≥65	20.4 ± 2.4	1741 ± 137
NaCl	1 M	300	20-95	9.6 ± 0.7	802 ± 63
HCl*	3 M	300	20-95	12.5 ± 1.5	1046 ± 177
KCl*	4 M	310	20-95	12.5 ± 1.9	1041 ± 222
		430		7.5 ± 0.2	630 ± 24
L-lysine	2 M	430	20-95	8.7 ± 0.4	727 ± 31

Computations

Classical mechanism protocol. Merck molecular force field 94x (MMFF94x) has been selected for molecular dynamic (MD) simulation since it is well suited to describe the counterions as well as organic molecules. For each system considered ($(H_2O)_{110}$,$(NaCl)_2(H_2O)_{108}$,$(C_3H_8O_3)_2(H_2O)_{108}$) a 100 ns MD run at 298K in implicit water solvent has been carried out with 2.0 fs time step, saving a structure every 20 ps. We obtained a pool of structures for each system. In order to maintain the structure of the spheroidal cluster as much as possible, a centroid spherical wall restraint is set within MD approach. The trajectories for NaCl and glycerol 1M are reported below (Energy corresponds to the structure minimized at the force field level (in kcal/mol), while distances are given in angstroms (Å); for water cluster data not shown)

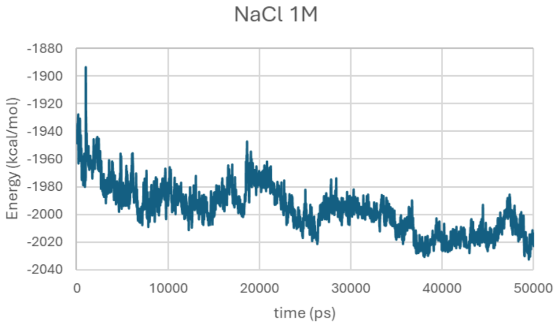

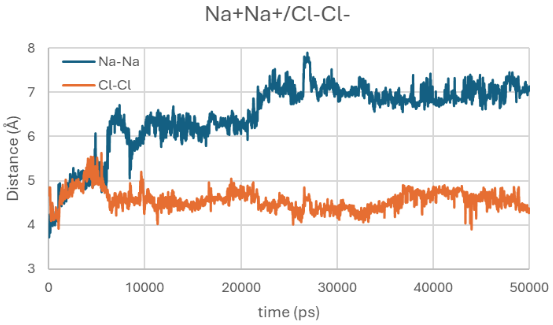

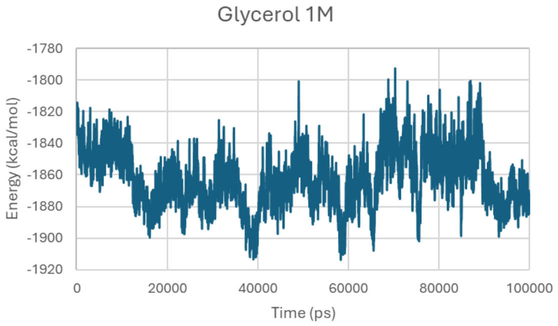

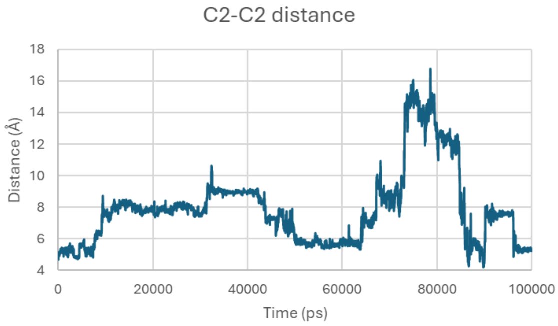

Subsequently, the structures were minimized at the FF (Force Field) level and then arranged in ascending order of total energy. From this pool of optimized geometries, a set of representative structures was chosen for the subsequent DFT geometry optimization. This selection of solute/water structures was aimed at uniformly sampling geometries in which the distance between solutes falls

within the range corresponding to the first three peaks of the radial distribution functions (1 M NaCl, for Na–Na and Cl–Cl distances, the first peak is centered around 4 Å, while the second and third peaks are located around 5.5 Å and approximately 7 Å, respectively; in glycerol, the C2–C2 distance between the central carbon atomsIn glycerol, the C2–C2 distance between the central carbon atoms of the two solute molecules shows a broad peak around ≈ 6 Å thus we consider a C2-C2 distance extending from approximately 4 Å to 8 Å).[11,12]

DFT/TD-DFT protocol. In the two previous articles, the modeling was conducted on 1M solution models using water/solute clusters with the structure $(H_2O)_{110}(solute)_2$. using TURBOMOLE suite of programs,[13] adoption using pure Gradient Generalized Approximation B-LYP functional.[14,15] and triple-ζ plus polarization split valence quality (def-TZVP) basis set. This level of theory nicely reproduces ion properties.[16,17]

At the TD-DFT level, we observed the tendency of certain OH bonds in solute molecules on the outer surface of the cluster to point outward and dissociate during vibrational relaxation on the S1 potential energy surface (PES). To address this phenomenon, we constrained the hydrogen atoms belonging to water molecules on the cluster surface, thereby preventing unphysical O-H bond dissociations. This approach resolved the issue but inevitably focused the study of the S_1 state dynamics on the internal portion of the cluster. In this paper, we observed that by employing a method similar to the 2x1 supercell approach used in solid-state electronic structure calculations, and by doubling the cluster size (from $2\times[(H_2O)_{54}(solute)_1]$ to $(H_2O)_{110}(solute)_2$), it was possible to construct 1M solution models without imposing constraints on any cluster atoms. Using these models, we no longer observed outward-pointing O-H bond dissociations on the cluster surface during vibrational relaxation on the S1 potential energy surface, as simulated through TD-DFT geometry optimization.

Table S3. $(H_2O)_{110}$ clusters. ΔE is the B-LYP/TZVP energy difference in kcal/mol compared with the lowest energy form identified; the H-bonds identified have average O-H distance equal to <O-H> with s<O-H> standard deviation both in Å; 1ex is the band assignment according to the population of the HOMO->LUMO monoelectronic transition and E(S_1) is the excitation energy in nm at the ground state geometry; each optimized structure was subsequently relaxed on the S_1 state using TD-DFT optimization; Cycles is the number of TD-DFT geometry optimization cycles needed to converge to a local S_1 minimum energy or to a conical intersection (CI) with S_0; following the relaxation vibration on S1, two types of final structures are observed: one in which either there is a rearrangement of the H-bond network around the water molecule carrying the positive charge (H_2O H_2O) or even the formation of the radical pair OH and hydronium ion (H_3O^+ OH)

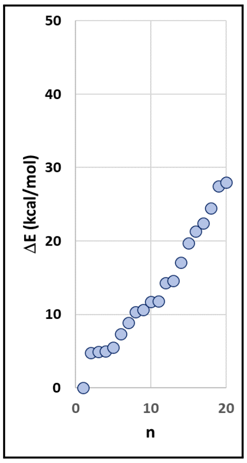

	E(B-LYP) (Hartree)	ΔE kcal/mol	Number Of H-bond	<O-H> (Å)	σ<O-H> (Å)	1ex	E(S_1) nm	Converged or CI?	Cycles	
1	-8410.952546	0.0	194	1.819	0.107	2O→H	253.9	CI	34	H_3O^++OH
2	-8410.944911	4.8	190	1.809	1.111	2O→2H	292.1	CI	24	H_2O H_2O
3	-8410.944803	4.9	191	1.811	0.105	2O→H	272.7	CI	15	H_3O^++OH
4	-8410.944643	5.0	193	1.819	0.129	2O→3H	289.2	CI	9	H_2O H_2O
5	-8410.943713	5.5	191	1.813	0.109	2O→6H	268.4	CI	9	H_2O H_2O
6	-8410.940866	7.3	191	1.813	0.104	O→4H	275.2	CI	13	H_2O H_2O
7	-8410.938396	8.9	190	1.811	0.110	O→4H	253.7	CI	81	H_3O^++OH
8	-8410.936103	10.3	188	1.802	0.090	3O→nH	248.7	CI	15	H_3O^++OH
9	-8410.935647	10.6	190	1.812	0.104	2O→2H	267.2	CI	21	H_3O^++OH
10	-8410.933958	11.7	192	1.812	0.120	2O→2H	247	CI	30	H_3O^++OH
11	-8410.933666	11.8	193	1.82	0.102	O→4H	281.6	CI	9	H_3O^++OH
12	-8410.929815	14.3	193	1.821	0.117	2O→2H	258.1	CI	15	H_2O H_2O
13	-8410.9293	14.6	190	1.814	0.111	2O→5H	272.6	CI	8	H_2O H_2O
14	-8410.925344	17.1	188	1.807	0.098	3O→2H	254.1	CI	26	H_2O H_2O
15	-8410.921202	19.7	195	1.831	0.111	3O→2H	262.2	converged	96	H_2O H_2O
16	-8410.91856	21.3	190	1.816	0.118	3O→3H	270.4	CI	41	H_2O H_2O
17	-8410.916912	22.4	192	1.826	0.130	2O→2H	261	CI	46	H_3O^++OH
18	-8410.913541	24.5	192	1.822	0.110	4O→2H	274.4	CI	88	H_2O H_2O
19	-8410.90866	27.5	191	1.823	0.120	2O→3H	286.9	CI	14	H_3O^++OH
20	-8410.907874	28.0	194	1.831	0.120	2O→6H	287.4	CI	81	H_3O^++OH

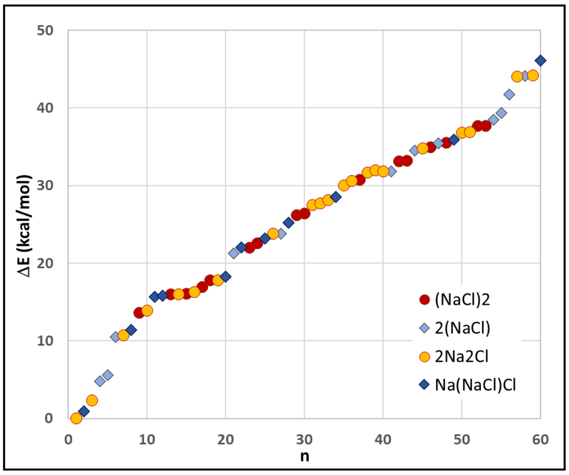

Table S4. $(NaCl)_2(H_2O)_{108}$ clusters. E(B-LYP) are the total energy in hartree; ΔE is the b-lyp/TZVP energy difference in kcal/mol (see picture alongside this table which is also reported in the main text Figure 3); in this column is reported the type of cluster obtained according to the ion positions among each other (2Na2Cl ion not directly interacting; 2(NaCl) ions interact as two salt couple; (NaCl)2 ions forms square or tetrahedral cores (see scheme above); $d(Na^+ \cdot Cl^-)$ is the average value of the four Na···Cl distances in Å; $d(Na^+ \cdot Na^+)$ and $d(Cl^- \cdot Cl^-)$ are Na···Na and Cl···Cl distances in Å; n H_b is the total number of H-bond interaction and <O-H> the corresponding average O-H distance (in Å); 1ex is the band assignment according to the population of the HOMO->LUMO monoelectronic transition and $E(S_1)$ is the excitation energy in nm at the ground state geometry: cycles are the number of TD-DFT optimization cycles on S_1 PES starting form S_0 optimized geometry; this S_1 PES relaxation can converge to a local minimum (converged) or stop when an internal conversion/conical intersection (CI) structure is reached; move column describes the most relevant geometry distortion observed during TD-DFT S_1 relaxation (H_3O^+ OH is for the separation between charge and radical vacancy; H_2O H_2O is for the rearrangement of H-bonds)

n	E(B-LYP) (Hartree)	ΔE	type	ions	d(Na·Cl) (Å)	d(Na·Na) (Å)	d(Cl·Cl)	n H_b	<O-H>	σ<O-H>	1ex	$E(S_1)$	cycles	Converged or CI?	move
1	-9503.2972	0.0	2Na2Cl	buried	6.63	6.21	8.08	174	1.801	0.098	2O→2H	316.4	91	converged	$(H_3O)^+$ OH
2	-9503.2957	0.9	Na(NaCl)Cl	Buried	4.01	5.42	11.64	181	1.816	0.107	Cl→2Na,2H	271.7	8	CI	H detach
3	-9503.2935	2.3	2Na2Cl	Surface	7.14	8.81	13.84	180	1.815	0.111	2O→Na,3H	264.8	13	CI	H_2O+H_2O; Na
4	-9503.2896	4.8	2(NaCl)	Buried	4.65	6.26	7.14	184	1.837	0.103	3O→Na	279.8	57	CI	H_2O+H_2O
5	-9503.2882	5.6	2(NaCl)	Buried	4.61	6.25	7.01	183	1.834	0.102	3O→Na	275.4	64	CI	H_2O+H_2O
6	-9503.2804	10.5	2(NaCl)	Buried	4.71	6.44	7.14	184	1.841	0.106	3O→Na	272.3	323	CI	H_2O+H_2O
7	-9503.2802	10.7	2Na2Cl	Buried	6.39	10.88	7.45	175	1.809	0.104	2O→Na, 4H	328.8	14	CI	H_2O+H_2O
8	-9503.2791	11.4	Na(NaCl)Cl	Buried	4.00	5.82	11.66	180	1.818	0.114	3O→Na,2H	291.1	49	CI	H_2O+H_2O
9	-9503.2755	13.6	(NaCl)2	Buried	3.74	4.79	5.25	189	1.846	0.116	Cl,O→2Na	259.5	18	CI	H_2O+H_2O
10	-9503.275	13.9	2Na2Cl	Buried	5.27	4.36	7.60	177	1.82	0.104	2O→2Na,3H	276.7	111	CI	H_2O+H_2O
11	-9503.2722	15.7	Na(NaCl)Cl	Buried	5.25	5.50	5.67	178	1.822	0.113	Cl,O→2Na	273.2	14	CI	H_2O+H_2O
12	-9503.2721	15.8	Na(NaCl)Cl	Buried	5.25	5.51	5.67	178	1.822	0.113	2O→Na	273.2	54	CI	H_2O+H_2O
13	-9503.2717	16.0	(NaCl)2	Buried	3.41	4.44	4.86	189	1.848	0.135	Cl→2Na	263.8	20	CI	Cl- shift
14	-9503.2717	16.0	2Na2Cl	Surface	7.37	9.11	13.56	177	1.813	0.102	2O→2H	295.2	33	CI	Cl- shift
15	-9503.2715	16.1	(NaCl)2	Buried	3.70	4.76	5.20	189	1.848	0.124	Cl,O→2Na,3 H	264.0	8	CI	H detach
16	-9503.2713	16.3	2Na2Cl	Surface	7.02	8.1	14.22	175	1.814	0.102	O→Na,5H	275.1	17	CI	$(H_3O)^+$ OH
17	-9503.2702	16.9	(NaCl)2	Buried	7.38	9.11	13.56	177	1.813	0.105	Cl→2Na,2H	276.6	12	CI	Na shift
18	-9503.2689	17.8	(NaCl)2	Buried	4.24	5.93	5.68	178	1.821	0.117	3O→Na,H	283.1	139	converged	H_2O+H_2O
19	-9503.2688	17.8	2Na2Cl	surface	7.25	9.93	18.12	179	1.816	0.101	Cl→Na,4H	294.0	11	CI	Cl detach
20	-9503.2681	18.3	Na(NaCl)Cl	Buried	3.79	4.15	5.07	179	1.82	0.112	Cl→2O,4H	284.4	9	CI	H detach
21	-9503.2632	21.3	2(NaCl)	Buried	5.29	6.83	6.79	177	1.825	0.105	3O→2Na,2H	275.1	554	converged	H_2O+H_2O
22	-9503.2622	22.0	Na(NaCl)Cl	Buried	4.76	4.81	6.85	177	1.816	0.114	Cl,O→H,Na	271.9	10	CI	H_2O+H_2O
23	-9503.2621	22.0	(NaCl)2	Buried	3.45	4.51	4.49	182	1.828	0.122	2O→Na,H	278.8	44	CI	H_2O+H_2O
24	-9503.2612	22.6	(NaCl)2	Buried	4.3	5.82	6.28	177	1.82	0.118	2O→2Na	264.5	23	CI	H_2O+H_2O
25	-9.503.2602	23.2	Na(NaCl)Cl	Buried	7.37	4.87	4.92	183	1.827	0.112	Cl→2Na,2H	276.5	12	CI	H_2O+H_2O
26	-9503.2592	23.8	2Na2Cl	Buried	5.22	6.26	8.13	177	1.818	0.106	2O→Na, H	268.0	74	converged	H_2O+H_2O
27	-9503.2592	23.8	2(NaCl)	Buried	8.05	6.26	8.13	177	1.817	0.106	2O→Na,4H	268.0	73	converged	H_2O+H_2O
28	-9503.2570	25.2	Na(NaCl)Cl	Buried	5.36	4.77	7.68	174	1.816	0.102	2O→Na,3H	269.3	13	CI	H_2O+H_2O
29	-9503.2555	26.2	(NaCl)2	Buried	5.42	8.31	6.94	179	1.826	0.117	2O→Na, H	276.9	71	converged	H_2O+H_2O
30	-9503.2552	26.4	(NaCl)2	Buried	8.59	8.31	6.94	179	1.826	0.116	2O→Na,3H	277.0	71	converged	H_2O+H_2O
31	-9503.2533	27.5	2Na2Cl	surface	8.05	6.63	15.56	175	1.812	0.108	Cl→Na,2H	282.2	20	CI	$(H_3O)^+$ OH

32	-9503.2531	27.7	2Na2Cl	Buried	4.83	7.2	7.27	176	1.825	0.112	2O→2Na	268.5	11	Cl	H_2O+H_2O
33	-9503.2524	28.1	2Na2Cl	Buried	4.77	6.65	6.64	175	1.806	0.095	2O→Na,2H	276.0	8	Cl	$(H_3O)^+$ OH
34	-9.503.2518	28.5	Na(NaCl)Cl	Buried	3.97	4.22	5.71	178	1.82	0.121	O→2Na,3H	269.0	10	Cl	$(H_3O)^+$ OH
35	-9503.2494	30.0	2Na2Cl	Buried	6.18	6.57	10.21	180	1.831	0.134	Cl,O→2Na	280.0	70	Cl	Na^+ shift
36	-9503.2484	30.6	2Na2Cl	surface	8.59	9.88	14.94	176	1.828	0.11	Cl→Na,2H	295.2	17	Cl	Na^+ shift
37	-9503.2483	30.7	(NaCl)2	Buried	3.38	4.75	4.45	195	1.878	0.125	2O→2Na	254.8	42	Cl	H_2O+H_2O
38	-9503.2467	31.7	2Na2Cl	Buried	6.47	5.91	10.65	184	1.861	0.12	4O→nH	282.0	24	Cl	$(H_3O)^+$ OH
39	-9503.2465	32	2Na2Cl	Buried	5.13	4.50	5.01	173	1.815	0.113	2O→2Na,3H	257.8	118	converged	H_2O+H_2O
40	-9503.2465	31.8	2Na2Cl	Buried	5.74	7.27	8.47	178	1.83	0.121	Cl→Na,O	274.7	10	Cl	H detach
41	-9503.2465	31.8	2(NaCl)	Buried	5.13	5.9	8.34	173	1.815	0.113	2O→Na,3H	257.8	119	converged	H_2O+H_2O
42	-9503.2445	33.1	(NaCl)2	Buried	4.74	5.76	7.08	175	1.817	0.115	2O→Na,3H	271.1	13	Cl	$(H_3O)^+$ OH
43	-9503.2442	33.2	(NaCl)2	Buried	4.14	4.45	5.82	172	1.807	0.095	2O→2Na	261.2	268	Cl	H detach
44	-9503.2422	34.5	2(NaCl)	Buried	3.08	4.14	4.52	182	1.835	0.115	O→Na,3H	294.1	157	Cl	H_2O+H_2O
45	-9503.2418	34.8	2Na2Cl	Buried	5.94	6.21	10.03	177	1.828	0.111	O→Na,2H	295.9			$(H_3O)^+$ OH
46	-9503.2416	34.9	(NaCl)2	Buried	2.91	4.06	4.10	181	1.828	0.132	3O→Na,2H	278.6	24	Cl	H_2O+H_2O
47	-9503.2408	35.4	2(NaCl)	Buried	5.99	7.64	9.54	176	1.823	0.113	2O→2Na,2H	312.8	98	converged	H_2O+H_2O
48	-9503.2406	35.5	(NaCl)2	Buried	3.95	5.96	4.37	180	1.829	0.11	Cl,O→2Na,3H	259.7	234	converged	H_2O+H_2O
49	-9.503.2401	35.9	Na(NaCl)Cl	Buried	3.81	4.75	6.21	177	1.817	0.112	3O→2Na	268.46	424	converged	H_2O+H_2O
50	-9503.2386	36.8	2Na2Cl	Buried	8.12	4.81	5.42	180	1.82	0.126	2O→2Na,2H	268.3	83	converged	H detach
51	-9503.2385	36.9	2Na2Cl	Buried	3.64	5.18	4.9	177	1.82	0.113	2O→2Na,2H	277.0	11	Cl	$(H_3O)^+$ OH
52	-9503.2371	37.7	(NaCl)2	Buried	3.72	4.62	5.92	179	1.84	0.111	2O→Na,2H	263.6	10	Cl	H_2O+H_2O
53	-9503.2267	37.7	(NaCl)2	Buried	3.22	4.24	4.60	176	1.81	0.116	O2→Na,2H	274.6	269	converged	H detach
54	-9503.2359	38.5	2(NaCl)	Buried	6.09	7.61	9.59	174	1.82	0.103	Cl→2Na,2H	267.1	23	Cl	H detach
55	-9503.2345	39.3	2(NaCl)	Buried	6.28	9.95	13.01	176	1.82	0.108	2O→Na,4H	290.1	54	Cl	H_2O+H_2O
56	-9.503.2308	41.7	2(NaCl)	Buried	4.98	2.78	2.83	179	1.82	0.111	2O→2Na	279.4	16	Cl	H_2O+H_2O
57	-9503.2271	44.0	2Na2Cl	Buried	6.05	6.04	6.32	174	1.83	0.11	3O→Na,5H	284.1	8	Cl	H_2O+H_2O
58	-9503.2269	44.1	2(NaCl)	Buried	3.55	10.40	4.74	178	1.831	0.111	2O→Na,H	275.3	17	Cl	H_2O+H_2O
59	-9503.2267	44.2	2Na2Cl	Buried	6.34	6.56	10.84	174	1.82	0.119	3O→Na,3H	277.1	246	converged	H_2O+H_2O
60	-9503.2238	46.1	Na(NaCl)Cl	surface	6.79	8.09	13.59	181	1.83	0.111	Cl→2Na,3H	288.4	11	Cl	Cl- shift

Table S5. $(C_3H_6O_3)_2(H_2O)_{108}$ clusters. E(B-LYP) are the total energy in hartree; ΔE is the b-lyp/TZVP energy difference in kcal/mol (see picture alongside this table which is also reported in the main text Figure 3); in the type column is reported the type of cluster obtained according to the ion of the t2o glycerol molecules (SIP and CIP) d(C2-C2) is the distance between the central carbon atoms of the two glycerol molecules in Å; n H_b is the total number of H-bond interaction and <O-H> the corresponding average O-H distance (in Å); 1ex is the band assignment according to the population of the HOMO->LUMO monoelectronic transition and $E(S_1)$ is the excitation energy in nm at the ground state geometry: cycles are the number of TD-DFT optimization cycles on S_1 PES starting form S_0 optimized geometry; this S_1 PES relaxation can converge to a local minimum (converged) or stop when an internal conversion/conical intersection (CI) structure is reached; move column describes the most relevant geometry distortion observed during TD-DFT S_1 relaxation (H_3O^+ OH is for the separation between charge and radical vacancy; H_2O H_2O is for the rearrangement of H-bonds, C-C bond breaking then a C-C bond results stretched or dissociated.

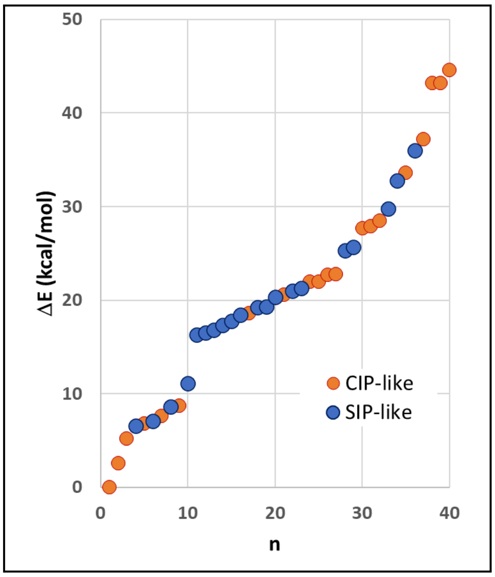

n	type	E(B-LYP) (Hartree)	dE	d(C2-C2) (Å)	H-bond	n Hb	<O-H> (Å)	sig<O-H> (Å)	1ex	$E(S_1)$ (nm)	cycles	conv?	move
1	CIP	-8947,68138	0,0	4,45	2	209	1,85	0,158	3O->3H	272,1	52	CI	$(H_3O)^+$ OH
2	CIP	-8947,67720	2,6	4,53	2	206	1,843	0,163	3O->3H	279	7	CI	$(H_3O)^+$ OH
3	CIP	-8947,67306	5,2	4,47	2	206	1,837	0,127	2O->4H	265	3	converged	H_2O H_2O
4	SIP	-8947,67087	6,6	5,49	1	207	1,845	0,163	gly->H	302,6	7	CI	C-C bond break
5	CIP	-8947,67060	6,8	6,79	1	198	1,816	0,11	2O->4H	263,3	25	converged	H2O H2O
6	SIP	-8947,67012	7,1	10,42	0	200	1,823	0,131	gly->4H	283,1	7	CI	C-C bond break
7	CIP	-8947,66930	7,6	4,48	1	204	1,841	0,154	3O->3H	283,6	50	converged	H_2O H_2O
8	SIP	-8947,66765	8,6	10,47	0	204	1,837	0,148	gly->2H	261,8	116	converged	H detach
9	CIP	-8947,66748	8,7	5,23	1	202	1,83	0,134	gly->2H	280,6	11	CI	H_2O H_2O
10	SIP	-8947,66370	11,1	6,38	0	203	1,835	0,149	2O->3H	258,3	12	CI	$(H_3O)^+$ OH
11	SIP	-8947,65534	16,3	5,67	1	204	1,84	0,146	1O->6H	292,8	294	converged	H_2O H_2O
12	SIP	-8947,65510	16,5	6	1	202	1,833	0,134	3O->4H	293,2	138	converged	$(H_3O)^+$ OH
13	SIP	-8947,65465	16,8	5,48	1	202	1,825	0,117	3O->1H	275,5	577	CI	H_2O H_2O
14	SIP	-8947,65379	17,3	5,73	0	201	1,83	0,144	2O->4H	265,8	22	CI	$(H_3O)^+$ OH
15	SIP	-8947,65309	17,8	6,37	1	205	1,844	0,143	2O->3H	266,2	201	converged	$(H_3O)^+$ OH
16	SIP	-8947,65210	18,4	5,95	1	201	1,83	0,13	3O->4H	267,9	265	converged	H detach
17	CIP	-8947,65170	18,6	4,58	2	203	1,84	0,135	2O->6H	275,3	6	CI	H_2O H_2O
18	SIP	-8947,65078	19,2	6,66	0	206	1,844	0,14	2O->4H	300,2	9	CI	H_2O H_2O
19	SIP	-8947,65060	19,3	6,64	0	206	1,847	0,162	2O->2H	267,1	9	CI	(H3O)+OH
20	SIP	-8947,64900	20,3	6,35	0	202	1,836	0,144	4O->2H	252	44	converged	C-C bond break
21	CIP	-8947,64849	20,6	5,5	1	202	1,835	0,14	2O->4H	272	230	converged	$(H_3O)^+$ OH
22	SIP	-8947,6479	21,0	5,51	1	203	1,84	0,146	2O->4H	270	12	CI	H_2O H_2O
23	SIP	-8947,64750	21,3	7	0	203	1,837	0,132	3O->7H	293	7	CI	
24	CIP	-8947,64633	22,0	6,77	1	201	1,835	0,14	gly->4H	278,1	16	CI	C-C bond break
25	CIP	-8947,64629	22,0	6,77	1	201	1,835	0,14	gly->6H	278,4	16	CI	C-C bond break
26	CIP	-8947,64521	22,7	6,83	1	198	1,823	0,108	gly->2H	263,6	462	converged	
27	CIP	-8947,64510	22,8	4,38	2	200	1,837	0,137	2O->5H	260,8	12	CI	H detach
28	SIP	-8947,64103	25,3	6,12	1	202	1,834	0,125	2O->6H	280,9	14	CI	H_2O H_2O
29	SIP	-8947,64050	25,7	7,52	0	210	1,861	0,162	3O->2H	292,2	8	CI	$(H_3O)^+$ OH
30	CIP	-8947,63717	27,7	4,46	2	206	1,853	0,15	2O->5H	283,2	2	CI	H_2O H_2O

31	CIP	-8947,63689	27,9	4,36	2	201	1,837	0,143	2O->4H	253,1	534	converged	H_2O H_2O
32	CIP	-8947,63588	28,5	4,64	1	203	1,842	0,151	3O->3H	276,1	6	Cl	H_2O H_2O
33	SIP	-8947,63396	29,8	4,6	1	205	1,85	0,155	3O->5H	275,7	10	Cl	O-H dissociation
34	SIP	-8947,62905	32,8	6,08	1	206	1,851	0,161	2O->1H	279,9	23	Cl	H_2O H_2O
35	CIP	-8947,62788	33,6	5,3	1	199	1,833	0,126	3O->nH	238,7	125	converged	H_2O H_2O
36	SIP	-8947,62405	36,0	5,71	1	202	1,84	0,125	2O->3H	261,8	13	Cl	$(H_3O)^+$ OH
37	CIP	-8947,62212	37,2	4,63	1	204	1,85	0,156	gly->5H	263,7	7	Cl	C-C bond break
38	CIP	-8947,61256	43,2	4,74	1	202	1,845	0,132	1O->4H	258,9	11	Cl	$(H_3O)^+$ OH
39	CIP	-8947,61256	43,2	4,74	1	202	1,845	0,132	2O->4H	258,9	14	Cl	$(H_3O)^+$ OH
40	CIP	-8947,61031	44,6	4,42	3	208	1,861	0,155	gly->6H	274,9	9	Cl	C-C bond break

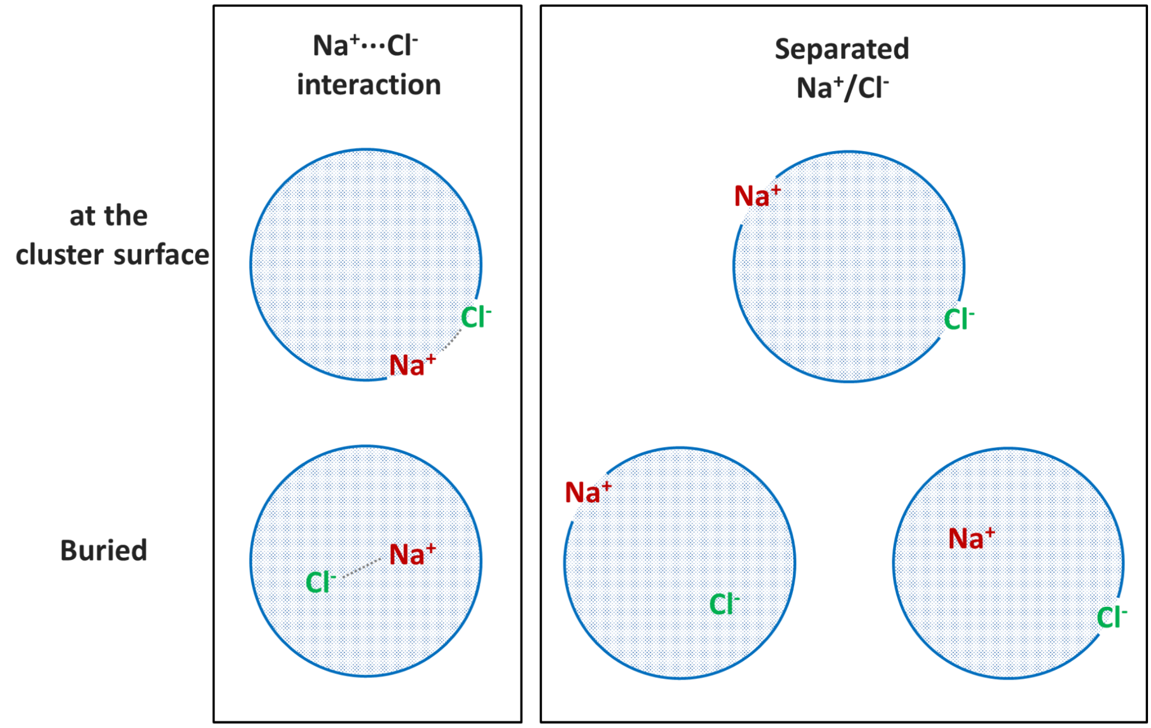

Fig. S71. General scheme for the 60 geometries for $(NaCl)_2(H_2O)_{108}$, clusters. The structure can be distinguished as contact ion pairs structures and solvent-shared ion-pair structures with ions at the surface or ions buried in the cluster.

Table S6. In the following table we report the comparison of structural and electronic structure data computed as a function of the DFT functional for the most stable $(H_2O)_{110}$, $(NaCl)_2(H_2O)_{108}$ and $(C_3H_6O_3)_2(H_2O)_{108}$ cluster (structure 1 in the tables above). In the picture are put in evidence the distance donor acceptor considered in the tables.

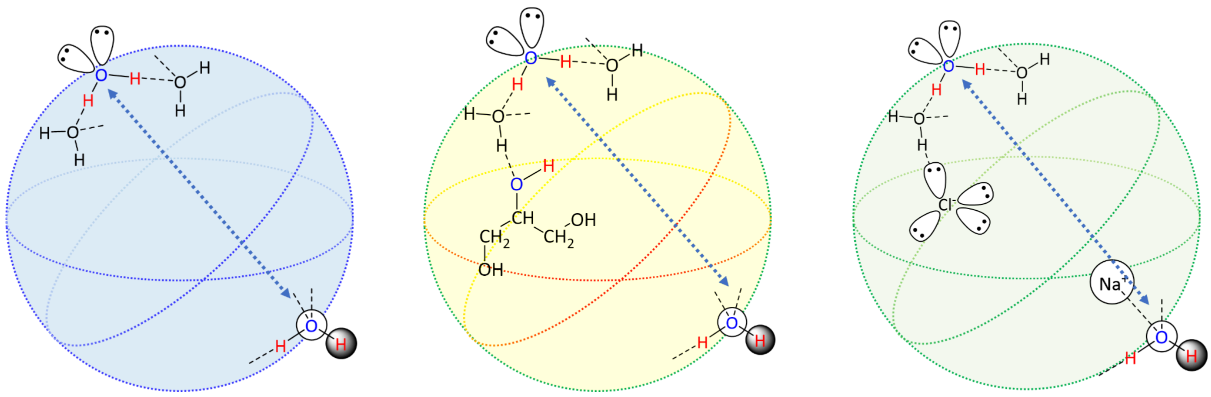

Total energy of the systems (in hartree); **dQ** is the sum of atomic charge differences between the S_1 and S_0 wavefunctions, calculated via NBO analysis and considering only atoms with a charge difference ≥ +0.1 electrons. This value reflects the extent of the most significant charge transfer (CT) involved in the $S_0 \rightarrow S_1$ excitation at the S_0 equilibrium geometry. **n Hb** is the number of O–H hydrogen bonds, with the average O–H distance and its standard deviation reported as **⟨O–H⟩ ± σ⟨O–H⟩** (in Å); **1ex** indicates the atoms involved in the primary mono-electronic transition corresponding to the HOMO → LUMO excitation, with the HOMO–LUMO gap reported in atomic units; **S_1** is the excitation wavelength (in nm).

**$(H_2O)_{110}$**										
**DFT functional**	**Total eneregy** (hartree)	**Dist O->H** CT (Å)	**dQ**			**n Hb**	**<O-H>± σ<O-H>** (Å)	**1ex**	**HOMO/ LUMO Gap** (au)	**S_1** (nm)
b-lyp	-8410.9525465	14,98	0,85			194	1,819±0,107	2O->H	0,179	253,9
b3-lyp[15,18]	-8408.8198846	14,91	0,84			194	1,824±0,104	2O->H	0.259	184,5
pbe[19]	-8403.7560768	14,71	0,82			195	1,757±0,113	2O->5H	0,185	248,1
pbe0[20]	-8403.6194192	14,75	0,81			195	1,786±0,108	2O->6H	0,284	170,3
tpss[21]	-8413.1087966	14,79				195	1,778±0,114	2O->6H	0,198	229,2
tpssh[22]	-8412.1182330	14,96				193	1,793±0,114	2O->2H	0,235	197.5

**$(H_2O)_{110}$ cluster structures whose ΔE is lower than 10 kcal/mol compared to the most stable form**										
**DFT functional B-LYP**	**Total eneregy** (hartree)	**ΔE** kcal/mol				**n H**	**<O-H>± σ<O-H>** (Å)	**1ex**	**HOMO /LUMO Gap** (au)	**S_1** (nm)
1	-8410.952546	0.0	0.85			194	1.819±0.107	2O→H	0.179	253.9
2	-8410.944911	4.8	0.83			190	1.809±1.111	2O→2H	0.156	292.1
3	-8410.944803	4.9	0.85			191	1.811±0.105	2O→H	0.167	272.7
4	-8410.944643	5.0	0.83			193	1.819±0.129	2O→3H	0.159	289.2
5	-8410.943713	5.5	0.85			191	1.813±0.109	2O→6H	0.170	268.4
6	-8410.940866	7.3	0.82			191	1.813±0.104	O→4H	0.167	275.2
7	-8410.938396	8.9	0.85			190	1.811±0.110	O→4H	0.180	253.7

**NaCl**										
**DFT functional**	**Total energy** (hartree)	**Dist O->H** CT (Å)	dQ	**d Na^+-Na^+** (Å)	**d Cl^--Cl^-** (Å)	**n Hb**	**<O-H>± σ<O-H>** (Å)	**1ex**	**HOMO/ LUMO Gap** (au)	**S_1** (nm)
b-lyp	-9503.2972	11,07	0.84	6.21	8.08	174	1,801±0,098	2O->6H	0.172	316,4
b3-lyp[15,18]	-9501.0566	14,8	0,79	6,15	8,08	175	1,805±0,095	3O->6H	0,223	214,1
pbe[19]	-9495.6240	10,79	0,83	6,11	7,92	173	1,730±0,101	2O->6H	0.147	310,0
pbe0[20]	-9495.5993	10,78	0,82	6,13	7,95	174	1,744±0,101	2O->6H	0,148	308,9
tpss[21]	-9505.49307	9,14		6,19	7,99	174	1,759±0,099	2O->6H	0,161	281,9
tpssh[22]	-9505.4693	10,95		6,20	8,00	174	1,761±0,100	2O->6H	0,162	231,4
**Glycerol**										
**DFT functional**	**Total energy** (hartree)	**Dist C2-C2** (Å)	**dQ**			**n Hb**	**<O-H>± σ<O-H>** (Å)	**1ex**	**HOMO/ LUMO Gap** (au)	**S_1** (nm)
b-lyp	-8947,68138	4,455	0.77			209	1,850±0,158	3O->3H	0.167	272,1
b3-lyp[15,18]	-8945,435232	4,444	0,84			209	1,852±0,152	3O->2H	0.249	192,0
pbe[19]	-8940,034032	4,347	0.77			208	1,800±0,172	3O->4H	0.170	267,5
pbe0[20]	-8939,927726	4,362	0.82			210	1,818±0,165	3O->2H	0.274	176,7
tpss[21]	-8950,082921	4,398				209	1,815±0,174	3O->6H	0,185	245,8
tpssh[22]	-8949,03569	4,401				209	1,823±0,164	3O->4H	0,273	206,6

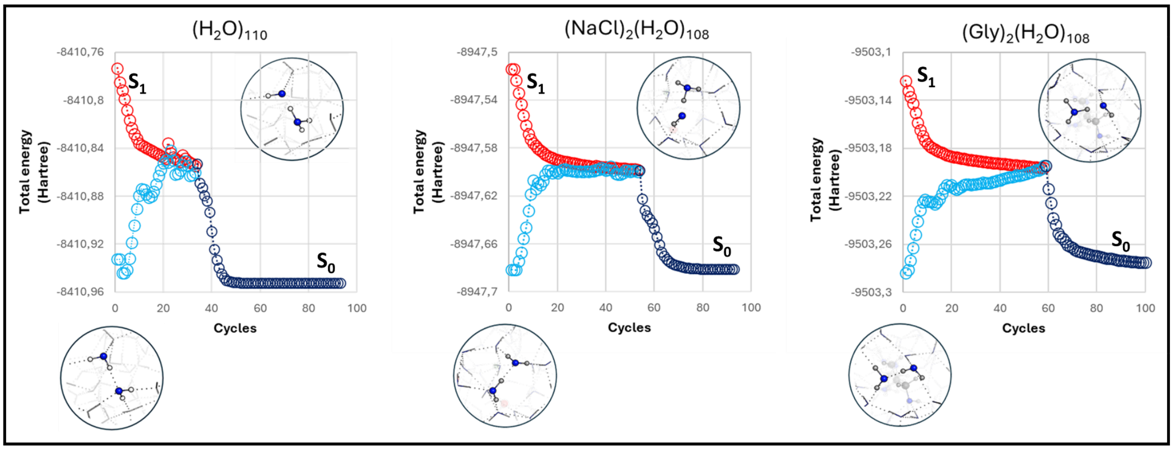

Fig. S72. The total energies of the S_1 and S_0 states along the S_1 recombination coordinate were simulated using TD-DFT geometry optimization, starting from the DFT minimum energy structures of water, water/NaCl, and water/glycerol clusters identified at the DFT level. The energy of S_1 is depicted in red, while the corresponding energy of S_0 is shown in cyan. After the S_1/S_0 crossing, the geometry optimization was continued at the DFT level, reverting to the initial structure. Specifically, the number of TD-DFT geometry optimization cycles required were 33 for the water cluster, 58 for the water/NaCl cluster, and 60 for the water/glycerol cluster.

References

(1) Levitus, M. Tutorial: Measurement of Fluorescence Spectra and Determination of Relative Fluorescence Quantum Yields of Transparent Samples. *Methods Appl Fluoresc* **2020**, *8* (3), 033001.
(2) Rhys Williams, A. T.; Winfield, S. A.; Miller, J. N. Relative Fluorescence Quantum Yields Using a Computer-Controlled Luminescence Spectrometer. *Analyst* **1983**, *108* (1290), 1067–1071.
(3) Lakowicz, J. R. Principles of Fluorescence Spectroscopy. 1999. https://doi.org/10.1007/978-1-4757-3061-6.
(4) Chen, R. F. Fluorescence Quantum Yields of Tryptophan and Tyrosine. *Anal. Lett.* **1967**, *1* (1), 35–42.
(5) Weyer, L. G.; Lo, S.-C. Spectra- Structure Correlations in the near-Infrared. *Handbook of Vibrational Spectroscopy*; Griffiths, P. R., Ed.; John Wiley & Sons, Ltd: Chichester, UK, 2006. https://doi.org/10.1002/0470027320.s4102.
(6) Brubach, J.-B.; Mermet, A.; Filabozzi, A.; Gerschel, A.; Roy, P. Signatures of the Hydrogen Bonding in the Infrared Bands of Water. *J Chem Phys* **2005**, *122* (18), 184509.
(7) Thämer, M.; De Marco, L.; Ramasesha, K.; Mandal, A.; Tokmakoff, A. Ultrafast 2D IR Spectroscopy of the Excess Proton in Liquid Water. *Science* **2015**, *350* (6256), 78–82.
(8) Villa, A. M.; Doglia, S. M.; De Gioia, L.; Bertini, L.; Natalello, A. Anomalous Intrinsic Fluorescence of HCl and NaOH Aqueous Solutions. *J Phys Chem Lett* **2019**, *10* (22), 7230–7236.
(9) Menter, J. M. Temperature Dependence of Collagen Fluorescence. *Photochem. Photobiol. Sci.* **2006**, *5* (4), 403–410.
(10) Villa, A. M.; Doglia, S. M.; De Gioia, L.; Natalello, A.; Bertini, L. Fluorescence of KCl Aqueous Solution: A Possible Spectroscopic Signature of Nucleation. *J Phys Chem B* **2022**, *126* (13), 2564–2572.
(11) Mancinelli, R.; Botti, A.; Bruni, F.; Ricci, M. A.; Soper, A. K. Hydration of Sodium, Potassium, and Chloride Ions in Solution and the Concept of Structure Maker/breaker. *J Phys Chem B* **2007**, *111* (48), 13570–13577.
(12) Jahn, D. A.; Wong, J.; Bachler, J.; Loerting, T.; Giovambattista, N. Glass Polymorphism in Glycerol-Water Mixtures: I. A Computer Simulation Study. *Phys Chem Chem Phys* **2016**, *18* (16), 11042–11057.
(13) Ahlrichs, R.; Bär, M.; Häser, M.; Horn, H.; Kölmel, C. Electronic Structure Calculations on Workstation Computers: The Program System Turbomole. *Chemical Physics Letters*. 1989, pp 165–169. https://doi.org/10.1016/0009-2614(89)85118-8.
(14) Perdew, J. P.; Wang, Y. Accurate and Simple Analytic Representation of the Electron-Gas Correlation Energy. *Phys. Rev. B Condens. Matter* **1992**, *45* (23), 13244–13249.
(15) Becke, A. D. Density-Functional Exchange-Energy Approximation with Correct Asymptotic Behavior. *Phys Rev A Gen Phys* **1988**, *38* (6), 3098–3100.
(16) Bucher, D.; Guidoni, L.; Carloni, P.; Rothlisberger, U. Coordination Numbers of K and Na Ions Inside the Selectivity Filter of the KcsA Potassium Channel: Insights from First Principles Molecular Dynamics. *Biophysical Journal*. 2010, pp L47–L49. https://doi.org/10.1016/j.bpj.2010.01.064.
(17) Bucher, D.; Kuyucak, S. Polarization of Water in the First Hydration Shell of K+ and Ca2+ Ions. *J. Phys. Chem. B* **2008**, *112* (35), 10786–10790.
(18) Lee, C.; Yang, W.; Parr, R. G. Development of the Colle-Salvetti Correlation-Energy Formula into a Functional of the Electron Density. *Phys Rev B Condens Matter* **1988**, *37* (2), 785–789.
(19) Perdew, J. P.; Ernzerhof, M.; Burke, K. Rationale for Mixing Exact Exchange with Density Functional Approximations. *J. Chem. Phys.* **1996**, *105* (22), 9982–9985.
(20) Adamo, C.; Barone, V. Toward Reliable Density Functional Methods without Adjustable Parameters: The PBE0 Model. *J. Chem. Phys.* **1999**, *110* (13), 6158–6170.
(21) Performance of the TPSS Functional on Predicting Core Level Binding Energies of Main Group Elements Containing Molecules: A Good Choice for Molecules Adsorbed on Metal Surfaces. American Chemical Society (ACS) 2020. https://doi.org/10.1021/acs.jctc.5b00998.s001.
(22) Staroverov, V. N.; Scuseria, G. E.; Tao, J.; Perdew, J. P. Comparative Assessment of a New Nonempirical Density Functional: Molecules and Hydrogen-Bonded Complexes. *J. Chem. Phys.* **2003**, *119* (23), 12129–12137.
