# Supplementary material for: Anomalous Water Fluorescence Induced by Solutes
Source: J Phys Chem Lett. 2025 Jun 30;16(27):6935–45. doi: 10.1021/acs.jpclett.5c01189 (PMC12257589; doi:10.1021/acs.jpclett.5c01189)
Supplement: Supplementary file 3 [file jz5c01189_si_003.pdf]

jz-2025-01189k.R1

Name: Peer Review Information for "Anomalous Water Fluorescence Induced by Solutes"

First Round of Reviewer Comments

Reviewer: 1

Comments to the Author

Villa et al. propose an important extension and generalisation of a previous works of the same group, by conducting a systematic study on the absorption and emission of wide range of solutes in aqueous solutions, finding interesting and systematic trends. In addition, they combine (TD)DFT simulations on 3 selected systems to shed light onto the character of low-lying states. The conclude that a) the low lying electronic states are connected to defects in the hydrogen bond network (HBN) b) the presence of solutes very generally increases the lifetime (of potentially emissive states) with respect to bulk water. The authors argue that the microscopic origin of this relies on the improved stiffness of the HBNs in presence of a solute.

Although some of the statements in the second part sounds speculative with respect to the theory and simulations provided here, the work appears to be sufficiently detailed and scientifically solid in the experimental part. This article can be published on JPC Letters, but some improvements on the text and several integration are required at least for the simulation part. A list of comments is provided below.

1) The amount of details offered to the reader concerning (TD)-DFT is not sufficient in my opinion. First, there is no clear mention of the criteria for the selection of the "representative structure".

More importantly, there is no mention of ground-state DFT: were the structures obtained from classical MD optimized at DFT level? (if not the authors should definitely comment on

this) How many states were used in TDDFT simulations? It is surprising the lowest energy transition is around

Besides a small paragraph in the SI, some of the information is in the SI Table captions, but still there should be a proper dedicated section.

2) DFT functional. It may sounds "boring", but especially because the authors have used BLYP we do need a minimal dicussion anywhere about this choice. Indeed, Table S6 is not referenced anywhere in the main. BLYP is a GGA functional, which choice needs to be justified since they are working on finite-size clusters and other options are available. Not to mention positioning of CT states: was a NBO analysis made at the other functionals listed in Table S6?

3)

The following sentence:

"According to natural bond order (NBO) populations, S1 excitation has an O→H CT

character. On average, 0.5-0.6 electrons are transferred from one oxygen atom,

with atomic orbitals contributing to the HOMO, to a certain number of hydrogen

atoms that belong to LUMO."

does not appear to be supported by any (supplementary) data/table/figure. If NBO analysis has been conducted on selected clusters, some extra details should be provided to the reader.

4)

"Based on this analysis, we describe this CT state as a

peculiar zwitterionic diradical (H2O)•+/(H2O)•- form"

same problem as above.

Addictional calculations may be needed to prove that there is a biradical character.

Especially because no demonstration is made for (3), this appears even more an assumption.

5)

I have a problem with the sentence:

"Moving from the $(H_2O)_{110}$ cluster models to bulk liquid water, our TD-DFT calculations allow us to infer that defects are continuously generated within the hydrogen-bond network during the bulk dynamics, producing transiently undercoordinated water molecules."

Why TD-DFT should give indications on the presence of defects in a cluster model vs bulk water?

And why should we deduce about presence of defects featured in the bulk based on the cluster model? DFT only suggests about the fact that frontier orbitals for the cluster are located on the cluster surface, which then may be correlated with defects.

6) Sentence:

"The S1 excitation involves HOMO→LUMO mono-electronic transition with average excitation energy equal to 268.8±13.9 nm."

A wavelength is not an energy!!

However, authors may move their discussion onto an energy-scale.

This will be beneficial for interpreting specific trends as such Figure 1 (intervals on wavelengths do not scale equally for example).

7)

Figures can be certainly improved.

E.g Figure 1 the color/marker could code the type of system instead of being random.

The caption is a bit "minimal": these are the wavelength values of maxima or bands or ... ?

8)

Do the authors have an hypothesis about the origin of the 3 emissive regions?

Some elements that would correlate with the hypothesis about the mechanism provided in the paper would be beneficial.

9)

Data for reproducibility. There is no statement about data provided. I think that would be a great added value if the authors could share data that allows a reproduction of results, e.g. relevant structures employed for simulations and/or input files.

10)

I would have appreciate a minimal discussion placing the (several) hypothesis done in this work onto a scientific context. For example how the simulations here compares with results for excited states of water or water-ion clusters (if any)?

Reviewer: 2

Comments to the Author

The authors present a combined experimental and theoretical study on the origins of anomalous fluorescence emissions of aqueous solutions of non-aromatic solutes. They measured fluorescence spectra of 21 solutions, and performed DFT calculations on the NaCl/water and glycerol/water systems in order to support their hypothesis of charge transfer from solute to solvent and hindered diradical recombination in water via librations as main reasons for the observed fluorescence.

The subject of the present study is of high scientific interest, and the manuscript therefore deserves publication in high-level journals such as JPCL. The experiments have been thoroughly performed in a systematic way, the results are described and discussed in detail. The spectroscopic data and the thermodynamic analyses of the activation energies for nonradiative fluorescence decay of various solutions supports the hypothesis of librational modes leading to diradical recombination being responsible for the observed effect.

From the theoretical side, the present work is at least in part a continuation of earlier work (references 24 and 25), the major advance being increased size of the water clusters used as models of the solvent. This reduces the urgency of a rapid publication in JPCL.

In the following I will list my concerns with respect to the theoretical modeling:

1. I did not find any description of the methods that have been applied, except "DFT" and "TD-DFT", and "B-LYP/TZVP" in the caption of figure 3. Since excited states with a significant charge-transfer contribution, which are in the focus of the present study, are not correctly described with standard GGA-based Kohn-Sham-DFT, a higher theoretical level, e.g. range-separated hybrid functionals or GW-BSE as mentioned in some references, is required.

It is therefore mandatory to include more computational details (including software, basis set etc.) and discuss possible effects of the method choice on the conclusions. If indeed GGA functionals such as BLYP were applied in the TD-DFT calculations, a critical comparison with higher-rung methods should be performed for the excited states of the water-solute clusters.

2. In this respect the accuracy of the DFT calculations should also be assessed by comparison of the calculated and measured fluorescence spectra. Also the calculated vibration frequencies, in particular those of the librations, should be compared to the present experiments.

3. I recommend to consider coupling of electronic and vibrational effects by calculating vibronic excitations. This would shed light on the accessibility of the libration modes.

4. Independent of the size of the water clusters, the results (e.g. for the frontier orbitals, figure 3) clearly show that the electronic structure, and subsequently the radical formation, is due to surface effects which are not present in a homogeneous water bulk. The authors admit that these effects exist and argue that their models correspond to real solutions where fractal-like voids exist (page 9).

The question is, how abundant these voids are, and if their concentration is large enough to explain the observed effects. Furthermore, the quoted works (72-74,78) are based on MD

simulations. Ref. 42 describes water-air and ice-air interfaces. Ref. 43, which is also a theoretical study, seems to be better suited as reference. The study should include an experimental confirmation of these theoretical models, either by own measurements or by quoting existing literature.

If the relevance of water-void interfaces cannot be demonstrated, it is indispensable to compare the present results to periodic TD-DFT calculations which are not prone to boundary effects.

Author's Response to Peer Review Comments:

Prof. Luca Bertini
Dipartimento di Biotecnologie e Bioscienze
Universita' degli Studi di Milano-Bicocca
Piazza della Scienza, 2
20126 Milano, Italy
Tel: +39 02 64483438 email: luca.bertini@unimib.it

Senior Editor
The Journal of Physical Chemistry Letters

Dear Editor

We would like to thank you for the valuable remarks and comments that helped us to improve our paper. We have carefully considered your requests and modified the paper accordingly. Hereafter we report a point-by-point response for each request/comment.

The original comments of the referees in black followed by our responses in blue. As a general note: all modifications to the original version of the main text, required to

fix issues raised or to detail comments made by the reviewers, have been highlighted in yellow in the revised version of the manuscript.

Luca Bertini on behalf of all coauthors

Reviewer: 1

Recommendation: This paper may be publishable, but major revision is needed; I would like to be invited to review any future revision.

Comments:

Villa et al. propose an important extension and generalisation of a previous works of the same group, by conducting a systematic study on the absorption and emission of wide range of solutes in aqueous solutions, finding interesting and systematic trends. In addition, they combine (TD)DFT simulations on 3 selected systems to shed light onto the character of low-lying states. The conclude that a) the low lying electronic states are connected to defects in the hydrogen bond network (HBN) b) the presence of solutes very generally increases the lifetime (of potentially emissive states) with respect to bulk water. The authors argue that the microscopic origin of this relies on the improved stiffness of the HBNs in presence of a solute.
Although some of the statements in the second part sounds speculative with respect to the theory and simulations provided here, the work appears to be sufficiently detailed and scientifically solid in the experimental part. This article can be published on JPC Letters, but some improvements on the text and several integration are required at least for the simulation part. A list of comments is provided below.

1) The amount of details offered to the reader concerning (TD)-DFT is not sufficient in my opinion. First, there is no clear mention of the criteria for the selection of the "representative structure".
More importantly, there is no mention of ground-state DFT: were the structures obtained from classical MD optimized at DFT level? (if not the authors should definitely comment on this) How many states were used in TDDFT simulations? It is surprising the lowest energy transition is around
Besides a small paragraph in the SI, some of the information is in the SI Table captions, but still there should be a proper dedicated section.

Thank you for your comment. Due to the word limit imposed on the main text of the paper, many of the computational details, including those related to the

- selection criteria for the representative structures from MD
- ground state DFT results ● TD-DFT details

are included in the Supporting Information. In particular,

- The energies and main characteristics of each structure optimized at the DFT level;
- The excitation energy for the first singlet excited S_1 state;
- A detailed account of the TDDFT relaxation on the S_1 PES, the main nuclear motions observed during the dynamics.

Aanswering point-by-point to this comment:

1. The structures subsequently studied at the DFT and TD-DFT levels were selected from a **classical molecular dynamics simulation of the cluster, treated as a single molecule**; the details are reported in the section computation of the Supplementary Materials.
2. The general criterion used to select representative structures from the MD simulation was to sample configurations in which the distance between solutes reflects the radial distribution functions reported in the literature;

3. In the TD-DFT calculations, only the first singlet excited state (S_1) was investigated. Therefore, **a single excited state was considered in each TD-DFT computation**.

Regarding point 2, the selection of solute/water structures was aimed at uniformly sampling geometries in which the distance between solutes falls within the range corresponding to the first three peaks of the radial distribution functions:

- 1 M NaCl, for Na–Na and Cl–Cl distances, the first peak is centered around 4 Å, while the second and third peaks are located around 5.5 Å and approximately 7 Å, respectively (reference 61 and 62 in the main text);
- In glycerol, the C2–C2 distance between the central carbon atoms of the two solute molecules shows a broad peak around ≈ 6 Å thus we consider a C2-C2 distance extending from approximately 4 Å to 8 Å.

In the figure below are reported these distances in the DFT optimized structures by re-ordering the plots in Figure 4C and 4F of the main text. While in the main text these figures show the distances sorted by increasing energy difference relative to the most stable structure, here they are instead sorted by increasing values of the distance under examination.

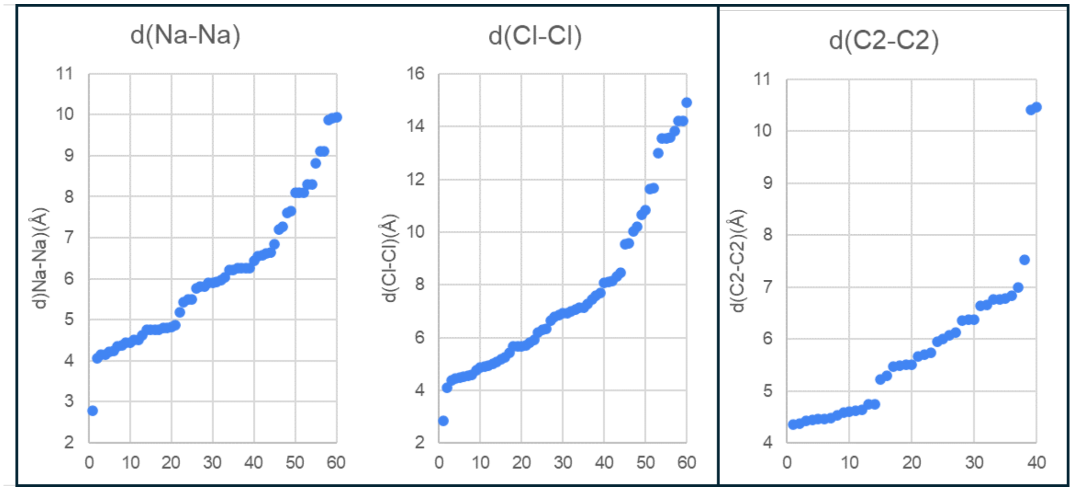

The range considered are therefore ● Na-Na: from 4 Å to 10 Å.

- Cl-Cl: from 4 Å to 15 Å.

- C2-C2: from 4 Å to 8 Å.

For example, in the case of NaCl, we selected structures with different ion arrangements from the classical dynamics, as highlighted in Figure 4 of the main text and in the Supplementary Materials (we reproduce the figures here below for the referee's convenience)

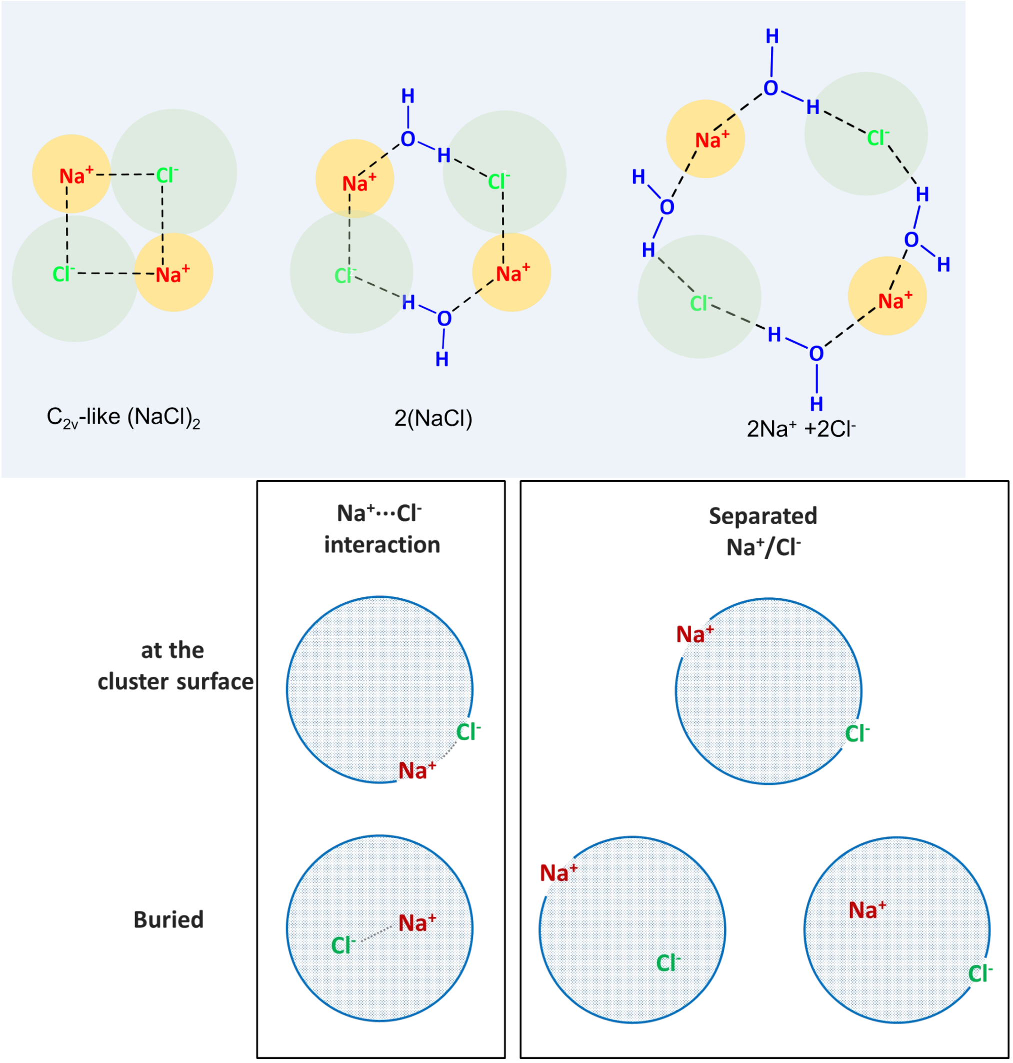

The two plots below show data from a 50 ns (time-step fs) molecular dynamics simulation of 1 M NaCl. The first plot reports the force-field-minimized energies of the structures identified during the simulation, while the second shows the Na–Na and Cl–Cl distances over the course of the trajectory.

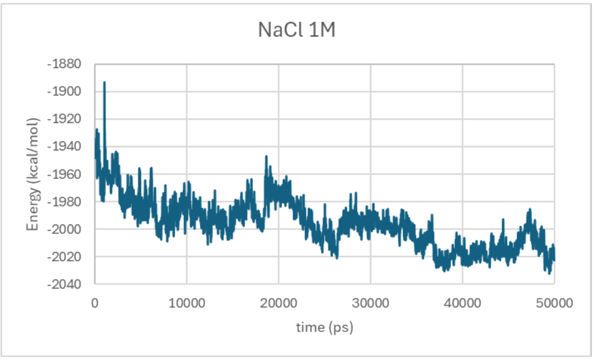

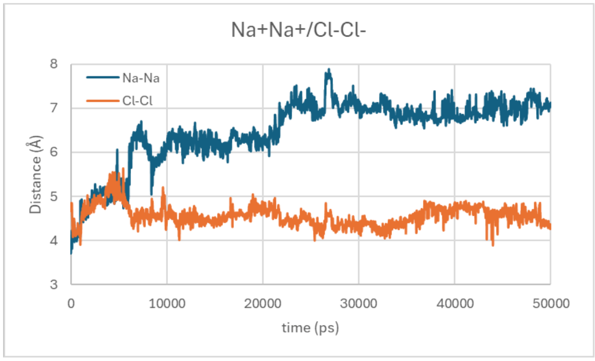

Similarly, we applied the same approach to the glycerol/water cluster, selecting structures based on different arrangements of the two solute molecules, as measured by the C2–C2 distance:

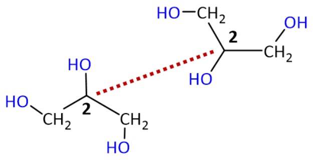

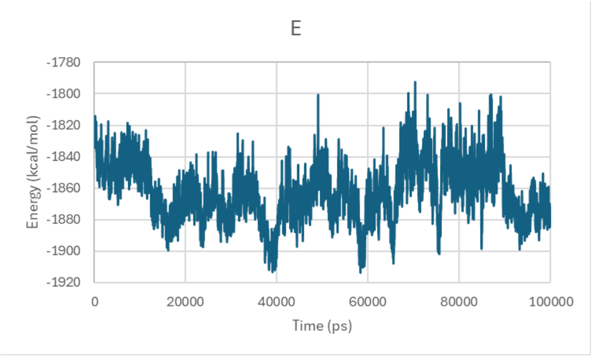

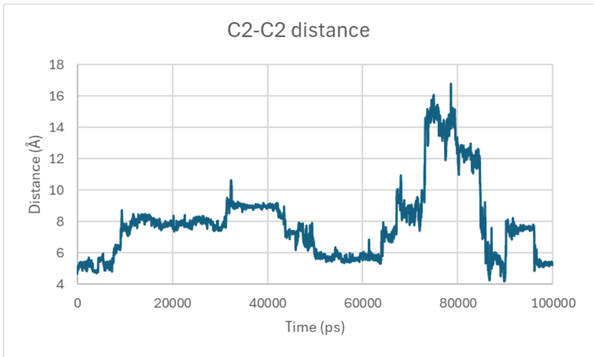

We observe that, both for NaCl and for glycerol, the considered structures uniformly cover the range of solute-solute distances observed in the radial distribution functions.

All these data have been included in the SI (computational at page 45).

2) DFT functional. It may sound "boring", but especially because the authors have used BLYP we do need a minimal discussion anywhere about this choice. Indeed,

Table S6 is not referenced anywhere in the main. BLYP is a GGA functional, which choice needs to be justified since they are working on finite-size clusters and other options are available. Not to mention positioning of CT states: was a NBO analysis made at the other functionals listed in Table S6?

We thank the reviewer for the comment concerning the choice of the exchange-correlation functional. Our selection of the BLYP functional was based on multiple considerations:

- In two previous studies conducted by our group, we compared experimental absorption spectra with TDDFT-calculated spectra using different functionals. We found that pure functionals, particularly BLYP, consistently provided a very good agreement with the experimentally observed absorption onset. In the figure below, we highlight the absorption spectra from Figure 1 of the main text, adding the average S_1 excitation wavelength computed at the TD-DFT level for the various structures considered. These values are in a good agreement with absorption onsets.

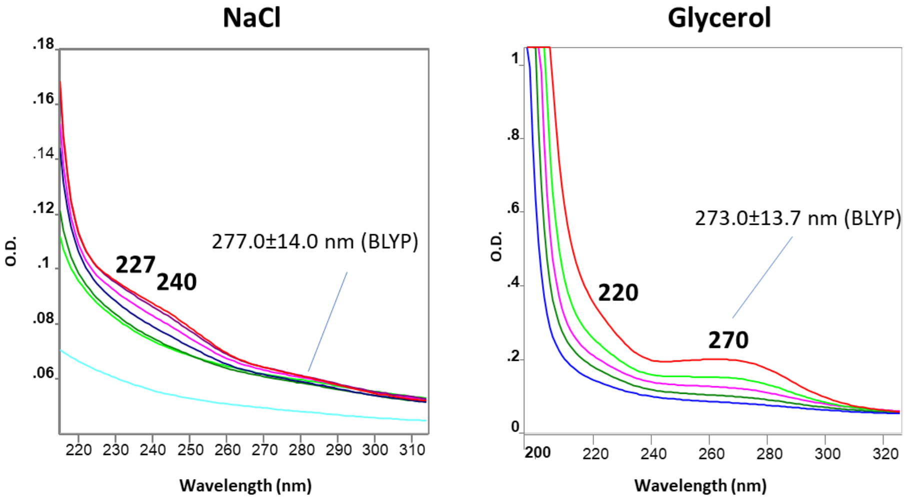

- The BLYP functional has also been shown to yield reliable results in ab initio molecular dynamics simulations involving ionic aqueous solutions. In particular, it accurately reproduces average ion coordination numbers in water and captures essential aspects of ion

hydration and salt dissociation, as demonstrated in previous studies (J. Phys. Chem. B 2008, 112, 10786–10790; J. Chem. Phys. 2010, 132, 114510). These findings further support our choice of BLYP for systems including K^+, NaCl, and $CaCl_2$ in aqueous environments.

- A further important practical motivation to adopt a pure GGA functional was the possibility of using the Resolution of Identity (RI) approximation within the TURBOMOLE suite to significantly accelerate the calculations—especially the excited-state geometry optimizations on the S_1 potential energy surface (see table S3-S5 where in column cycles are reported the number of TDDFT optimization steps performed for each structure considered). To adopt RI was crucial given the high computational cost of the present study. Indeed we considered 120 distinct structures each at TZVP basis set level. For example, each NaCl/water cluster included 328 atoms and 568 electrons, described using 3420 basis functions. The use of BLYP in combination with RI thus allowed us to make the extensive computational protocol tractable without compromising the reliability of the results.

We further extended the benchmarking of DFT functionals beyond the most stable structure of the glycerol/water cluster to $(H_2O)_{110}$ cluster and the $(NaCl)_2(H_2O)_{108}$ cluster. In this extended benchmark, we considered the same six functionals reported in Table S6 of the Supporting Information. For the referee’s convenience, we report these tables here below. ΔQ is the sum of the sum of the positive atomic charge differences comparing the S_0 state at its equilibrium geometry and the S_1 state at the S_0 equilibrium geometry.

**$(H_2O)_{110}$**										
**DFT functional**	**Total eneregy** (hartree)	**Dist O->H** CT (Å)	**ΔQ**			**n Hb**	**<O-H>± σ<O-H>** (Å)	**1ex**	**HOMO/ LUMO Gap** (au)	**S_1** (nm)
b-lyp	-8410.9525465	14,98	0,85			194	1,819±0,107	2O->H	0,179	253,9
b3-lyp	-8408.8198846	14,91	0,84			194	1,824±0,104	2O->H	0.259	184,5

pbe	-8403.7560768	14,71	0,82			195	1,757±0,113	2O->5H	0,185	248,1
pbe0	-8403.6194192	14,75	0,81			195	1,786±0,108	2O->6H	0,284	170,3
tpss	-8413.1087966	14,79				195	1,778±0,114	2O->6H	0,198	229,2
tpssh	-8412.1182330	14,96				193	1,793±0,114	2O->2H	0,235	197.5
**NaCl**										
**DFT functional**	**Total energy** (hartree)	**Dist O->H** CT (Å)	**ΔQ**	**d Na^+-Na^+** (Å)	**d Cl^--Cl^-** (Å)	**n Hb**	**<O-H>± σ<O-H>** (Å)	**1ex**	**HOMO/ LUMO Gap** (au)	**S_1** (nm)
b-lyp	-9503.2972	11,07	0.84	6.21	8.08	174	1,801±0,098	2O->6H	0.172	316,4
b3-lyp	-9501.0566	14,8	0,79	6,15	8,08	175	1,805±0,095	3O->6H	0,223	214,1
pbe	-9495.6240	10,79	0,83	6,11	7,92	173	1,730±0,101	2O->6H	0.147	310,0
pbe0	-9495.5993	10,78	0,82	6,13	7,95	174	1,744±0,101	2O->6H	0,148	308,9
tpss	-9505.49307	9,14		6,19	7,99	174	1,759±0,099	2O->6H	0,161	281,9
tpssh	-9505.4693	10,95		6,20	8,00	174	1,761±0,100	2O->6H	0,162	231,4
**Glycerol**										
**DFT functional**	**Total energy** (hartree)	**Dist C2-C2** (Å)	**Δq**			**n Hb**	**<O-H>± σ<O-H>** (Å)	**1ex**	**HOMO/ LUMO Gap** (au)	**S_1** (nm)
b-lyp	-8947,68138	4,455	0.77			209	1,850±0,158	3O->3H	0.167	272,1
b3-lyp	-8945,435232	4,444	0,84			209	1,852±0,152	3O->2H	0.249	192,0
pbe	-8940,034032	4,347	0.77			208	1,800±0,172	3O->4H	0.170	267,5
pbe0	-8939,927726	4,362	0.82			210	1,818±0,165	3O->2H	0.274	176,7
tpss	-8950,082921	4,398				209	1,815±0,174	3O->6H	0,185	245,8
tpssh	-8949,03569	4,401				209	1,823±0,164	3O->4H	0,273	206,6

We observed from these results that, across the various functionals considered, t**he nature of the first singlet excited state remains very similar**, as does the extent of CT. In general, we found that the charge transfer typically involves one or two atoms acting as donors and several hydrogen atoms (or Na atoms in the case of NaCl) as acceptors. As expected, the excitation energy exhibits greater variability in DFT calculations due to the inherent fluctuations in the HOMO–LUMO gap.

3)
The following sentence:
"According to natural bond order (NBO) populations, S1 excitation has an O→H CT character. On average, 0.5-0.6 electrons are transferred from one oxygen atom, with atomic orbitals contributing to the HOMO, to a certain number of hydrogen atoms that belong to LUMO." does not appear to be supported by any (supplementary) data/table/figure. If NBO analysis has been conducted on selected clusters, some extra details should be provided to the reader.

For each functional, **we evaluated the extent of charge transfer (CT) based on Natural Bond Orbital (NBO) charges, comparing the S_0 state at its equilibrium geometry and the S_1 state at the S_0 equilibrium geometry**. We considered all charge differences (ΔQ) exceeding 0.1 electrons as significant contributions to CT. This additional analysis provides further support for the trends observed in our main study and confirms the consistent behavior of the functionals across different solvation environments. Unfortunately we can not evaluate NBO charges for MGGA functionals (here TPSS and TPSSh) for excited state wave-function since this computation is not available in TURBOMOLE suite version 7.4.1.

We then took advantage of this helpful comment to further investigate the extent of charge transfer (CT) in the various structures we obtained. Specifically, we focused on the $(H_2O)_{110}$ cluster and considered all structures whose ΔE is lower than 10 kcal/mol. These results are also included in Table S6.

**$(H_2O)_{110}$ cluster structures whose ΔE is lower than 10 kcal/mol compared to the most stable form**							
**DFT functional B-LYP**	**Total eneregy** (hartree)	**ΔE** kcal/mol		**n Hb**	**<O-H>± σ<O-H>** (Å)	**1ex**	**S_1** (nm)
1	-8410.952546	0.0	0.85	194	1.819±0.107	2O→H	253.9
2	-8410.944911	4.8	0.83	190	1.809±1.111	2O→2H	292.1
3	-8410.944803	4.9	0.85	191	1.811±0.105	2O→H	272.7

4	-8410.944643	5.0	0.83	193	1.819±0.129	2O→3H	289.2
5	-8410.943713	5.5	0.85	191	1.813±0.109	2O→6H	268.4
6	-8410.940866	7.3	0.82	191	1.813±0.104	O→4H	275.2
7	-8410.938396	8.9	0.85	190	1.811±0.110	O→4H	253.7

Here, it is particularly important to note that we evaluated the charge transfer (CT) starting from the two oxygen atoms most involved in the S_1 state. As a result, compared to the initial estimate of 0.5–0.6 e, the CT increases to an average of 0.82 e in the $(H_2O)_{110}$ structures, as well as in those containing NaCl and glycerol, using different functionals. This point has therefore been revised accordingly in the main text.

As shown by the data reported in the table above, the S_1 exhibits similar characteristics among the seven most stable structures. In all cases, the excitation is characterized by a CT from one or two oxygen atoms toward hydrogen atoms belonging to water molecules located on the surface of the cluster

All these new data have been added in the updated Table S6 in SI.

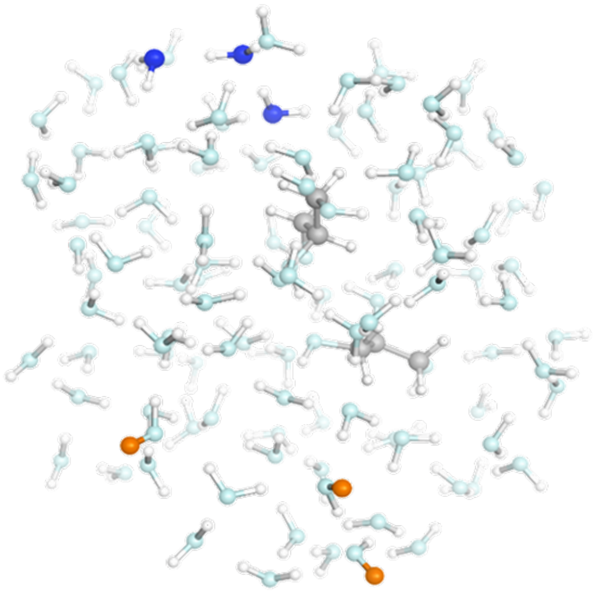

Lowest energy glycerol 1M model

In the figure provided above, the three oxygen donor atoms are shown in red, and the three hydrogen acceptor atoms are shown in blue for the most

stable water/glycerol cluster. In this case the mono-electronic excitation involve HOMO (590)->LUMO (591) transition where the largest orbital contribution are

MULLIKEN BRUTTO POPULATIONS FOR SELECTED MOS				
590a	energy/a,u,: -0,2148597			
atom	total	s	p	d
152o	0,66456	-0,00154	0,66583	0,00027
290o	0,11861	0,00033	0,11825	0,00003
302o	1,11856	-0,00395	1,1221	0,00041
591a	energy/a,u,:	-0,0474170		
atom	total	s	p	
174h	0,16387	0,16166	0,00221	
285h	0,18511	0,18248	0,00264	
306h	0,10867	0,10717	0,0015	

As a general comment, HOMO is mainly localized on a few oxygen atoms while

LUMO is more delocalized, as orbital hydrogen contributions show. These are the MO Mulliken population computed for the same structure as reported in figure 3 of the main text.

4)
"Based on this analysis, we describe this CT state as a peculiar zwitterionic diradical (H2O)•+/(H2O)•- form" same problem as above. Addictional calculations may be needed to prove that there is a biradical character. Especially because no demonstration is made for
(3), this appears even more an assumption.

Thank you for your observation. The assignment of the S_1 state as a zwitterionic diradical species is based on the nature of the main one-electron excitation characterizing this state. Specifically, in S_1, the dominant electronic transition involves an electron being promoted from the HOMO—

localized on the oxygen atom of a peripheral water molecule—to the LUMO, which is primarily localized on a dangling hydrogen atom. This spatial separation of the hole and the excited electron supports the interpretation of a CT character consistent with a $(H_2O)^{\bullet+}/(H_2O)^{\bullet-}$ pair. Most importantly, this assignment is based on the observation that vibrational relaxation on the S1 potential energy surface leads to the formation of the H_3O^{+}+ $OH^{\bullet}$ radical pair

(reference 54, Chipman, D. M. *Hemibonding between Water Cation and Water.* J. Phys. Chem. A 2016, 120 (48), 9618–9624). This result is clearly shown in Figure S72 in SI which we report here below for sake of convenience.

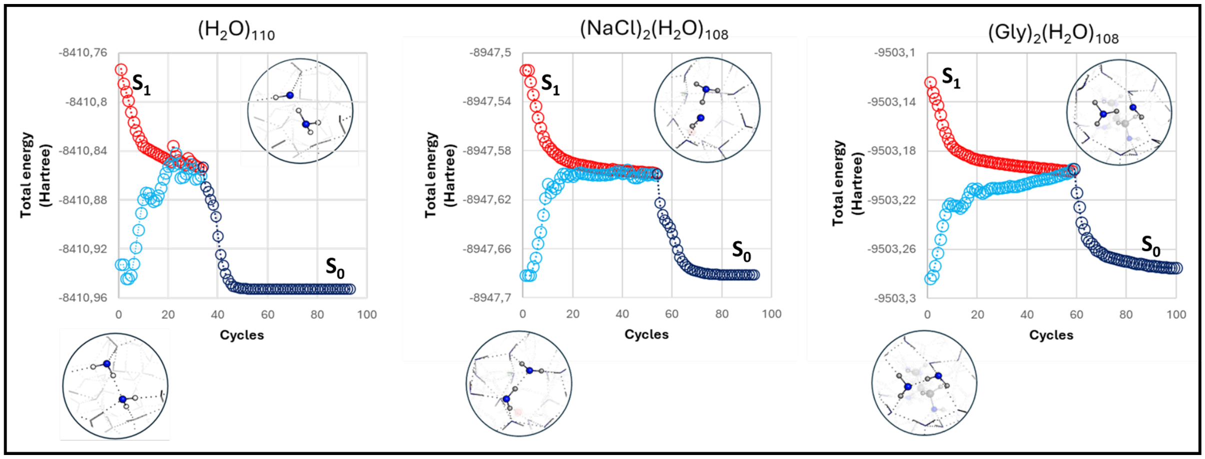

We agree that a more rigorous multi-reference approach would be needed to accurately evaluate the degree of biradical character. However, TD-DFT is able to qualitatively describe the nature of this electronic excitation and its evolution along the recombination coordinate on its PES.

In the main text the referee can find a sentence about this point which we report below with a small modification

> “This result supports our assignment of S_1 as a zwitterionic diradical-like species. Indeed this behavior is in line with the investigations[52,53] of the small cationic water cluster $(H_2O)_n$•+ in which the lowest

energy isomer is an hydrogen-bonded system $(H_3O)^+\cdots(HO)^\bullet$ while the hemibonded system $(H_2O\cdots H_2O)^{\bullet+}$ is higher in energy.[54]"

5)

I have a problem with the sentence: "Moving from the $(H_2O)_{110}$ cluster models to bulk liquid water, our TD-DFT calculations allow us to infer that defects are continuously generated within the hydrogen-bond network during the bulk dynamics, producing transiently undercoordinated water molecules."
Why TD-DFT should give indications on the presence of defects in a cluster model vs bulk water? And why should we deduce about presence of defects featured in the bulk based on the cluster model? DFT only suggests about the fact that frontier orbitals for the cluster are located on the cluster surface, which then may be correlated with defects.

We thank the reviewer for this insightful and justified comment. We agree that the original formulation in the manuscript was potentially misleading, as TD-DFT calculations on finite clusters cannot directly reveal the presence of defects in bulk liquid water.

We have therefore substantially revised the sentence in the main text as to clarify our intended meaning.

"Although TD-DFT calculations on finite clusters cannot directly capture the dynamics of bulk liquid water, our results suggest that molecules involved in CTTS transitions are often found in environments that deviate from ideal tetrahedral coordination. In the liquid phase, thermal fluctuations continuously lead to transient local structures with incompletely hydrogen-bonded water molecules. These molecules transiently involved in local environments that are not fully tetrahedral can act as potential donor–acceptor pairs contributing to charge-transfer bands in the absorption spectrum"

Our reasoning was not to infer defects in bulk water from the TD-DFT calculations per se, but rather to conceptually interpret the ordinary dynamics of liquid water—when imagined without surfaces, i.e., as a bulk

system. Here the transient nature of hydrogen-bond fluctuations continuously leads to local configurations that deviate from perfect tetrahedral order.

While the potential formation of nanovoids in liquid water has been thoroughly investigated by Dr. Ali Hassanali's group (as referenced in our manuscript), we do not claim that such voids are the sole or necessary origin of the anomalous fluorescence. Our argument remains valid even without invoking nanovoids. The intrinsic molecular motions of liquid water naturally give rise to spatially and temporally fluctuating structures. These fluctuations can transiently generate local environments that deviate from ideal tetrahedral coordination, allowing some water molecules to act as donor–acceptor pairs and contribute to charge-transfer bands in the absorption spectrum.

From this perspective, nanovoids may represent an additional source of structural inhomogeneities that can enhance or contribute to the CTTS band.

However reference 73 (*Charge Gradients around Dendritic Voids Cause Nanoscale Inhomogeneities in Liquid Water. J. Phys. Chem. Lett. 2022, 13, 7462*) investigate liquid water with femtosecond elastic second harmonic scattering (fs-ESHS) and molecular dynamics simulations, finding that transient cavities have lifetime ~ 300 fs and revealing that the total void fraction is approximately 3 vol %, with 20% of this void volume comprising structures larger than 1 nm. **These results suggest that nanovoids could indeed play a role in the emission process we observe experimentally.**

6) Sentence:
"The S1 excitation involves HOMO→LUMO mono-electronic transition with average excitation energy equal to 268.8±13.9 nm." A wavelength is not an energy!!
However, authors may move their discussion onto an energy-scale.
This will be beneficial for interpreting specific trends as such Figure 1 (intervals on wavelengths do not scale equally for example).

We thank the referee for pointing out the confusion regarding the terminology. We have revised the sentence accordingly and now refer to the excitation wavelength instead of excitation energy to ensure consistency and accuracy.

7)
Figures can be certainly improved.
E.g Figure 1 the color/marker could code the type of system instead of being random.
The caption is a bit "minimal": these are the wavelength values of maxima or bands or ... ?

According to the comment, we have revised the caption of Figure 1 to make it more informative and to clearly specify that the data refer to the maxima of the excitation and emission bands. The updated caption now reads:

“The comprehensive scatter plot displays the wavelength values for the maxima of the excitation and emission bands of all the aqueous solutions considered in this paper. The emission maxima clusterize in three spectral regions centered at 295 nm, 343 nm and 420 nm, while the excitation maxima clusterize in two regions centered at 228 nm and 323 nm.”

8)
Do the authors have an hypothesis about the origin of the 3 emissive regions? Some elements that would correlate with the hypothesis about the mechanism provided in the paper would be beneficial.

Thank you for the thoughtful question. Despite considerable effort, we must admit that the origin of the three distinct emissive regions is still not fully understood. At this stage, we do not have a definitive hypothesis that can confidently explain this observation. However, we fully agree that this is a key aspect deserving further investigation, and it is indeed one of the central questions guiding our ongoing and future studies. Specifically, we are currently investigating in detail the anomalous fluorescence properties of NaOH solutions, with a particular focus on identifying and characterizing at least several distinct non-radiative decay pathways that may contribute to the emission intensity maxima observed at different emission wavelengths.

9)
Data for reproducibility. There is no statement about data provided. I think that would be a great added value if the authors could share data that allows a reproduction of results, e.g.
relevant structures employed for simulations and/or input files.

In the SI we include the coordinates of the most stable geometries of $(H_2O)_{110}$,
$(NaCl)_2(H_2O)_{108}$ and $(C_3H_8O_3)_2(H_2O)_{108}$ clusters obtained from minimum search and optimized at B-LYP/TZVP level of theory.

10)
I would have appreciate a minimal discussion placing the (several) hypothesis done in this work onto a scientific context. For example how the simulations here compares with results for excited states of water or water-ion clusters (if any)?

We thank the referee for this constructive suggestion. Specifically, we have shown that the excited state most likely to decay radiatively is predominantly localized on water molecules that experience **transiently distorted** hydrogen-bonding environments, deviating from the ideal tetrahedral coordination. The core hypotheses of our work therefore concern the possible microscopic origin of such distorted environments (or “defects”). We propose two main scenarios: (i) these defects can naturally emerge during the ordinary thermal dynamics of bulk water, as also observed in previous simulations and experiments involving transient hydrogen-bond rearrangements; (ii) an additional, "extra" source of defects may arise from the formation of nanoscale voids or density fluctuations, which are known to affect the local structure of water and can enhance electronic localization.

Regarding previous literature, studies of excited states of liquid water and aqueous solutions have typically focused on small clusters due to computational limitations. As far as we are aware, our investigation of excited states of clusters of this size and complexity represents a unique contribution to the field. For example the reference 55 (Godinho et al. Charge Separation and Charge Transfer to Solvent in NaCl–water Clusters.

Chemical Physics Letters. 2004, pp 200–205) deals with $(NaCl)(H_2O)_n$ up to n=8, which is quite far from the nuclearity of our models. In reference 55 is observed for larger clusters with n≥5, the electronic density associated with virtual frontier MO are delocalized over the water molecules nearby the Cl-, indicating the formation of CTTS states in the clusters.

Here again, we would like to emphasize that our goal is not to reproduce experimental spectral data quantitatively, but rather to provide a model for the general mechanism underlying the emission phenomenon in these solutions. To this end, we study the properties of the first excited state using a model that approximates as closely as possible the behavior of the bulk liquid solution, within the limits imposed by the computational cost of the level of theory we can realistically employ. Such a model can only be a solute/water cluster, and the level of theory must necessarily be DFT with a pure functional, which allows us to take advantage of the RI (Resolution of Identity) method to significantly reduce the computational cost.

Additional Questions:
Urgency: High
Significance: High
Novelty: High
Scholarly Presentation: Moderate

Is the paper likely to interest a substantial number of physical chemists, not just specialists working in the authors' area of research?: Yes

Reviewer: 2

Recommendation: This paper is probably publishable, but major revision is needed; I do not need to see future revisions.

Comments:
The authors present a combined experimental and theoretical study on the origins of anomalous fluorescence emissions of aqueous solutions of non-aromatic solutes. They measured fluorescence spectra of 21 solutions, and performed DFT calculations on the NaCl/water and glycerol/water systems in order to support their hypothesis of charge transfer from solute to solvent and hindered diradical recombination in water via librations as main reasons for the observed fluorescence.
The subject of the present study is of high scientific interest, and the manuscript therefore deserves publication in high-level journals such as JPCL. The experiments have been thoroughly performed in a systematic way, the results are described and discussed in detail. The spectroscopic data and the thermodynamic analyses of the activation energies for nonradiative fluorescence decay of various solutions supports the hypothesis of librational modes leading to diradical recombination being responsible for the observed effect. From the theoretical side, the present work is at least in part a continuation of earlier work (references 24 and 25), the major advance being increased size of the water

clusters used as models of the solvent. This reduces the urgency of a rapid publication in JPCL.
In the following I will list my concerns with respect to the theoretical modeling:
1. I did not find any description of the methods that have been applied, except "DFT" and "TD-DFT", and "B-LYP/TZVP" in the caption of figure 3. Since excited states with a significant charge-transfer contribution, which are in the focus of the present study, are not correctly described with standard GGA-based Kohn-Sham-DFT, a higher theoretical level, e.g. range-separated hybrid functionals or GW-BSE as mentioned in some references, is required.
It is therefore mandatory to include more computational details (including software, basis set etc.) and discuss possible effects of the method choice on the conclusions. If indeed GGA functionals such as BLYP were applied in the TD-DFT calculations, a critical comparison with higher-rung methods should be performed for the excited states of the water-solute clusters.

The computational details have been included in the Supporting Information, in a dedicated section. This section contains the data for all the structures considered, as well as a validation of the level of theory, based on comparisons between BLYP results and those obtained using five other functionals: the pure GGA PBE, the hybrid functionals B3LYP and PBE0, the meta-GGA TPSS, and the hybrid meta-GGA TPSSh.

In brief the modeling was conducted using TURBOMOLE suite of programs,adoption using pure GGA B-LYP functional and triple-ζ plus polarization split valence quality (def-TZVP) basis set.

In the literature, most high-level quantum chemical computations focus on small water clusters. For instance, in the case of $(H_2O)_6$, Miliordos et al. (*J. Chem. Phys.*, 2015, 142, 234303) reported that the energy difference between the book and prism minima—where the prism is the global minimum—computed at the CCSD(T)/aug-cc-pVTZ level is 0.321 kcal/mol. At the BLYP/TZVP level, the corresponding difference is 0.456 kcal/mol. The discrepancy of 0.135 kcal/mol can be considered reasonably small.

In the literature, there are very few studies on the electronic excited states of large water clusters, and most of the existing research focuses on small clusters. At high levels of theory, including GW-BSE level, studies are mainly limited to small water clusters (1–6 molecules). Considering the paper by Blase et al. titled "*GW and Bethe-Salpeter study of small water*

clusters" (J. Chem. Phys. 2016; 144,034109.) we compute the B-LYP S_1 excitation energy for $(H_2O)_6$ in book conformation

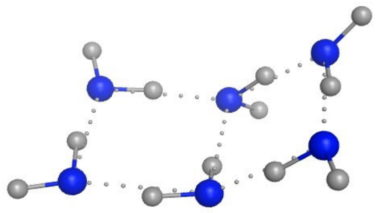

obtaining 6.01 eV that is in reasonable agreement with 6.24 eV at G_0W_0@PBE level.

Finally we would like to emphasize that our goal is not to reproduce experimental spectral data quantitatively, but rather to provide a model for the general mechanism underlying the emission phenomenon in these solutions. To this end, we study the properties of the first excited state using a model that

approximates as closely as possible the behavior of the bulk liquid solution, within the limits imposed by the computational cost of the level of theory we can realistically employ. Such a model can only be a solute/water cluster, and the level of theory must necessarily be DFT with a pure functional, which allows us to take advantage of the RI (Resolution of Identity) method to significantly reduce the computational cost.

2. In this respect the accuracy of the DFT calculations should also be assessed by comparison of the calculated and measured fluorescence spectra. Also the calculated vibration frequencies, in particular those of the librations, should be compared to the present experiments.

From this perspective, this represents a limitation of the model we employed. However, we would like to reiterate that the aim of this work is to provide a rationale for the various emissions observed experimentally, based on the nature of the S_1 excited state, which indeed decays radiatively to S_0 with a quantum yield of

0.010 ± 0.002. This means that, out of 100 molecules populated in the S_1 state, one decays via emission rather than through non-radiative pathways.

Therefore our goal is only to characterize the S_1 state populated in these solutions upon absorption, which often decays through non-radiative pathways but is also capable of radiative decay. From this perspective, the prediction of emission wavelengths lies beyond the scope of the present work

We consider the comparison of our TD-DFT computations on large solute clusters with the corresponding absorption spectra. In the figure below we report the detail of NaCl and glycerol absorption spectra at various concentrations as already reported in Figure 1 of the main text.

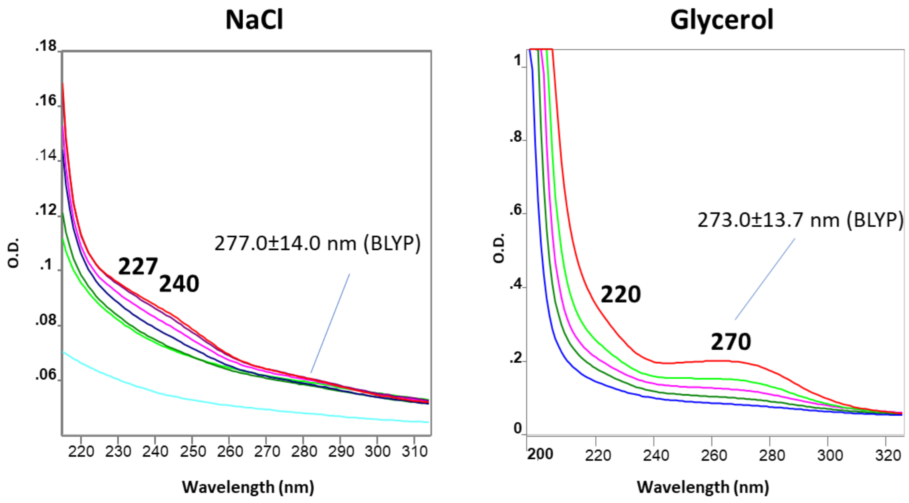

As shown in the figure, in both the case of NaCl and glycerol, the onset of absorption can be identified in the spectrum around 270 nm. This reasonably fits with the absorption wavelengths calculated for the S_1 state. In a previous investigation on KCl anomalous fluorescence carried out in our laboratory (Villa et al. J. Phys. Chem. Lett. 2019, 10 (22), 7230–7236) we computed the absorption spectra for 1M solution from the lowest energy for $(KCl)(H_2O)_{54}$ cluster computed at BLYP/TZVP level. Here we computed the excitation energies for the first 90 singlet excitations

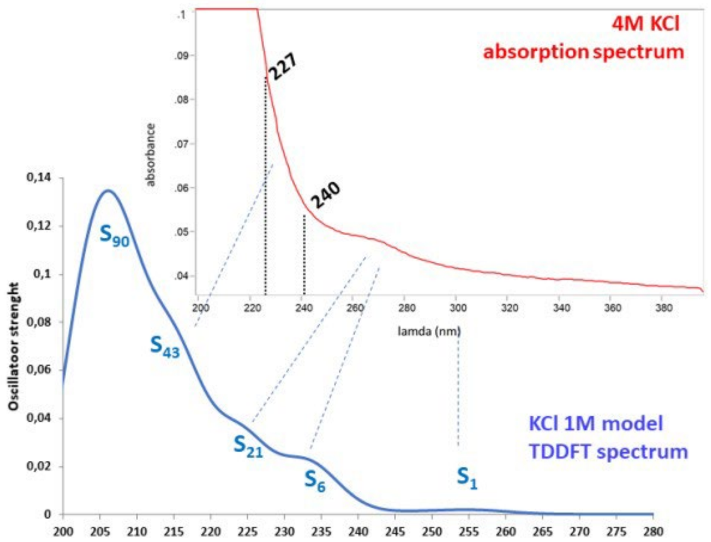

showing that the first weak shoulder measured at 260-270 nm is tentatively assigned

to the computed weak shoulder around 230-235 nm, which corresponds to S6 excitation.

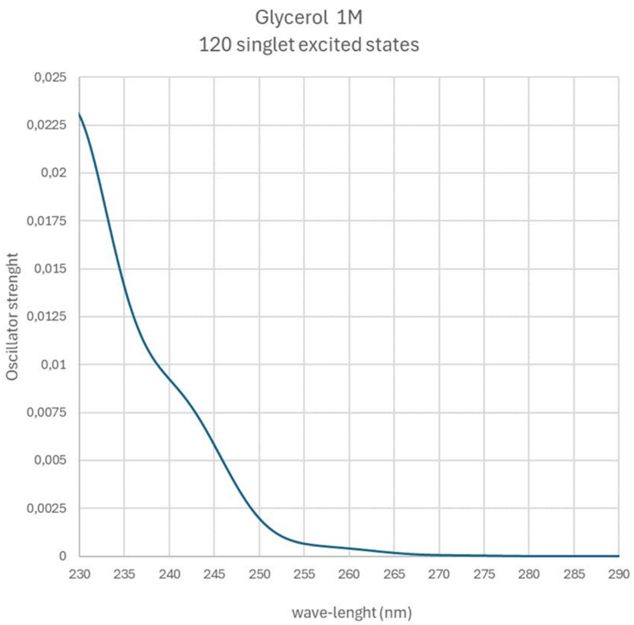

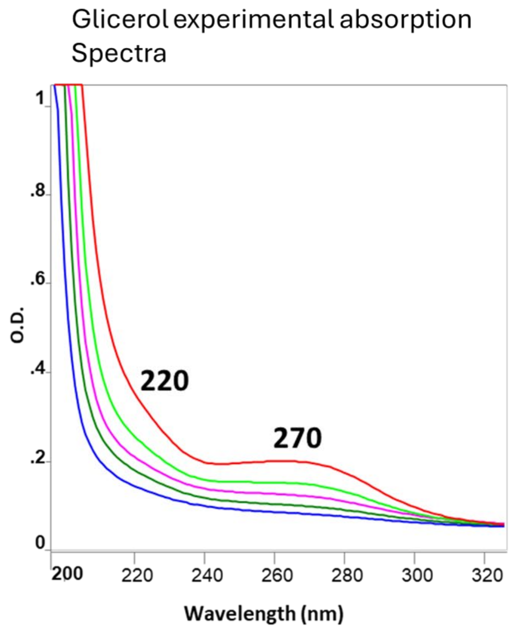

3. I recommend to consider coupling of electronic and vibrational effects by calculating vibronic excitations. This would shed light on the accessibility of the libration modes.

> We appreciate the referee's suggestion to consider the coupling of electronic and vibrational effects through the calculation of vibronic excitations. Unfortunately, our current computational framework (Turbomole suite) does not provide the tools necessary to perform such vibronic coupling calculations, which prevents us from addressing this aspect directly at this time.
>
> However, regarding the accessibility of the librational modes, we would like to note that our Arrhenius-plot analysis clearly indicates that these modes are thermally accessible. The system effectively utilizes them to facilitate non-radiative decay back to the ground state, as evidenced by the temperature-dependent behavior observed in our data.

4. Independent of the size of the water clusters, the results (e.g. for the frontier orbitals, figure 3) clearly show that the electronic structure, and subsequently the radical formation, is due to surface effects which are not present in a homogeneous water bulk. The authors admit that these effects exist and argue that their models correspond to real solutions where fractal-like voids exist (page 9).

The question is, how abundant these voids are, and if their concentration is large enough to explain the observed effects. Furthermore, the quoted works (72-74,78) are based on MD simulations. Ref. 42 describes water-air and ice-air interfaces. Ref. 43, which is also a theoretical study, seems to be better suited as reference. The study should include an experimental confirmation of these theoretical models, either by own measurements or by quoting existing literature.

If the relevance of water-void interfaces cannot be demonstrated, it is indispensable to compare the present results to periodic TD-DFT calculations which are not prone to boundary effects.

> The referee correctly points out that the electronic structure and radical formation in our simulations are predominantly influenced by surface effects, which are not representative of a homogeneous bulk phase. However, we would like to clarify and support the relevance of such interfaces in realistic aqueous systems.

First, we propose that defects that spontaneously arise during the normal thermal dynamics of bulk water are the centers of the CTTS states that feature transient hydrogen-bond non tetrahedral rearrangements. Then an additional source of defects might arrive from the formation of nanoscale voids.

Inhomogeneities of liquid water and nano-voids have been intensively studied at various levels. The question of how abundant these nanovoids are, has been focused in the reference 73 (*Charge Gradients around Dendritic Voids Cause Nanoscale Inhomogeneities in Liquid Water. J. Phys. Chem. Lett. 2022, 13, 7462*) where liquid water was investigated with femtosecond elastic second harmonic scattering (fs-ESHS) and molecular dynamics simulations (see Figure 3 of this paper). fs-ESHS measurements can be understood to arise from transient cavities with lifetime ~ 300 fs. From classical MD at 300 K these authors reveal that the total void fraction is approximately 3 vol %, with 20% of this void volume comprising structures larger than 1 nm. **These results further support the idea that nanovoids may be among the factors involved in the emission process observed experimentally**.
